# End processing in NHEJ by Polymerase λ and PNKP is coordinated during short-range synapsis

**DOI:** 10.1038/s41467-026-77503-2

**Published:** 2026-09-09

**Authors:** Alex Vogt, Andrea M. Kaminski, Jason G. Williams, Tasmin Naila, Danny Laurent, Alan E. Tomkinson, Susan P. Lees-Miller, Lars C. Pedersen, Thomas A. Kunkel, Yuan He

**Affiliations:** 1 Department of Molecular Biosciences, Northwestern University, Evanston, IL USA; 2 Interdisciplinary Biological Sciences Program, Northwestern University, Evanston, IL USA; 3 Genome Integrity and Structural Biology Laboratory, National Institute of Environmental Health Sciences, National Institutes of Health, Research Triangle Park, Durham, NC USA; 4 Epigenetics and RNA Biology Laboratory, National Institute of Environmental Health Sciences, National Institutes of Health, Research Triangle Park, Durham, NC USA; 5 Department of Internal Medicine, University of New Mexico, Albuquerque, NM USA; 6 Department of Molecular Genetics & Microbiology, University of New Mexico, Albuquerque, NM USA; 7 University of New Mexico Comprehensive Cancer Center, University of New Mexico, Albuquerque, NM USA; 8 Department of Biochemistry and Molecular Biology and Robson DNA Science Centre, Arnie Charbonneau Cancer Institute, University of Calgary, Calgary, AB Canada; 9 Department of Biophysics, Johns Hopkins University, Baltimore, MD USA; 10 Department of Biology, Johns Hopkins University, Baltimore, MD USA

**Keywords:** Cryoelectron microscopy, DNA

## Abstract

Non-homologous end joining (NHEJ) is a major pathway of DNA double strand break (DSB) repair, capable of directly joining both damaged strands of DNA through the coordinated activities of repair factors that detect the termini, physically bridge them together, and perform the chemistry necessary to complete repair. NHEJ is capable of repairing a variety of damaged DNA, employing various accessory end-processing factors to resolve chemically blocked ends, trim overhangs, and fill gaps in order to achieve directly ligatable DNA ends. To investigate the molecular mechanisms underlying end-processing, we determine the cryo-EM structure of the NHEJ specific polymerase Pol λ bound to the short-range synaptic complex, detailing the mode of its recruitment to the complex as well as a putative model for its activity. Furthermore, the coordinated end-processing activities of the short-range (SR) synaptic complex simultaneously bound by both Pol λ and PNKP, another accessory factor, demonstrates the ability of NHEJ to form large, multifunctional repair complexes capable of processing a variety of different DNA end structures to effect repair.

## Abstract (web summary)

Non-homologous end joining restores damaged DNA ends through a variety of enzymes that fill gaps, chemically cleanup termini, and ultimately ligate the DNA. Here, the authors show that the end-processing factors Pol λ and PNKP coordinate their activities with LigIV during short-range synapsis.

## Introduction

Double-stranded DNA breaks (DSBs) are the most lethal forms of DNA damage, requiring efficient and accurate repair for cells to retain viability and avoid carcinogenesis^1,2^. Non-homologous end joining (NHEJ) is the major DSB repair pathway in quiescent cells^3^, directly joining both strands of the damaged DNA through the concerted activity of pathway-specific factors, which can be roughly categorized into two groups: core NHEJ factors—those required in all instances of repair by NHEJ - and accessory factors, which are required only under certain circumstances^4^. Significant progress has been made in elucidating the molecular mechanisms that underly the activity of the core NHEJ machinery, supporting a multi-step model of repair in which the damaged DNA ends are detected and bound to by the Ku70/80 heterodimer^5^ followed by recruitment of DNA-PKcs^6^, before being brought into a long-range (LR) synaptic configuration mediated by the scaffolding proteins XRCC4, DNA Ligase-IV (LigIV), and XLF^7–9^. Autophosphorylation of DNA-PKcs in trans triggers a significant rearrangement of the kinase within the complex, precipitating its dissociation^10,11^ and a transition to short-range (SR) synapsis wherein the two copies of LigIV sequentially obtain access to the DSB to perform tandem ligation, resolving the DSB^7,12^.

Due to the variety of lesions produced by different DNA-damaging agents, additional chemical processing of the DNA ends is often a prerequisite for ligation to proceed^13^. This includes the activities of the NHEJ-specific DNA polymerases that fill short gaps and overhangs caused by a loss of nucleotides during damage or earlier stages of repair^14^, nucleases that truncate long overhangs as well as function in special cases of V(D)J recombination mediated by NHEJ^15^, and other DNA repair-related enzymes that resolve chemical lesions produced by damage which block direct ligation^16–18^. Single-molecule studies in *Xenopus* egg extracts indicate that at least certain end-processing events occur during short-range synapsis^19^, but the molecular details underlying these processes’ coordination with the core NHEJ machinery remain unclear, particularly in terms of the structural accommodation of the accessory factors within the large protein-DNA complexes that mediate repair.

Here we explore the activity of two different end-processing enzymes in the context of the core NHEJ machinery; DNA Polymerase λ (Pol λ), a family X DNA polymerase shown to function in NHEJ by filling gaps and overhangs a few (~4) nucleotides in length^20^, and Polynucleotide kinase-phosphatase (PNKP), a dual purpose repair enzyme that has kinase activity at 5′-hydroxyl (5′-OH) and phosphatase activity on 3′-phosphate (3′-Pho) DNA termini respectively^16^. We report the cryo-EM structure of the short-range (SR) synaptic complex with two copies of Pol λ bound, elucidating the mechanism of its recruitment to DSB. We also examine the activity of in vitro reconstituted complexes to show that both Pol λ and PNKP can cooperatively process the same DNA substrate within a single protein complex. Our results provide evidence for a model in which NHEJ end-processing occurs iteratively during short-range synapsis, with the core NHEJ factors forming a stable platform for the enzymes to flexibly access the DSB junction.

## Results

### Architecture of the Pol λ-NHEJ complex

**Fig. 1:**
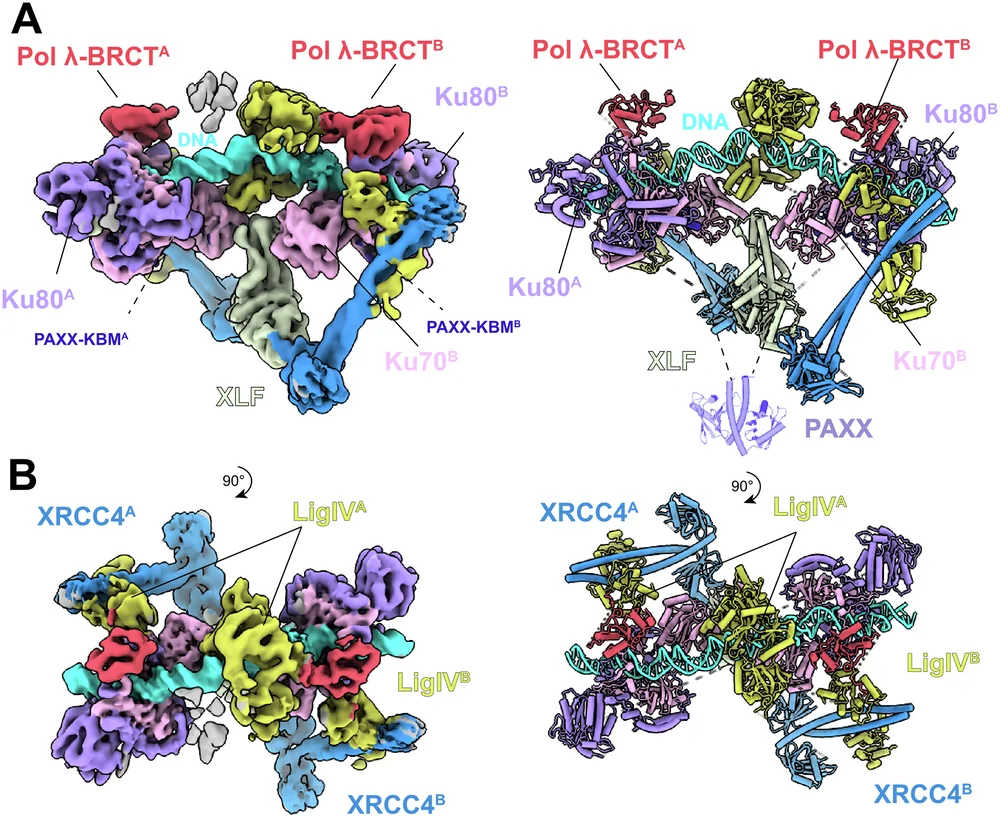
Structure of the SR-Pol λ NHEJ complex. **A** Composite cryo-EM map (left) and structural model (right) of the SR-Pol λ complex assembled on a DNA substrate containing symmetrical 1 nucleotide non-complementary 3′ overhangs. **B** Top views of the SR-Pol λ complex.

In order to capture an NHEJ synaptic complex in the presence of Pol λ, we extended the step-wise in vitro reconstitution of Ku70/80, XRCC4-LigIV, and XLF^7^ to include both Pol λ and PAXX, a redundant core factor that stabilizes the core NHEJ complexes through interactions with Ku70^9,10^, although its end-bridging activity is not wholly equivalent to XLF, with multiple investigations reporting substantially reduced or complete loss of ligation when the two factors are directly compared^10^. PAXX has been shown to physically interact with Pol λ through its globular head domain and stimulate its activity^21^. These factors were all sequentially added to a DNA substrate optimized for complex assembly that contained 1-nucleotide (nt) noncomplementary 3′ overhangs on either side to destabilize binding of LigIV’s catalytic domain and block direct ligation. The sample was then purified and stabilized using GraFix^22^ (Supplementary Fig. 1) and subjected to single-particle analysis by cryo-EM (methods). From roughly 170,000 particles, we obtained a 3D reconstruction with a reported resolution of 7.23 Å (Fig. 1A, B). This relatively modest resolution is in a range similar to that of the previously reported cryo-EM structure of the SR complex (8.4 Å)^7^, and is likely reflective of a degree of structural flexibility present in these states. Signal subtraction and local refinement of the XLF/XRCC4 scaffold, distal Ku70/80-Pol λ BRCT, and catalytic site-proximal Ku70/80-Pol λ BRCT respectively yielded reconstructions with nominal resolutions of 4.58 Å, 3.86 Å, and 4.43 Å (Supplementary Fig. 2), respectively, allowing us to clearly resolve secondary structure elements of both proteins and DNA.

The overall architecture of the SR complex is conserved in the presence of Pol λ, with the XRCC4-XLF-LigIV scaffold and both copies of Ku70/80 adopting almost identical conformations and orientations on the DNA (Fig. 1). Interestingly, more density attributable to XLF’s globular head domains was observed, possibly due to the improvement in map quality in this region compared to previous studies. We were able to model the loop between residues 85–92 (Supplementary Fig. 3A) unreported in the crystal structures, as well as extend the helix that leads into the flexible CTD by 5 residues (Supplementary Fig. 3B). Density corresponding to the DNA is noticeably sharper than reported previously, but the resolution still precludes precisely determining its register. PAXX’s Ku binding motif (residues 179–201) is also present between the core and vWA domains of Ku70 (Supplementary Fig. 3C), as observed in previous studies^9,10^, although its head and stalk domains do not appear in the reconstruction.

### Pol λ is recruited to the NHEJ complex through interactions with its BRCT domain and NTD regions

**Fig. 2:**
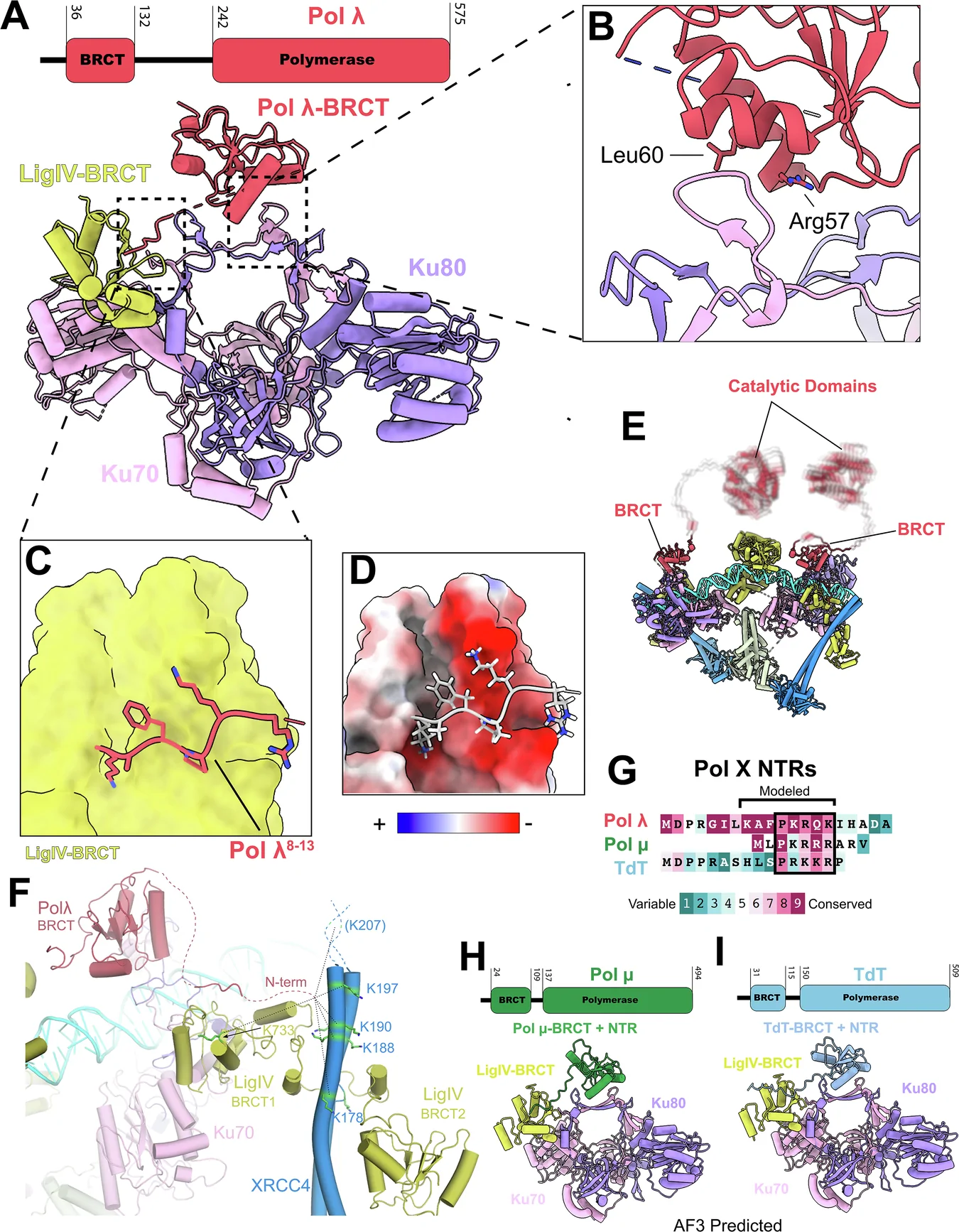
Pol λ recruitment to DSBs and catalytic activity is coordinated with the core NHEJ machinery. **A** Model of Pol λ’s NTR and BRCT domain interacting with Ku70/80 and LigIV-BRCT1 domain. **B** Magnified view of the BRCT-Ku70/80 interaction. The position of residues Arg57 and Leu60 are indicated**. C** Magnified view of the Pol λ NTR interaction with LigIV-BRCT1. **D** Magnified view of the Pol λ NTR interaction with LigIV-BRCT1 colored by electrostatic potential. **E** Model showing the flexible tethering of the polymerase domains to the SR complex. Full length AF3 predictions of Pol λ were used to accurately depict the length of the disordered linker region between the BRCT and polymerase domains. **F** Structural diagram of BS^3^ crosslinks involving the Polλ N-terminus, colored as in Fig. 1. Disordered linker regions are shown as dashed lines in colors coordinated by protein. BS^3^ crosslinks detected and identified by mass spectrometry are shown as black dashed lines, with the involved residues marked in green and drawn in stick. **G** Sequence alignment of Pol λ, Pol µ, and TDT with a conserved patch of basic residues following a proline indicated. **H** Domain architecture and AF3 prediction of Pol µ interacting with Ku70/80 and LigIV-BRCT1. **I** Domain architecture and AF3 prediction of TdT interacting with Ku70/80 and LigIV-BRCT1.

We observe two copies of Pol λ’s BRCT domain bound to the bridge region of Ku70/80 (Fig. 2A). Upon closer examination, the Pol λ BRCT domain straddles the bridge of Ku70/80 with connective density near residues Arg57 and Leu60 (Fig. 2B), which have been previously shown to be important for Pol λ’s recruitment to DSBs^23^. Given the known dependence of Pol λ’s activity on its BRCT domain^24^, this interaction is essential for anchoring the polymerase to the complex, allowing its catalytic domain more flexible access to the damaged DNA ends (Fig. 2E). Surprisingly, we also see evidence for an interaction between a portion of Pol λ’s N-terminal peptide and the LigIV C-BRCT1 which bridges XRCC4 and Ku70/80 (Fig. 2C, and Supplementary Fig. 3D). Guided by AlphaFold3^25^ (AF3) predictions of full-length Pol λ with Ku70/80-LigIV BRCT1, we place residues 8–15 at this position. This peptide comprises several highly conserved residues, including Phe10, which interacts with a hydrophobic patch on the BRCT’s surface, and a few positively charged residues that can form an interface with a nearby negatively charged region (Fig. 2D, G). This region has also been identified as the polymerase’s nuclear localization signal^26^, providing a striking example of the repurposing of conserved peptide regions.

To further investigate this interaction, we employed crosslinking mass spectrometry (XL-MS) (Supplementary Fig. 5A–C). This revealed a total of 17 crosslinks between the N-terminus of Pol λ and X4L4 (Fig. 2F). Two of these crosslinks were between K733 of the LigIV-BRCT1 subdomain, proximal to the putative binding terminus of the Pol λ-NTR. Surprisingly, a total of 11 crosslinks were observed between the Pol λ-NTR and the ordered C-terminal end of the XRCC4 coiled-coil region (K178, K188, K190, and K197). Another two crosslinks were observed between the N-terminus and K207 of XRCC4, located in the disordered tails. A large number of crosslinks in this area, combined with the fact that only one other was observed anywhere else in the entire NHEJ complex, strongly suggests that the Pol λ-NTR, upstream of its BRCT subdomain, follows the trajectory along the LigIV-BRCT1 subdomain, toward the XRCC4 coiled-coil. Interestingly, this strong pattern of crosslinks involving the Pol λ N-terminus is not observed when in complex with X4L4 alone, absent the rest of the NHEJ complex. Only one such crosslink was observed, with K190 of XRCC4. It is possible that interaction between the Pol λ-NTR is only observed in the context of the full complex, due to the slightly different orientation of the LigIV-BRCT1 subdomain, relative to the XRCC4 coiled-coil region, that are observed in the crystal structure of the LigIV tandem BRCT construct in complex with the head and coiled-coil domains of XRCC4^27^ (Supplementary Fig. 5D, E). In the crystal structure, the two LigIV BRCT subdomains are more extensively interacting with the XRCC4 coiled-coil region but flex slightly more open in the context of the complete NHEJ complex, generating new interfaces with both components of the Ku heterodimer. This may provide a structural underpinning for the N-terminal tail interaction with the polymerases—the BRCT1 subdomain’s interface with Ku may be required to present the optimal surface for stable binding.

Given the overlapping activities of the other family X polymerases, Polymerase µ (Pol µ) and terminal deoxynucleotidyl transferase (TdT), we performed a comparative analysis of AF3 predictions of each with Ku70/80-LigIV BRCT1. Both polymerases’ BRCT domains are predicted to bind in a nearly identical manner (Fig. 2H, I, and Supplementary Fig. 4A, B) to the bridge of Ku70/80, which is perhaps unsurprising given the structural similarities between these accessory domains. Curiously, regions of both Pol µ and TdT’s N-terminal region (NTR) are predicted to contact LigIV’s BRCT1 domain as well. Predictions between the LigIV-BRCT1 subdomain and all three polymerases’ NTRs are also suggestive of interaction (Supplementary Fig. 4C–E). Conservation analysis between all three polymerases’ NTRs reveals a patch of conserved positive residues following a conserved proline at residue 11 for both TdT and Pol λ, and residue 3 of Pol µ (Fig. 2G), although residues 8–10, which are represented in our EM map, are less well represented. Despite TdT and Pol λ both having longer NTRs than Pol µ, Pol λ’s is much more conserved and predicted to form a more extensive interface with the LigIV-BRCT1. It is unclear what role these differences in NTRs play, but given the nearly identical manner in which the BRCT domains appear to bind to Ku70/80, they may help modulate which polymerases are preferentially recruited to the complex through differences in affinity. It is also worth noting the differences in linker length separating the BRCT and catalytic domains of each polymerase, with Pol λ’s being significantly extended (110 residues) compared to Pol µ (18 residues) and TdT (35 residues), which may afford the former much more flexibility in its ability to access the DSB relative to the other family X polymerases.

### LigIV adopts an open conformation to allow end-processing at the DSB

**Fig. 3:**
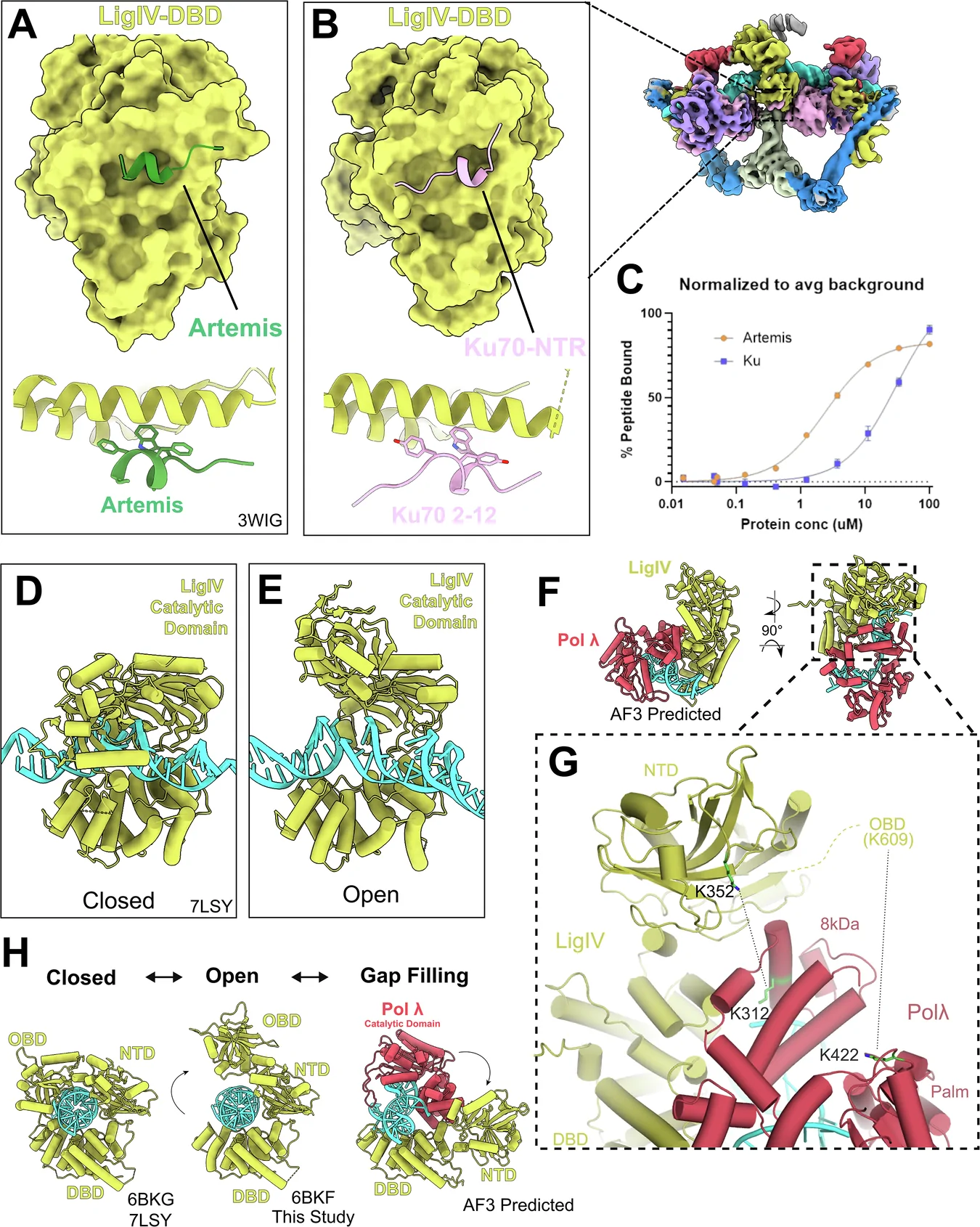
LigIV catalytic domain interacts with other NHEJ factors at the DSB. **A**, **B** Comparison of the binding location on the LigIV-DBD of both Ku70 and Artemis peptides with highlighted pattern of three aromatic residues at the LigIV-DBD binding interface shared by Ku70 and Artemis peptides below. **C** Fluorescence polarization assay comparing the binding affinities of the Artemis and Ku70 peptides interacting with the LigIV DBD. Data are presented as mean values ± SD of three biological replicates. **D**, **E** Comparison of the open and closed conformations of LigIV observed between the SR complex captured in this study and previously reported^7^. **F** AF3 predicted interaction between the catalytic domains of Pol λ and LigIV at the DSB. **G** Structural diagram of BS^3^ crosslinks between the Pol λ and LigIV catalytic domains mapped onto AF3 predicted model. BS^3^ crosslinks detected and identified by mass spectrometry are shown as black dashed lines, with the involved residues marked in green and drawn in stick. Disordered linker regions are shown as dashed lines in colors coordinated by protein. **H** Potential mechanism for Pol λ binding to the DSB based on reported structures of the LigIV catalytic domain and AF3 predictions of the Pol λ-LigIV-DNA complex.

To our surprise, we observed extra density near the bottom of LigIV’s DBD opposite its DNA-binding surface. Using AlphaFold3 to screen the factors present in the sample, we were able to identify and model a short portion of the Ku70 CTR at this position (Fig. 3B). Intriguingly, a short helix of the NHEJ-specific nuclease Artemis’ CTR binds in this exact position^28^, an interaction which has been shown to play an important role in V(D)J recombination^29^. A comparison of this interaction with the Ku70-LigIV interface reveals a pattern of three aromatic residues at conserved positions mediating the interaction (Fig. 3A, B). To compare the affinities of these two interacting peptides, we carried out fluorescence polarization on FAM-labeled Artemis^486-496^ and Ku70^1–11^ with the LigIV-DBD (Fig. 3C), and relatively modest K_D_’s were calculated for both interactions, with the Artemis-LigIV-DBD at 1.5–4 µM K_D_ and Ku70-LigIV-DBD at 29–30 µM. We speculate that there is some sort of handoff between these factors, perhaps with the Artemis interaction holding LigIV more distant from the DSB during long-range synapsis while Ku70 helps position it at the termini during short-range synapsis.

By supplying a DNA substrate with unpaired 1-nt 3′ overhangs, we sought to destabilize the LigIV catalytic domain’s interaction with the DSB. Intriguingly, we still observed the entire LigIV catalytic domain at the DSB, but in an open conformation that aligns well with that reported for LigIV bound to a non-ligatable substrate^30^. This is in contrast to the closed conformation observed in the previously reported SR complex (Fig. 3D, E). In this conformation, the DNA is primarily held by LigIV’s DBD, which serves a dual purpose in the complex by interacting with the vWA domain of the proximal Ku70 to stabilize short-range synapsis, with its NTD forming additional contacts with the DNA. Although the DNA substrate is discontinuous, the double helix is minimally distorted at the DSB junction, suggesting that LigIV is able to accurately align the ends even though they contain noncomplementary bases.

To better understand the mechanism by which Pol λ accesses the DSB junction, we used AF3 to predict potential interactions between the Pol λ catalytic domain, LigIV catalytic domain, and a gapped DNA substrate (Fig. 3F). Based on the available structural information, we hypothesized that the LigIV OBD and NTD domains need to open even further to accommodate Pol λ, potentially being completely displaced by the polymerase’s catalytic domain. This led us to compare predictions on the full-length LigIV catalytic domain and OBD or OBD+NTD truncations. In line with our hypothesis, the full-length LigIV catalytic domain is predicted to assume a slightly more open conformation, while deleting the OBD or OBD and NTD results in a prediction where both Pol λ catalytic domain and LigIV DBD form extensive interactions with the DNA flanking the gap (Fig. 3F). Furthermore, protein-protein interactions are predicted near the helix at 323–328 of Pol λ’s 8 kDA domain. Our XL-MS data corroborates the existence of such a state; five crosslinks are observed between the Pol λ catalytic domain and that of LigIV (4 between Pol λ K312 and LigIV K352 in the NTD and one between Pol λ K422 and LigIV K609 just outside of the OBD) (Fig. 3G). The K312-K352 crosslinks are consistent with our AF3 model of the Pol λ catalytic domain bound at the break site, while the K609 crosslink exists in a disordered region between the catalytic and BRCT domains of LigIV, which would position the OBD in an orientation possible in this state, too.

These results support a mechanism whereby the OBD and NTD domains of LigIV’s catalytic domain are displaced by the polymerase during gap filling, while the LigIV DBD maintains its position at the DSB, stabilizing the complex. Also supporting this notion of partial LigIV rather than complete release is the fact that the SR complex does not form when a LigIV construct containing only the BRCT domains is used to assemble the complex, even when the substrates contain terminal complementarity (Supplementary Fig. 6), which indicates Pol λ alone is not enough to stabilize short-range synapsis. Single-molecule studies show that mutating or completely deleting the LigIV DBD severely impacts the system’s ability to achieve synapsis^31^, providing further evidence that it may be irreplaceable in this aspect. While revising this manuscript, the related structure of Pol µ engaged with the short-range complex in precisely this configuration was reported^32^. In this structure, the polymerase’s catalytic domain displaces only LigIV’s OBD and NTD from the DSB, while the DBD remains in position to stabilize the complex. Given their semi-overlapping roles in NHEJ and their shared recruitment mechanism, it is unsurprising that these Pol X’s would also carry out end-processing in a similar manner.

### Pol λ allows ligation to proceed on gapped and overhang substrates

**Fig. 4:**
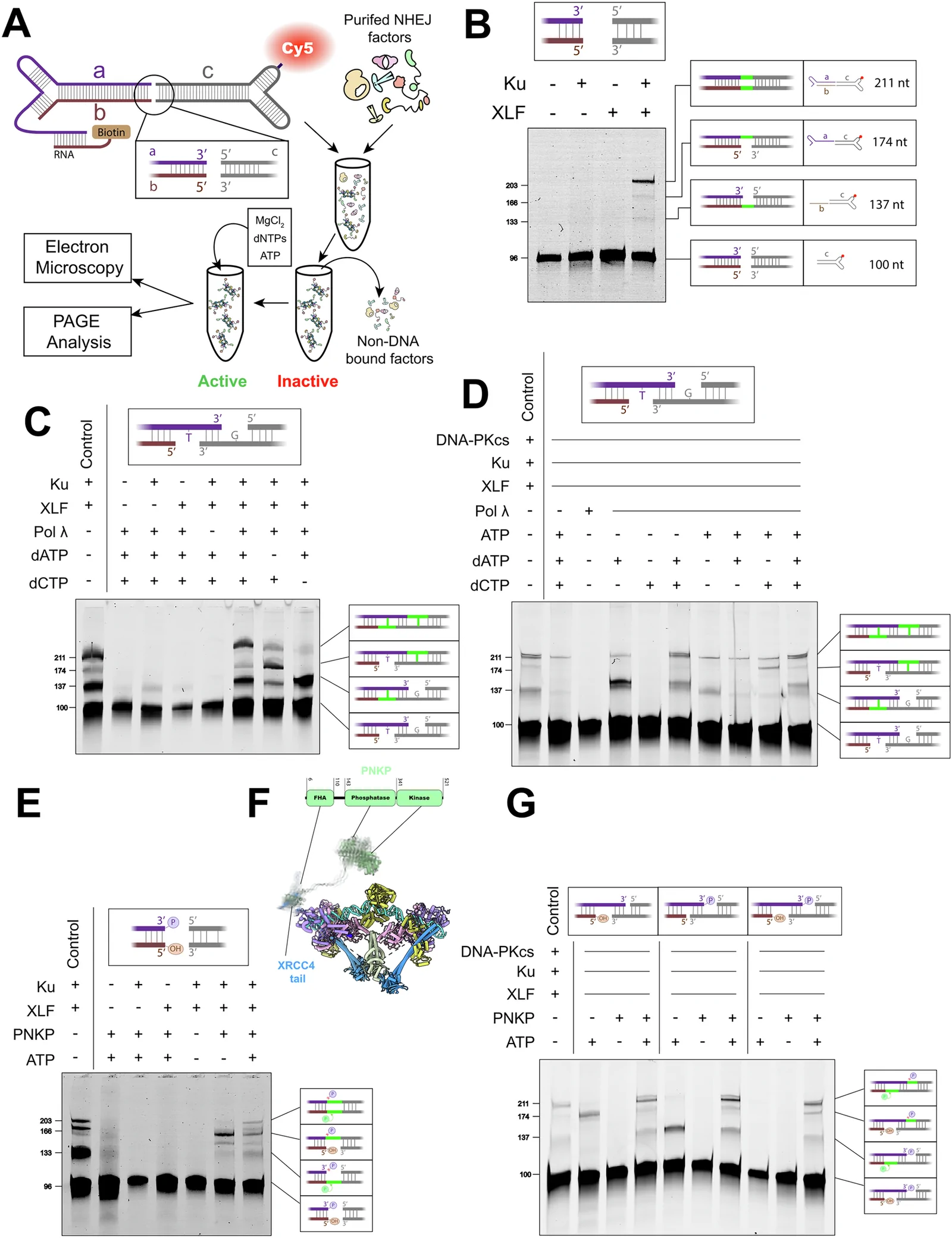
Pol λ and PNKP facilitate repair within larger NHEJ assemblies. **A** Schematic showing design of the generic DNA substrate used for complex assembly and protocol for assembly and ligation assay. **B** Denaturing TBE/urea gel analysis of end joining of a blunt-end DNA substrate by components of the SR complex. This and all subsequent reactions contain XRCC4-LigIV. Single and double ligation products are indicated on the right with each strand colored the same as in 3A and ligation events colored green. Uncropped gels are available in supplementary material. **C** Pol λ dependent ligation of a substrate with 3 base-pair microhomology flanked by single nucleotide gaps. Control reaction utilizes directly ligatable substrate with nicks separated by 4 bp terminal complementarity (C4). **D** Pol λ dependent ligation in the context of the LR complex demonstrating the use of dATP by DNA-PKcs for phosphorylation (lanes 2 and 4) and the incorporation of ribonucleotides by Pol λ (lanes 7–10). Control substrate is same as in 4C. **E** PNKP dependent ligation of a chemically blocked blunt end substrate. End-processing and ligation is dependent on the presence of all components of the SR complex + PNKP. Directly ligatable blunt end substrate is used as control. **F** Domain architecture of PNKP and a putative model for its flexible recruitment to the SR complex via the flexible tail of XRCC4. **G** PNKP dependent ligation proceeds in an ATP dependent manner in the LR complex. Single ligation proceeds at a nick opposite a 3’-Pho when the lesions are separated by a few base-pairs of complementarity (lane 5). Control substrate is same as used in 4D. All activity assays were performed in two independent biological replicates with similar results.

To functionally characterize the activity of Pol λ, we extended the ligation assay we used previously^7,10^ to include substrates with gaps and overhangs precluding direct ligation. The assembly of the complex and its enzymatic activities are separated by first adding components in a buffer free of Mg^2+^ followed by performing a pulldown on the biotinylated nucleic acid substrate to separate any non-DNA-bound proteins, and resuspending in a buffer containing Mg^2+^ to stimulate ATP hydrolysis, restoring the ligation activity of LigIV (Fig. 4a, and Supplementary Fig. 7A). Compared to assays where all components are mixed in a bulk reaction, this strategy allows for functional characterization of a more homogenous population of complexes by minimizing the number of freely diffusing factors unbound to DNA. This strategy has the added benefit that the sample is amenable to analysis by single-particle electron microscopy, allowing samples exhibiting robust ligation to be interrogated structurally to assay complex formation. For example, NS EM of a Pol λ containing pulldown with the components of the SR complex yielded a 3D reconstruction with clearly visible density corresponding to the Pol λ BRCT-Ku70/80 interaction (Supplementary Fig. 1B–E). We note that while this approach is particularly effective at capturing strongly interacting species, transient interactions that may also contribute to repair are likely to be excluded from the analysis.

**Fig. 5:**
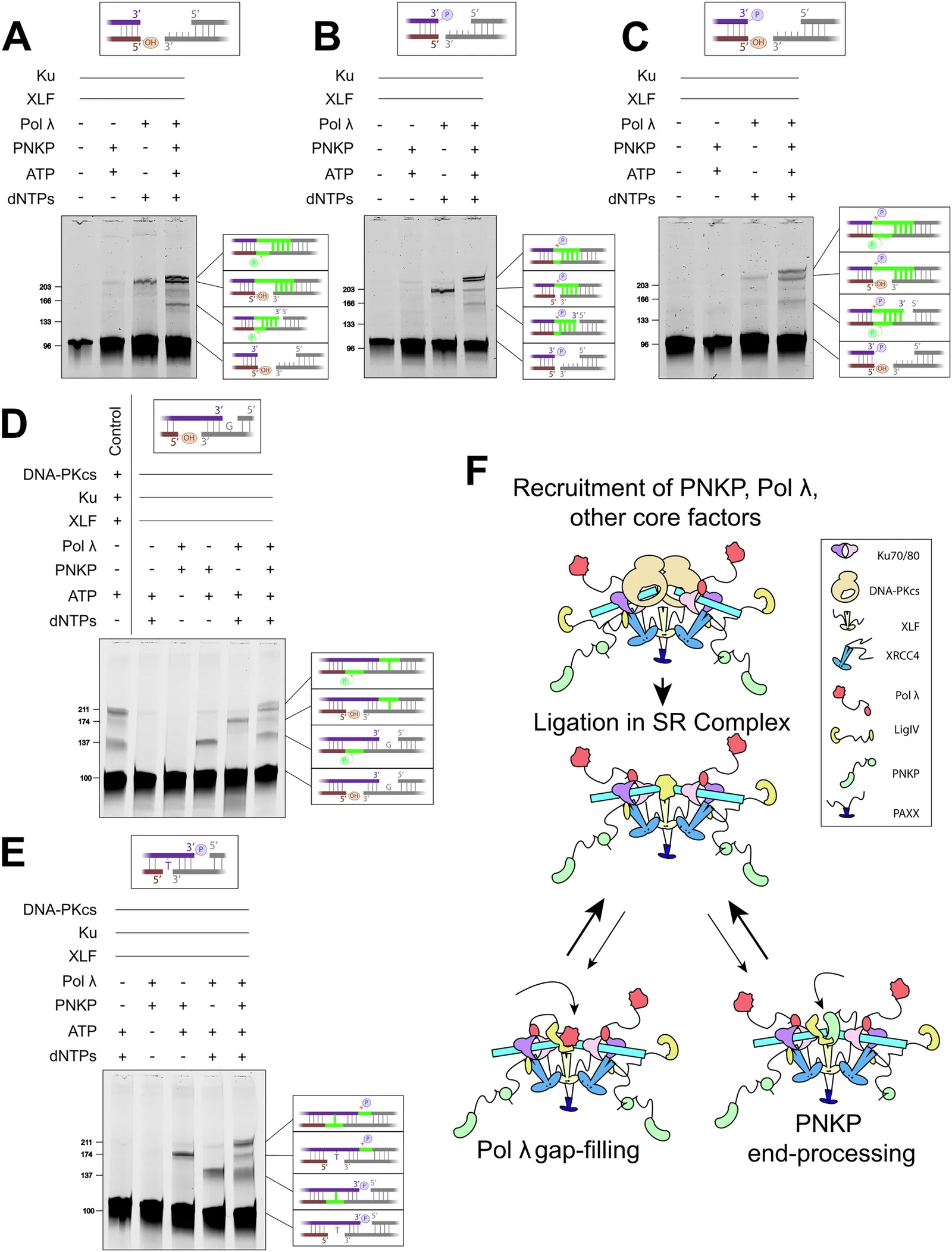
Coordinated DSB processing and repair by Pol, PNKP, and the core NHEJ factors. **A**–**C** Gap filling and end-processing dependent ligation of incompatible substrates containing 3′ 4 nt overhangs and 3′-Pho and/or 5′-OH. Pol λ appears to have robust activity on substrates with all lesions, although the full range of products are only generated when both factors are present. **D**, **E** Coordinated end-processing dependent ligation of gapped and chemically blocked substrates containing terminal complementarity in the LR complex. Distinct single ligation products dependent on either PNKP or Pol λ are observed (**D** lanes 2-3, **E** lanes 1,2), and double ligation products only seen when both factors are present. Control substrate is same as used in Fig. 4E. All activity assays were performed in two independent biological replicates with similar results. **F** Model for flexible end-processing in the short-range synaptic complex.

On substrates with blunt ends, all components of the SR complex (Ku70/80, XLF, and XRCC4-LigIV) make up the minimal functional unit capable of ligation (Fig. 4B). When 3′ and 5′ overhangs are introduced on one side, ligation is almost completely ablated, which is recovered by addition of Pol λ and free nucleotides (Fig. 4C, D). Little to no ligation of the strand opposite the overhang is observed in cases where Pol λ is absent (Fig. 4C lane 5, Fig. 4D lane 2), indicating that in this configuration LigIV is inhibited by any missing nucleotides, and Pol λ acts first to produce a blunt-end DNA termini prior to ligation. Surprisingly, ligation is also largely dependent on Pol λ in cases of microhomology flanked by gaps in this system with only faint gap-crossing ligation products visible in conditions absent Pol λ (Fig. 4C lanes 2-4). This stands in contrast to previous reports of more flexible LigIV substrate requirements^33^. We note several critical differences in our experimental approach that may account for these discrepancies, primarily the use of a pulldown to purify a relatively homogenous population of stable complexes. In this system, ligation is dependent on the presence of XLF (Fig. 4B), indicative of the formation of the SR complex, which holds the DNA ends in place more rigidly, possibly limiting its ability to accommodate structural distortions caused by ligation over gaps or bubbles. This is in contrast to the flexible synapsis observed in single molecule studies^34^, which is mediated by only Ku and XRCC4-LigIV and may afford more opportunities for LigIV to dynamically interact with the DSB and directly join a wider variety of substrates. In the absence of XLF, this flexibly synaptic state is likely too dynamic to survive pulldown by magnetic beads, further illustrating the differences between flexible and short-range synapsis. In our system, as opposed to substrates with no microhomology, single ligation on substrates with terminal base-pairing is possible (Fig. 4C), indicating that in the SR complex, LigIV can repair nicks near gaps independently of Pol λ activity, so long as they are flanked by base-paired nucleotides. We also note that the presence of double ligation products in the last two lanes of Fig. 4C where only a single nucleotide is supplied, suggests that Pol λ is capable of incorporating mismatched nucleotides, consistent with previous work identifying the “medium fidelity” inherent to family X polymerases^14^. Alternatively, it is possible that once one strand is repaired, LigIV becomes more capable of gap-crossing ligation, which would also be consistent with the presence of all ligation products observed in these lanes, although results on similar substrates in Fig. 5D, E do not show evidence of robust gap-crossing ligation after repair on the opposite strand.

Pol λ is also stably recruited to the LR complex (Fig. 4F, and Supplementary Fig. 6E). As described previously, the two copies of DNA-PKcs present in this configuration protect both DNA ends, and ATP must be supplied to this reaction for the kinases to autophosphorylate in trans allowing ligation to proceed (Supplementary Fig. 7B). When assembled on a gapped substrate, ligation by the LR complex is dependent on both ATP and Pol λ (Fig. 4D). Unexpectedly, ligation also proceeds when only dATP is added to a LR-Pol λ complex (Fig. 4D lanes 4 & 6), suggesting that DNA-PKcs can also utilize this cofactor for its kinase activity. To determine whether DNA-PK is active in the presence of dATP, purified DNA-PK was incubated under standard kinase assay conditions with equimolar amounts of either ATP or dATP (Supplementary Figs. 8A, B). The results show that DNA-PK is catalytically active and able to support DNA-enhanced autophosphorylation in the presence of either ATP or dATP on serine 2056, a highly conserved member of the PQR phosphorylation cluster of DNA-PKcs^35^. We also confirmed that this phosphorylation was sensitive to the DNA-PKcs inhibitor Nu7441 (Supplementary Fig. 8C). Although unusual, there are reports of kinases using dATP as a phosphate donor^36,37^, particularly in vitro, although we speculate this is of marginal relevance in the cell where ATP concentrations exceed dATP by orders of magnitude^38^. We also observe Pol λ utilizing ATP for gap filling of a gap with an unpaired thymidine (Fig. 4E lane 7), confirming previous reports of family X polymerases incorporating ribonucleotides during repair^39–41^. We further explored the ability of Pol λ to utilize different dNTPs and rNTPs when filling a single nucleotide overhang, detecting more ligation when the corresponding dNTP was supplied, although products of ligation were observed in the presence of all cofactors (Supplementary Fig. 6). These results are in agreement with previous reports of family X polymerases having medium fidelity, prioritizing restoration of the DNA ends to a directly ligatable configuration over error-free repair.

Structurally, the Pol λ BRCT-Ku70/80 and Pol λ NTR-LigIV BRCT1 interfaces are not occluded by DNA-PKcs in the LR complex, further indicating that Pol λ’s recruitment to the NHEJ complex is independent of its interaction with DNA and can occur during long-range synapsis. Complementing our findings, a recent study reported the Pol λ BRCT domain bound to the same position on Ku70/80 NHEJ complexes containing DNA-PKcs^42^, providing direct evidence for recruitment of the polymerase at earlier stages of repair. Separating Pol λ’s catalytic domain from the BRCT domain by a flexible linker region facilitates the decoupling of recruitment and enzymatic activity, thus allowing the formation of a functional complex without prematurely attempting chemistry at the DSB.

### PNKP is flexibly recruited to the NHEJ complex to repair chemically blocked DNA ends

To test whether the end-processing factor PNKP forms a similarly stable complex with the core NHEJ factors, we carried out in vitro reconstitution of the SR complex, separating the DNA bound species by both graFix (Supplementary Fig. 10B) and pulldown (Supplementary Fig. 10C). Although there was no marked peak shift in the graFix experiment, we observed that PNKP requires other NHEJ factors to be retained in the elution of a pulldown (Supplementary Fig. 10C), indicating that protein-protein interactions are responsible for its recruitment to the site of DNA damage during NHEJ. When subjected to single-particle electron microscopy, there were no noticeable differences between SR and SR-PNKP complexes in either 2D class averages or 3D reconstructions of negatively stained particles (Supplementary Fig. 10D–F). It is known that PNKP’s FHA domain interacts with the disordered tail of XRCC4 at the CK2-phosphorylated T233^43,44^, which flexibly tethers it to the complex^45^ precluding structural analysis by electron microscopy. PNKP’s domain architecture is similar to both LigIV and Pol λ; a flexible linker separates its catalytic domain from an accessory domain that interacts with components of the core NHEJ machinery (Fig. 4D). Therefore, it seems likely that it too is recruited to the complex independently of its direct interaction with the DSB.

3′-Pho and 5′-OH moieties chemically block ligation by the SR complex that the dual function of PNKP as a DNA phosphatase-kinase is capable of resolving (Fig. 4E). By adding PNKP after LigIV during complex assembly, we ensured that the end-processing enzyme was encountering a fully formed short-range complex, within which it would coordinate its activity. Substrates containing 5′-OH groups, both in blunt-end configurations and flanked by microhomology, show robust single ligation products at the compatible ends opposite the lesion, demonstrating that the 5′-OH does not inhibit LigIV activity on the opposite strand (lane 1 in Supplementary Fig. 9D, E, and Fig. 4E lane 2). However, 3′-Pho severely decreases ligation on the opposite strand (Supplementary Fig. 9F lane 2), indicating some disruption of the LigIV-DNA interactions necessary to facilitate ligation. The LR complex is also able to recruit PNKP (Fig. 4G), likely because the XRCC4-PNKP-FHA interaction is unperturbed by the presence of DNA-PKcs at the two DNA ends. Like the Pol λ-mediated end-processing, addition of ATP is required in all reactions for ligation to proceed in the presence of DNA-PKcs, due to the requirement of its phosphorylation and dissociation from the complex (Fig. 4G).

### End-processing by Pol λ and PNKP is coordinated during short-range synapsis

In cases of DNA damage where both nucleotides are missing and 3′Pho/5′OH groups block ligation, the activities of all three enzymes—LigIV, Pol λ, and PNKP—need to be coordinated for repair. Since all three factors form distinct interfaces with core NHEJ factors, there is no structural barrier preventing their recruitment to the same assembly, and we were able to efficiently assemble the entire complex (Supplementary Fig. 11). This SR-Pol λ-PNKP complex processes and ligates substrates containing both overhangs and chemically blocked ends, with double ligation only occurring in the presence of both end-processing factors (Fig. 5A–C). Unexpectedly, when assembled on a substrate containing a 3′-Pho and 4 nt 3′ overhang, Pol λ addition seems to produce substrates that are capable of a single ligation (Fig. 5B, C), although it is unclear how exactly this product is accounted for. In agreement with previous experiments, unpaired nucleotides in the form of overhangs limit ligation, which PNKP alone cannot recover. As expected, the LR complex is also capable of supporting both Pol λ and PNKP (Fig. 5D, E). Introducing microhomology between the gap and 3′-Pho or 5′-OH separates the sites of end-processing, allowing for distinct single ligation products resulting from either Pol λ or PNKP (Fig. 5D lanes 2, 3 and Fig. 5E lanes 1, 2), while the full range of products is only observed when both factors are present (last lane Fig. 5D, E).

## Discussion

The structure of the SR-Pol λ complex and biochemical evidence for the coordinated end-processing by Pol λ and PNKP support a model of NHEJ end-processing where the different factors are recruited at early stages of repair (i.e., pre-synapsis or long-range synapsis) followed by iterative enzymatic activity during short-range synapsis (Fig. 5F). This is consistent with studies showing the importance of the catalytically inert NHEJ polymerases’ BRCT domains and PNKP’s FHA domain in the factors’ ability to resolve DSBs^24,43^. This model is also strongly supported by the recent structural study reporting the Pol λ BRCT binding at the same position as Ku70/80 in the DNA-PK complex^42^. Interestingly, although these authors report structures with X4L4 bound to the DNA-PK-Pol λ complex, they did not observe the Pol λ NTR-LigIV BRCT1 interaction. Because the XLF-X4L4 scaffold has not been assembled in this long-range synaptic state, the torsional strain required to rotate the LigIV-BRCT domains observed during short-range synapsis is absent. This may support our speculation that a critical determinant in the polymerase’s NTR interaction is the transition to a short-range complex. Previous studies have also shown significant variability in both the positioning of XLF and XRCC4 relative to one another when forming the XXL (XLF-XRCC4-LigIV) scaffold, as well as the ability of the CTR of the XRCC4 coiled-coil region to fold back on itself under certain conditions^46,47^. This indicates some level of dynamism in this region, which is particularly intriguing given the XL-MS data showing significant interaction between Pol λ and XRCC4 near this site. We also highlight that this interaction, along with the Ku70 CTR-LigIV DBD interaction, adds to an expanding list of small interfaces and weak interactions between different NHEJ factors mediating synapsis and repair, in line with single-molecule studies demonstrating how the dynamic range of repair is quantitatively modulated by the presence or absence of different NHEJ factors^48^. Although we show that cryo-EM, particularly in conjunction with emerging structure prediction technologies, is effective at identifying and characterizing some of these interactions, there are undoubtedly limits to this strategy, and whether these approaches will prove useful in interrogating the effects of less ordered species such as certain lncRNAs that have been identified as NHEJ modulators^49^ remains an open question.

All three DNA enzymes described in our investigation (LigIV, Pol λ, and PNKP) contain accessory domains that anchor the protein to the complex and a DNA-binding catalytic domain responsible for performing the actual repair, both of which are separated by flexible linker regions. This domain organization decouples the factors’ recruitment to the complex from the chemistry they perform at the DSB, allowing the formation of a multifunctional complex with a variety of enzymes that can dynamically and iteratively access the DSB. Given the important structural role LigIV plays in stabilizing the SR complex, particularly its DBD, it is likely that its disassociation from the DSB is incomplete, consistent with a mechanism of coordinated repair whereby its OBD and NTD domains release the DNA and are displaced by the catalytic domain of another enzyme. As mentioned above, recently published work has demonstrated that this is indeed the case for Pol µ^32^, suggesting that this mechanism is conserved across the Pol X family in their function during NHEJ. It is interesting to note that while we were able to detect Pol λ sampling this configuration through XL-MS, we did not capture a population of particles by electron microscopy substantial enough to produce a 3D reconstruction with the polymerase’s catalytic domain bound to the DSB. This may suggest that Pol µ more stably occupies the site or binds to the LigIV-DNA junction with a higher affinity. It is also likely that substrate configuration is a potent modulator of these phenomena, as explored in our study and other recently published work^50,51^, and further investigation will be needed to fully understand how all three NHEJ-related Pol X’s modulate their activity relative to one another.

Our results provide mechanistic insight into a growing body of evidence that end-processing, particularly by the NHEJ polymerases and enzymes that resolve chemically blocked ends (PNKP, TDP, etc.), occurs during short-range synapsis in which both ends of the DSB are able to interact with one another. Single molecule studies of this system, including both Pol λ and PNKP, come to similar conclusions^19^, and crystal structures of Pol λ’s catalytic domains show extensive interactions with both upstream and downstream DNA possible only when both ends of the DSB have come close together^52^. Furthermore, prior to DNA-PKcs eviction, the DNA ends are sterically shielded by DNA-PKcs’ cradle, and access to the termini is occluded by the DBD-helix^7,11^, indicating that any enzymatic activity at the DNA ends would have to be well-coordinated with DNA-PKcs. Indeed, such pre-synaptic end-processing by the NHEJ nuclease Artemis has been extensively documented^15,53–55^, but the differences in the properties of this enzyme compared to the other NHEJ end-processing factors (dependence on phosphorylation by DNA-PKcs^56^, extensive interaction surface with DNA-PKcs^57,58^, unique substrate specificities^59^, and central role in V(D)J recombination^60,61^) suggest that its mode of activity may be distinct from other end-processing factors. In contrast, processing by Pol λ and PNKP has no DNA-PKcs requirement, and these factors are recruited independently of the kinase, although they are both targets of its phosphorylation^62,63^, the effects of which are currently not well understood. It is possible that limiting these more general end-processing activities to short-range synapsis could enhance fidelity by ensuring the formation of a complete and functional complex before releasing the DNA ends from the protection of DNA-PKcs’ cradle. Occluding access to the damaged termini until they are positioned and primed for close synapsis prevents the filling of any gaps or overhangs until any remaining microhomology is formed, thereby retaining as much original genetic information as possible. The preference for LigIV occupancy at the DSB also favors fidelity by ensuring that ligation is attempted often, resulting in sealing ends as soon as they become compatible, before extraneous end-processing can be attempted.

In summary, we provide structural and biochemical evidence for the coordinated activities of end-processing during NHEJ. Specifically, we identify interactions between Pol λ and the core NHEJ factors as well as demonstrate the functional cooperation of multiple DNA enzymes on the same DNA substrate in the same complex. By flexibly recruiting different factors to the same complex, NHEJ is able to repair a variety of DSBs while minimizing genetic aberrations. Future studies investigating the activities of other end-processing factors will be needed to determine if this mechanism of coordination is conserved and to test if the formation of even larger complexes capable of repairing a wider variety of DSBs is feasible. Continued dissection of the mechanisms underlying NHEJ’s ability to flexibly repair a variety of DSBs will help further understanding of the phenomena underlying responses to chemotherapy and radiation treatments, with opportunities to develop and optimize targeted therapeutic strategies.

## Methods

### Purification of core and accessory NHEJ protein factors

Nuclear cell pellets from 50, 100 or 300 L batches of logarithmically-growing HeLa cells were purchased from the National Cell Culture Center/Biovest International, Wisconsin, USA and purified as previously described^64^ with a measured concentration of 0.5 mg/mL. Previous approaches^65^ were also used to purify Ku70/80, LigIV-XRCC4, and LigIVΔcat-XRCC4 from baculovirus-infected SF9 insect cells (Expression Systems, cat. No. 94–001F) to final concentrations of 1.53 mg/mL, 7.9 mg/mL and 5.4 mg/mL respectively. Previous methods for XLF^66^, PAXX^65^, and PNKP^62^ expression and purification from *Escherichia coli* BL21-DE3 (Thermo Fisher Sciences No. EC0114) cells were used, resulting in concentrations of 2.0 mg/mL, 1.3 mg/mL, and 3.0 mg/mL respectively.

### Cloning, expression, and purification of full-length Pol λ

The sequence encoding full-length human Pol λ (Met1-Trp575) was cloned into the NotI/EcoRI restriction sites of the pGEXM vector^67^ to generate an N-terminal GST-tagged fusion protein. The plasmid was transformed into Rosetta2 (DE3) (Novagen) cells for expression, which was carried out in Terrific broth supplemented with 100 μg/mL ampicillin and 35 μg/mL chloramphenicol. Cultures were grown at 37 °C with shaking at 275 rpm to an OD_600nm_ of 0.8, when the temperature was reduced to 18 °C for 30 min. Protein expression was induced by the addition of isopropyl-β-D-thiogalactoside (IPTG) to a final concentration of 0.4 mM, and allowed to continue overnight. Bacterial cells were pelleted by centrifugation and lysed by sonication in 25 mM Tris pH 8, 750 mM NaCl, 5% (v/v) glycerol, and 1 mM DTT. The lysate was clarified by centrifugation and bound in-batch to glutathione Sepharose 4B resin (Cytiva). After extensive washing with sonication buffer to remove unbound proteins, the GST tag was removed by on-resin TEV cleavage in 25 mM Tris pH 8, 500 mM NaCl, 5% (v/v) glycerol, 1 mM DTT, rocking gently overnight at 4 °C. Cleaved protein was concentrated and further purified by size exclusion chromatography on a Superdex200 (Cytiva) column equilibrated with 25 mM Tris pH 8, 200 mM NaCl, 5% (v/v) glycerol, 1 mM DTT. Fractions containing the purified full-length Pol λ were pooled, concentrated to 4.71 mg/mL in chromatography buffer, and flash-frozen in liquid nitrogen.

### Complex assembly and GraFix

All oligonucleotide sequences used in this study are available in the Supplementary Data 1 file. Each was purchased from Integrated DNA Technology (IDT) and annealed as described previously^7^. Substrates were designed to include functional features used in previous investigations^7,10^, and altered primarily at the ends to include overhangs, gaps, 3′-Pho, and 5′-OH to serve as substrates for Pol λ and PNKP.

Complexes were assembled in a buffer containing 50 mM KCl, 10 mM HEPES pH 7.9, 5% glycerol, 1 mM DTT, 100 mM MgCl_2_, and 0.01% NP-40. For negatively stained samples and samples assayed for ligation, approximately 330 nM DNA, 350 nM DNA-PKcs, 430 nM Ku70/80, 1 µM XLF, 800 nM PAXX, 1 µM Pol λ, and 600 nM PNKP were added. Stepwise assembly of the complex was carried out by sequentially adding factors, followed by 5 min of incubation at room temperature after each step. First, Ku was added to each half of the DNA substrate, followed by DNA-PKcs where indicated. Both halves were then mixed together and incubated. Addition of XLF, XRCC4-LigIV, PAXX, Pol λ, and PNKP was carried out without incubating between factors, and the complex was allowed to equilibrate for 10 min at room temperature, followed by addition to the glycerol-glutaraldehyde gradient or addition of streptavidin-magnetic beads. In cases where GraFix was not used to purify complexes, a streptavidin-based pulldown was performed with 50 nM PAXX included during washing and elution steps where indicated to compensate for its rapid removal from the complex in this system, consistent with the protocol established in our previous work^10^.

GraFix^22^ was used to capture the SR-Pol λ complex. The sample concentration was increased roughly tenfold and the roughly 70 µL sample was then added to the top of a 2 mL 10–30% glycerol/glutaraldehyde gradient, prepared using a BioComp Gradient Master 108. After centrifugation at 76,974 × *g* for 16 h at 4 °C in a TLS-55 rotor using an Optima MAX-TL Ultracentrifuge (Beckman Coulter), the sample was split into 100 µL fractions, and the crosslinking reaction was quenched with 500 µM Tris-HCl. Non-denaturing PAGE revealed a peak corresponding to the Pol λ containing NHEJ complex in fractions 12–15, which were subsequently pooled and concentrated to a final volume of 20 µL using Amicron Ultra 0.5 mL centrifugal filters with a 100,000 MW cutoff (Millipore).

### Electron microscopy

Negative staining was carried out as described previously^7^. These samples were imaged using a JEOL 1400 transmission electron microscope equipped with a Gatan 4000 ×4000 charge-coupled device camera operating at 120 kV at a nominal magnification of 30,000× (3.71 Å/pix). Data were collected using Leginon^68^ and SerialEM^69^ with a defocus range of −1.5 µM to −3 µM.

Homemade graphene oxide (GO) grids^70^ were made for cryo-EM by applying GO to glow-discharged 2/1, 400 mesh Quantifoil grids that had been glow discharged using a Solarus plasma cleaner (Gatan). Grids were loaded into a Vitrobot IV (FEI) operating at 4 °C and 100% humidity, and 4 µL sample was added. Following 5 min incubation on the grid, the sample was blotted with a blot force of −10 for 3 s and plunge-frozen in liquid ethane before being transferred to storage in liquid nitrogen.

Two cryo-EM data sets of this sample were collected at the Pacific Northwest Center for cryo-EM (PNCC), totaling 14,845 movies from the same Titan Krios-3 transmission electron microscope (Thermo Fisher Scientific) operating at 300 kV equipped with a K3 direct electron detector (Gatan) and Biocontinuum energy filter with a 10 eV slit width set to 81,000× magnification (0.528 Å/pix in super-resolution mode). 3.162 s, 50-frame movies were collected with a total dose of 62 e^-^/Å^2^ and a defocus range of −1.5 µM to −4.0 µM.

### Image processing and 3D reconstruction

CryoSparc (v4.6)^71^ was used to process both negative stain EM and cryo-EM data. For negative stain, EM images were imported with constant CTF and directly subjected to blob picking, particle extraction, and 2D classification with a box size of 128 pix^2^. To generate the 3D reconstruction of the SR-PNKP complex from negative stain data, multiple iterations of 2D classification yielded 12,199 particles that were used to generate an ab initio model, which underwent homogenous refinement and B-factor sharpening prior to analysis.

For the SR-Pol λ cryo-EM datasets, movies were patch-motion corrected and CTF-corrected. Initial rounds of blob picking yielded 1,506,486 particles, which were extracted with a box size of 360 pix^2^ binned by four and subjected to multiple rounds of 2D classification to generate templates for a second round of picking. Template picking yielded 2,639,430 particles, which were extracted with the same box size added to the good particles from the initial round of picking (duplicates were removed). After multiple rounds of 2D classification, exemplary 2D class averages were used to generate low-resolution 3D reconstruction ab initio, which was then used as a reference for heterogeneous refinement of all 2,062,285 particles generated from template picking and remaining from blob picking. 4 “decoy” classes generated from discarded particles were also supplied in order to separate junk particles. Iterative 2D classification and heterogeneous refinement using both “good” and “junk” references were performed on particles classified into the particles sorted into the best class, which, after plateauing in quality following re-extraction at a box size of 416 pix^2^ retained 77,753 particles, resulting in an 8.71 Å reconstruction after non-uniform refinement. Exemplary 2D class averages of this particle set were selected, representing common and rare views of the complex, and these were used to train two Topaz^72^ models. Subsequent Topaz extraction yielded 392,215 particles, which were combined with the remaining particles from blob and template picking and passed through multiple iterations of 2D classification and heterogeneous refinement. Non-uniform refinement of a final set of 179,057 particles yielded a nominal global resolution of 7.23 Å.

Local refinement was carried out by splitting and masking the complex into three sections: the XLF-XRCC4 scaffold, the distal Ku-Pol λ BRCT-LigIV BRCT1 domain with the nearby DNA, and the proximal Ku + Pol λ BRCT-LigIV BRCT, LigIV catalytic domain with nearby DNA. UCSF Chimera^73^ and ChimeraX^74^ (made accessible through the SBGrid consortium^75^) were used for volume segmentation and mask generation. All particles were subjected to signal subtraction and locally refined using these masks, resulting in nominal resolutions of 4.58 Å for the scaffold, 3.86 Å for the distal Ku-Pol λ, and 4.43 Å for the LigIV catalytic domain + proximal components. Subsequent attempts at 3D classification did not improve the reconstruction.

### Model building

The published cryo-EM model of the SR complex^7^ (7LSY) was used as an initial homology model by rigid body docking into the newly determined cryo-EM map using ChimeraX. The PAXX KBM was placed by aligning the Ku70/80 heterodimer and PAXX KBM portions of the model from the LR-PAXX complex^10^ (8EZA). The AF3^25^ prediction of full-length Pol λ-Ku70/80-LigIV BRCT domains was used to position the Pol λ BRCT domain. AF3 predictions of the full-length XLF dimer and Ku70/80-LigIV were positioned in a similar manner, deleting portions where no density was observed. Web 3DNA 2.0^76^ was used to generate a generic DNA model of the 1 nt 3′ overhang substrate used for complex assembly, the termini of which were aligned to the nick in the catalytic site of LigIV. Following rigid body docking of all components, the model was manually rebuilt using ISOLDE^77^ and Phenix real-space refinement^78^.

### BS^3^ crosslinking analysis

All protein components of the NHEJ complex were buffer-exchanged into crosslinking buffer (20 mM HEPES pH 8, 100 mM KCl, 5 % (v/v) glycerol, 1 mM DTT). The NHEJ complex was assembled stepwise by first mixing Ku70/80 heterodimer with the Y35 1 nt overhang DNA substrate. After a 15 min incubation on ice, the X4L4 complex, XLF, and PAXX were added. After a subsequent 15 min incubation on ice, full-length human Polλ was added and allowed to bind for 25 min on ice. The final concentrations of each component are as follows: 2.37 μM DNA substrate, 1.77 μM Ku heterodimer, 2.36 μM X4L4, 1.77 μM XLF, 2.97 μM PAXX, and 2.96 μM Pol λ. 22 μL of assembled complex was mixed with 2.44 μL of 2 mM BS^3^ (Suberic acid bis(3-sulfo-N-hydroxysuccinimide ester, Millipore Sigma Cat # S5799) and incubated at room temperature for 15 min. 2.44 μL of 300 mM Tris pH 8 was added to quench the reaction. 13.5 μL crosslinking reactions of X4L4 and full-length Pol λ alone were carried out by mixing X4L4 and Pol λ (4 μM and 5 μM, respectively) and allowed to bind for 15 min on ice. 1.5 μL of 2 mM BS^3^ stock was added and incubated at room temperature for 15 min. The reaction was quenched by the addition of 1.5 μL 300 mM Tris, pH 8. Assembled NHEJ or X4L4/Pol λ complexes were used as negative controls for crosslinking. Sample processing, mass spectrometry, and data analyses were performed largely as described^79^, with the exception of the LC-MS data acquisition being performed using an Orbitrap Ascend mass spectrometer (ThermoFisher Scientific, Waltham, MA) coupled with a nanoVanquish UPLC system (ThermoFisher Scientific, Waltham, MA). Chromatography was performed with an Easy-Spray™ PepMap™ Neo 2 μm C18 75  μm × 150 mm analytical column and a PepMap™ Neo 5 μm C18 300 μm × 5 mm trapping column. 2 μL of the sample digest was injected onto the system where buffer conditions were initially held at 3% B for 5 min, then a linear gradient from 3% to 45% over 105 min. This gradient was followed by a step to 95% B and a 10-min hold. The flow rate was maintained at 350 nL/min throughout the analysis, with solvent A being 0.1% formic acid in water and solvent B being 0.1% formic acid in acetonitrile. The instrument was calibrated using Pierce™ FlexMix™ Calibration Solution (ThermoFisher Scientific, Waltham, MA). The mass spectrometer was operated in positive ion mode with a spray voltage of 2000 volts. Both MS1 (resolution of 120,000) and MS2 (resolution 30,000) analyzes were conducted using the Orbitrap analyzer, and Higher Collision Energy Dissociation (HCD) was used for fragmentation. Peaklists were generated using Mascot Distiller v 2.6.1.0 and searches were performed using the Batch-Tag Web tool from ProteinProspector (version 6.7.0) hosted by the University of California, San Francisco Mass Spectrometry Facility^80^.

### In vitro ligation assay

Activity assays were carried out similar to previously described^7,10^ and overall protocol is diagrammed in Fig. 4A. Cy5-labeled DNA substrates were annealed and NHEJ complexes were assembled through stepwise addition of the factors shown in a buffer containing 50 mM KCl, 10 mM HEPES pH 7.9, 5% glycerol, 1 mM DTT, and 0.01% NP-40. Samples were then incubated on streptavidin-coated magnetic beads for 15 min, following a wash in assembly buffer and resuspension in a reaction buffer identical to the assembly buffer with the addition of 5 mM MgCl_2_. Where indicated, NTPs and dNTPs were added at this step as well as a concentration of 1 mM, and the reaction was carried out at room temperature for 15 min except where indicated. Samples were then boiled at 95 °C for 5 min to end the reaction and denature proteins, followed by a 15 min proteinase K digestion of remaining DNA-bound factors at 37 °C. Formamide (50% volume) and 10 mM EDTA were added to samples, which were then boiled for 5 min at 95 °C before being added to a denaturing urea gel [TBE (tris-borate-EDTA) gel with 8% acrylamide (19:1)]. Gel electrophoresis was performed at 250 V for 30–40 min, and gels were scanned using a Sapphire Biomolecular Imager (Azure) at optimal absorbance.

### DNA-PKcs phosphorylation in the presence of dATP

Purified DNA-PKcs and Ku70/80 (each at a final concentration of 0.4 µM in a final volume of 20 µl) in 50 mM HEPES-KOH, pH 7.5, 10 mM MgCl_2_, 0.1 mM EDTA and 0.1 mM DTT were incubated in the presence of sonicated calf thymus DNA (10 µg/ml), an equivalent volume of TE and either 1 mM ATP or dATP at 27 °C for 3 min. Two separate sources of dATP were used, (a) ThermoScientific, cat #R0191 and (b) Invitrogen, cat #10216018. Reactions were stopped by the addition of SDS sample buffer, boiled and loaded onto low bis-acrylamide gels^81^. After electrophoresis at 100 V for approximately 90 min, proteins were transferred to nitrocellulose in the presence of SDS as described^81^. Membranes were probed for DNA-PKcs phosphoserine 2056 (Cell signalling, cat # 68716, 1:1000 dilution), total DNA-PKcs (monoclonal antibody 42–27, a kind gift from Dr. Tim Carter, St. John’s University, NY, 1:1000 dilution) and Ku80 (Cell Signalling Technologies, cat. #2180, 1:1000 dilution) as loading control. Separate gels were used for total DNA-PKcs and DNA-PKcs phospho-serine 2056.

### Cloning and purification of the LigIV DBD

The sequence encoding residues Met1-Ile240 was cloned between the NotI and BamHI restriction sites of the pET21M expression vector. This vector was transformed into Rosetta2 (DE3) cells for expression. These cells were grown at 37 C˚ in Terrific broth supplemented with 100 μg/mL ampicillin and 35 μg/mL chloramphenicol to an OD_600nm_of 0.8, when the temperature was decreased to 30 °C. Protein expression was induced by the addition of IPTG to a final concentration of 1 mM and allowed to continue for 4 h. The cells were pelleted by centrifugation, and the resulting cell pellets were frozen at −80 °C. Cell pellets were thawed, lysed by sonication in (50 mM Tris pH 8, 500 mM NaCl, 25 mM imidazole pH 8, 0.1% Tween-20, 5 mM βME), and clarified using centrifugation. His-tagged LigIV DBD was bound in batch to Ni-NTA resin, with rocking at 4 °C for 1 h. Unbound proteins were removed with repeated washes of lysis buffer, and the bound protein was eluted in-batch with lysis buffer supplemented with 300 mM imidazole pH 8. The N-terminal His-tag was removed by TEV protein digestion during overnight dialysis in 25 mM Tris pH 7.5, 150 mM NaCl, 2 mM βME. Imidazole pH 8 was added to the cleaved protein to a final concentration of 25 mM, and rebound to washed Ni-NTA resin. The cleaved protein was collected as flowthrough and further purified using size exclusion chromatography in 25 mM Tris pH 7.5, 150 mM NaCl, 2 mM βME. Fractions containing LigIV DBD were pooled, concentrated to 22.43 mg/mL and flash-frozen in liquid nitrogen.

### Fluorescence polarization assays for peptide binding to LigIV DBD

FAM-labeled Artemis^486-496^ (FAM-VPQWEVFFKRN) and Ku70^1-11^ (FAM-MSGWESYYKTE) peptides were synthesized by GenScript. These peptides were highly sensitive to oxidation and were therefore solubilized in 100% N-methyl-2-pyrrolidone (NMP), rather than DMSO. Fluorescence polarization assays were performed using 25 nM peptide at constant concentration, mixing with increasing concentrations of the LigIV DBD (5 nM–200 μM) in buffer (15 mM Tris pH 7.5, 65 mM NaCl, 0.7 mM βME). The complex was allowed to assemble for 20 min, before reading in 96-well black half-area plates, using a BMG CLARIOstar plate reader (ex. 482 nm/em. 520 nm filters). Gain adjustments were performed before each measurement using a control well containing FAM-labeled peptide alone. *K*_*d*_ values were then calculated in GraphPad Prism using nonlinear regression analysis of fluorescence polarization changes as a function of increasing LigIV DBD protein concentration.

### Reporting summary

Further information on research design is available in the Nature Portfolio Reporting Summary linked to this article.

## Supplementary information

Supplementary Information

Description of Additional Supplementary Files

Supplementary Data 1

Reporting Summary

Transparent Peer Review file

## Source data

Source Data

## Data Availability

Cryo-EM density maps have been deposited in the Electron Microscopy Data bank (EMDB) under accession numbers EMDB-70958 [https://www.ebi.ac.uk/emdb/EMD-70958] (consensus SR-Pol λ complex), EMDB-70960 [https://www.ebi.ac.uk/emdb/EMD-70960] (composite map SR-Pol λ complex), EMDB-70957 [https://www.ebi.ac.uk/emdb/EMD-70957] (XLF, XRCCC4, LigIV-BRCT scaffold), EMDB-70959 [https://www.ebi.ac.uk/emdb/EMD-70959] (distal Ku70/80, Pol λ-BRCT), and EMDB-70949 [https://www.ebi.ac.uk/emdb/EMD-70949] (proximal Ku70/80, Pol λ-BRCT, LigiV-catalytic domain). Model coordinates for the SR-Pol λ complex have been deposited in the PDB under accession number 9OX8. The raw mass spectrometry data are available at MSV000101839 [https://doi.org/10.25345/C5D795R00]. AlphaFold3 models of TdT and Pol µ interacting with Ku and LigIV are available at ModelArchive with the accession codes ma-wmbxe [https://modelarchive.org/doi/10.5452/ma-wmbxe] and ma-drktp [https://modelarchive.org/doi/10.5452/ma-drktb], respectively. Source data are provided with this paper.
