# Supplementary material for: End processing in NHEJ by Polymerase λ and PNKP is coordinated during short-range synapsis
Source: Nat Commun. 2026 Sep 9;17:10645. doi: 10.1038/s41467-026-77503-2 (PMC13649775; doi:10.1038/s41467-026-77503-2)
Supplement: Supplementary file 1 [file 41467_2026_77503_MOESM1_ESM.pdf]

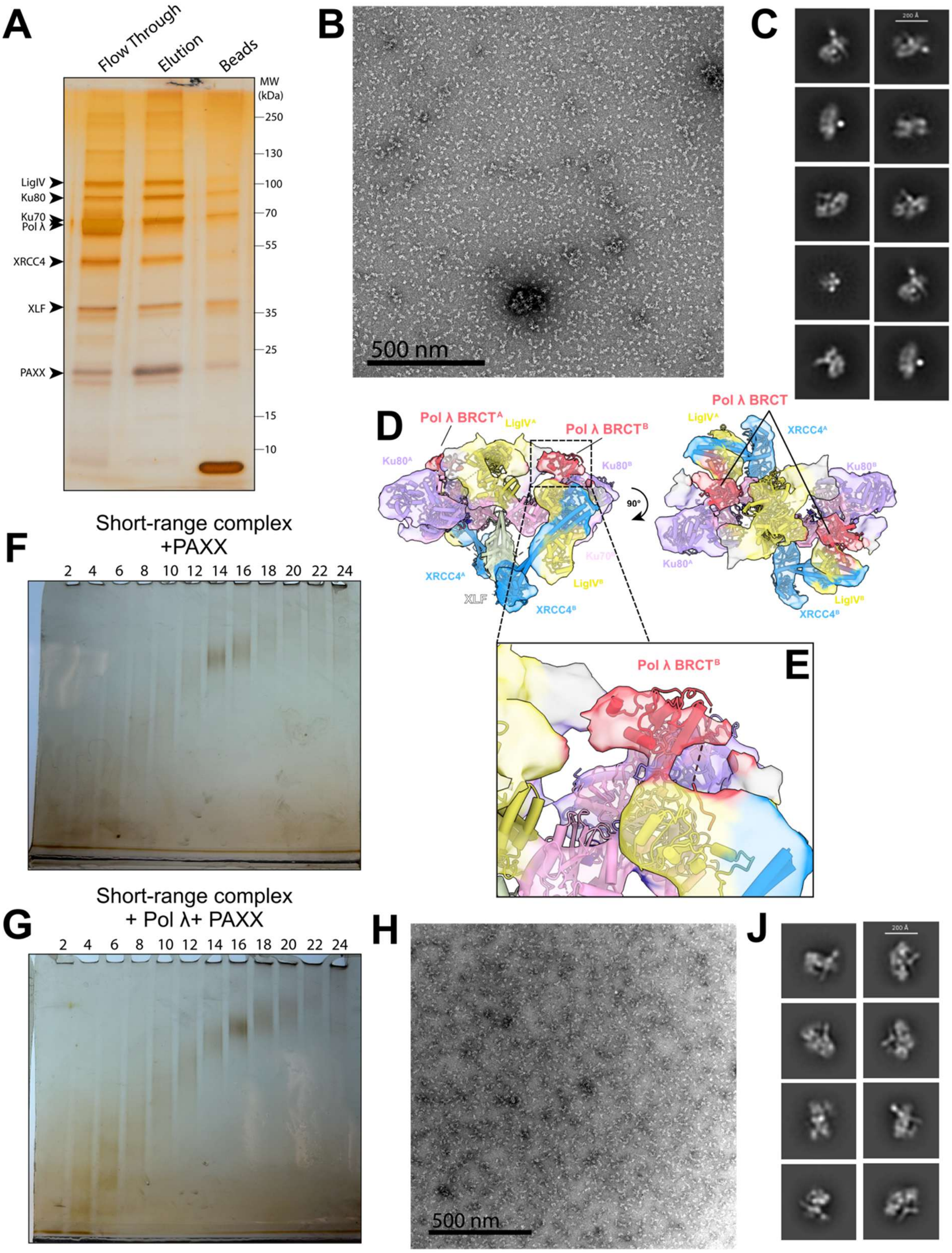
A
Flow Through
Elution
Beads
MW (kDa)
250
130
100
70
55
35
25
15
10
LigIV
Ku80
Ku70
Pol λ
XRCC4
XLF
PAXX
B
500 nm
C
200 Å
D
Pol λ BRCT^A
LigIV^A
Pol λ BRCT^B
Ku80^A
Ku80^B
90°
Pol λ BRCT
XRCC4^A
Ku70
LigIV^B
XLF
XRCC4^B
E
F
Short-range complex +PAXX
2 4 6 8 10 12 14 16 18 20 22 24
G
Short-range complex + Pol λ+ PAXX
2 4 6 8 10 12 14 16 18 20 22 24
H
500 nm
J
200 Å

Supplementary Figure 1. Assembly of SR-Pol λ complex. (A) Silver stain of a magnetic beads-based pulldown of the SR complex with Pol λ. **(B-C)** Raw micrographs and 2D class averages from NS EM of SR-Pol λ complex generated by pulldown.**(D-E)** 3D reconstruction of NS EM data from pulldown. Density corresponding to 2 copies of Pol λ BRCT domain are observed. Rigid body docking of the SR-Pol λ complex determined by cryo-EM demonstrates all components to be in the same configuration and orientation when assembled by both graFix and pulldown. **(F-G)** Non-denaturing PAGE analysis of GraFix sample preparations of the SR complex +/- Pol λ showing a clear peak shift from fractions 14-15 to 16-17.**(H-J)** Raw micrographs and 2D class averages of graFix assembled SR-Pol λ complex.

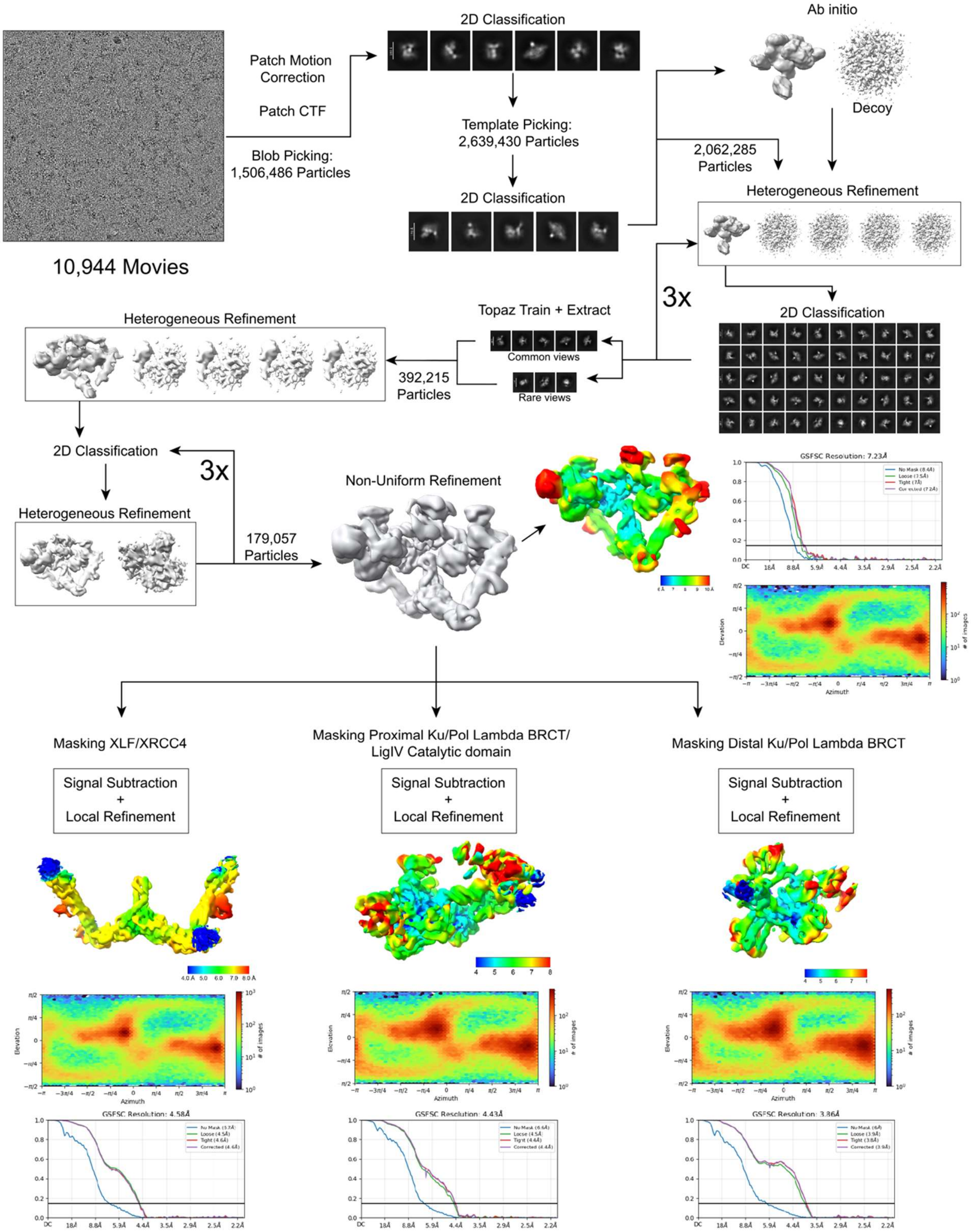

10,944 Movies
Patch Motion Correction
Patch CTF
Blob Picking: 1,506,486 Particles
2D Classification
Template Picking: 2,639,430 Particles
2D Classification
Ab initio
Decoy
2,062,285 Particles
Heterogeneous Refinement
3x
2D Classification
Topaz Train + Extract
Common views
Rare views
392,215 Particles
Heterogeneous Refinement
2D Classification
3x
Heterogeneous Refinement
179,057 Particles
Non-Uniform Refinement
GSFSC Resolution: 7.23Å
Masking XLF/XRCC4
Masking Proximal Ku/Pol Lambda BRCT/ LigIV Catalytic domain
Masking Distal Ku/Pol Lambda BRCT
Signal Subtraction + Local Refinement
Signal Subtraction + Local Refinement
Signal Subtraction + Local Refinement
GSFSC Resolution: 4.58Å
GSFSC Resolution: 4.43Å
GSFSC Resolution: 3.86Å

Supplementary Figure 2. Data-processing scheme of the SR-Pol λ complex. Flow chart of cryo-EM data processing. Gold-standard Fourier shell correlation (FSC) curves (cutoff 0.143) show the final calculated resolution of each individually refined body.

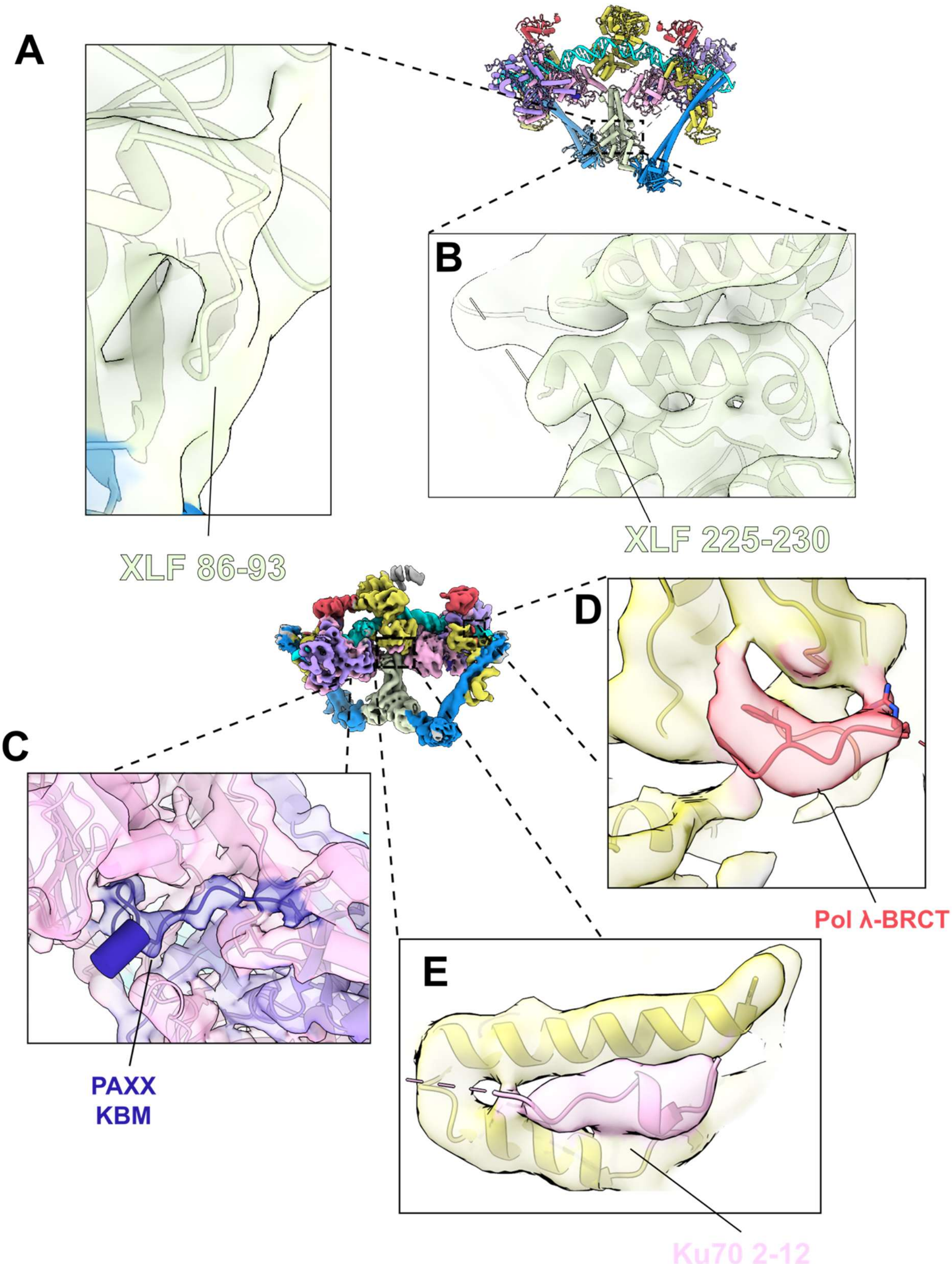

A
B
XLF 86-93
XLF 225-230
C
D
PAXX
KBM
Pol λ-BRCT
E
Ku70 2-12

Supplementary Figure 3. Structural details of NHEJ factors in SR-Pol λ complex. **(A)** Magnified view of the newly modeled loop of XLF with corresponding residues noted. **(B)** Magnified view of newly modeled extended helix of XLF with corresponding residues noted. (**C)** Magnification of the PAXX-KBM interacting with Ku70. **(D)** Magnification of cryo-EM map showing Pol lambda-NTD interaction with LigIV-BRCT1. **(E)** Magnified view of cryo-EM map showing Ku70-CTD interaction with LigIV-DBD.

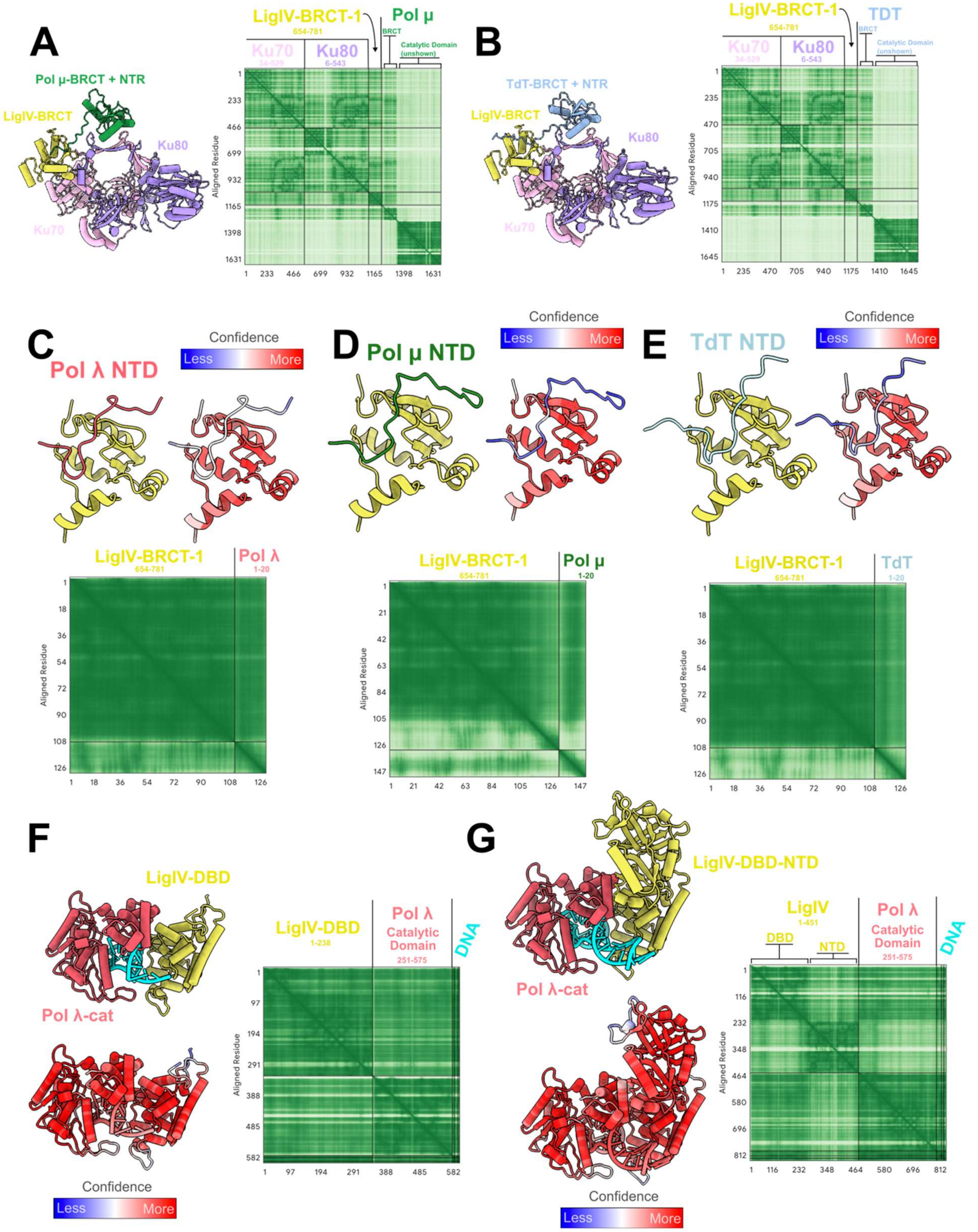
A
LigIV-BRCT-1
654-781
Pol μ
BRCT
Catalytic Domain (unshown)
Ku70
Ku80
6-543
Pol μ-BRCT + NTR
LigIV-BRCT
Ku80
Ku70
Aligned Residue
B
LigIV-BRCT-1
654-781
TDT
BRCT
Catalytic Domain (unshown)
Ku70
Ku80
6-543
TdT-BRCT + NTR
LigIV-BRCT
Ku80
Ku70
Aligned Residue
C
Pol λ NTD
Confidence
Less
More
LigIV-BRCT-1
654-781
Pol λ
1-20
Aligned Residue
D
Pol μ NTD
Confidence
Less
More
LigIV-BRCT-1
654-781
Pol μ
1-20
Aligned Residue
E
TdT NTD
Confidence
Less
More
LigIV-BRCT-1
654-781
TdT
1-20
Aligned Residue
F
LigIV-DBD
Pol λ-cat
LigIV-DBD
1-238
Pol λ
Catalytic Domain
251-575
DNA
Aligned Residue
Confidence
Less
More
G
LigIV-DBD-NTD
Pol λ-cat
LigIV
1-451
DBD
NTD
Pol λ
Catalytic Domain
251-575
DNA
Aligned Residue
Confidence
Less
More

Supplementary Figure 4. AlphaFold3 Prediction of Pol X NTDs. (A-B) PAE for Pol μ-Ku70/80-LigIV-BRCT (A) and TdT-Ku70/80-LigIV-BRCT (B). **(C-E)** AF3 prediction of first 20 residues of Pol lambda with LigIV-BRCT. Cartoons on left are colored by confidence and PAE for each is included below. **(F-G)** AF3 predictions of Pol λ catalytic domain with 1 nt gapped DNA and the LigIV DBD and LigIV DBD-NTD subdomains. Bottom cartoons are colored by confidence and PAE for each prediction is included to the left.

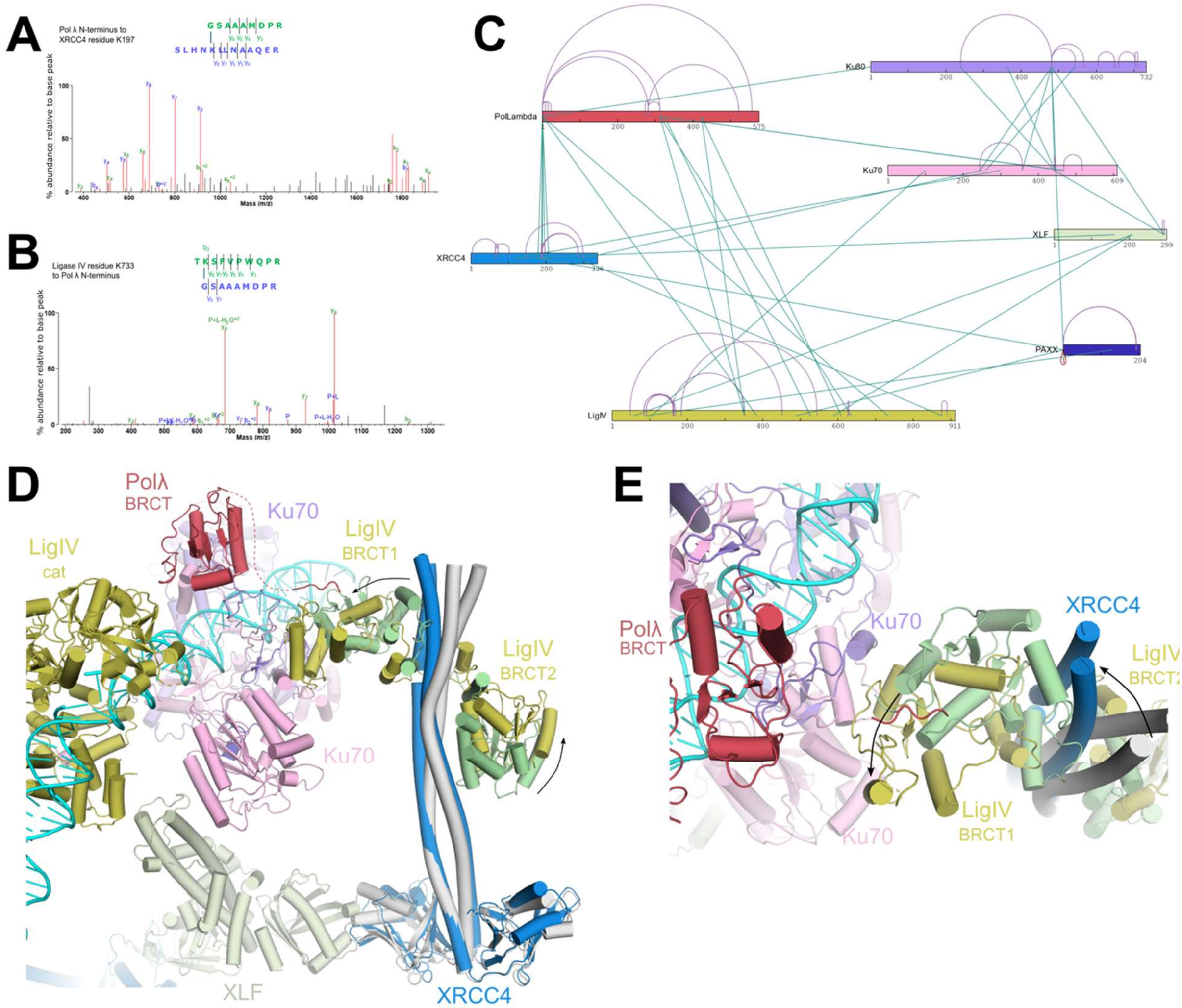

Supplementary Figure 5. XL-MS of the SR-Pol λ complex. (A-B) Representative MS/MS spectra identifying interprotein crosslinks between the Pol λ N-terminus and the XRCC4 coiled-coil (top) or the LigIV BRCT1 subdomain (bottom). Precise location of the covalent crosslink is indicated by a black line connecting the green and blue peptide sequences above each spectra. It should be noted that the Pol λ construct used in this study contains a 5-residue cloning artifact (GSAAA) that is not present in the native protein. This small addition likely extended the effective crosslinking reach from Pol λ toward the XRCC4 coiled-coil. **(C)** Diagram of all BS^3 crosslinks observed in the SR-Pol

λ complex. Intra-protein crosslinks are shown in purple and inter-protein crosslinks are shown in teal. **(D)**Cartoon diagram of the NHEJ complex, determined by CryoEM, colored as in Figure 1. The crystal structure (PDB 3II6) of the LigIV tandem BRCT domain construct (pale green) in complex with the ordered head and coiled-coil domains of XRCC4 (gray) was superimposed with the cryo-EM structure, based on residues 120-151 of the bottom of the coil. **(E)** A zoomed-in top view of the LigIV BRCT1 subdomain, its position relative to the XRCC4 coiled-coil, and interactions with the Ku heterodimer. Black arrows indicate structural differences between the cryo-EM and crystal structures.

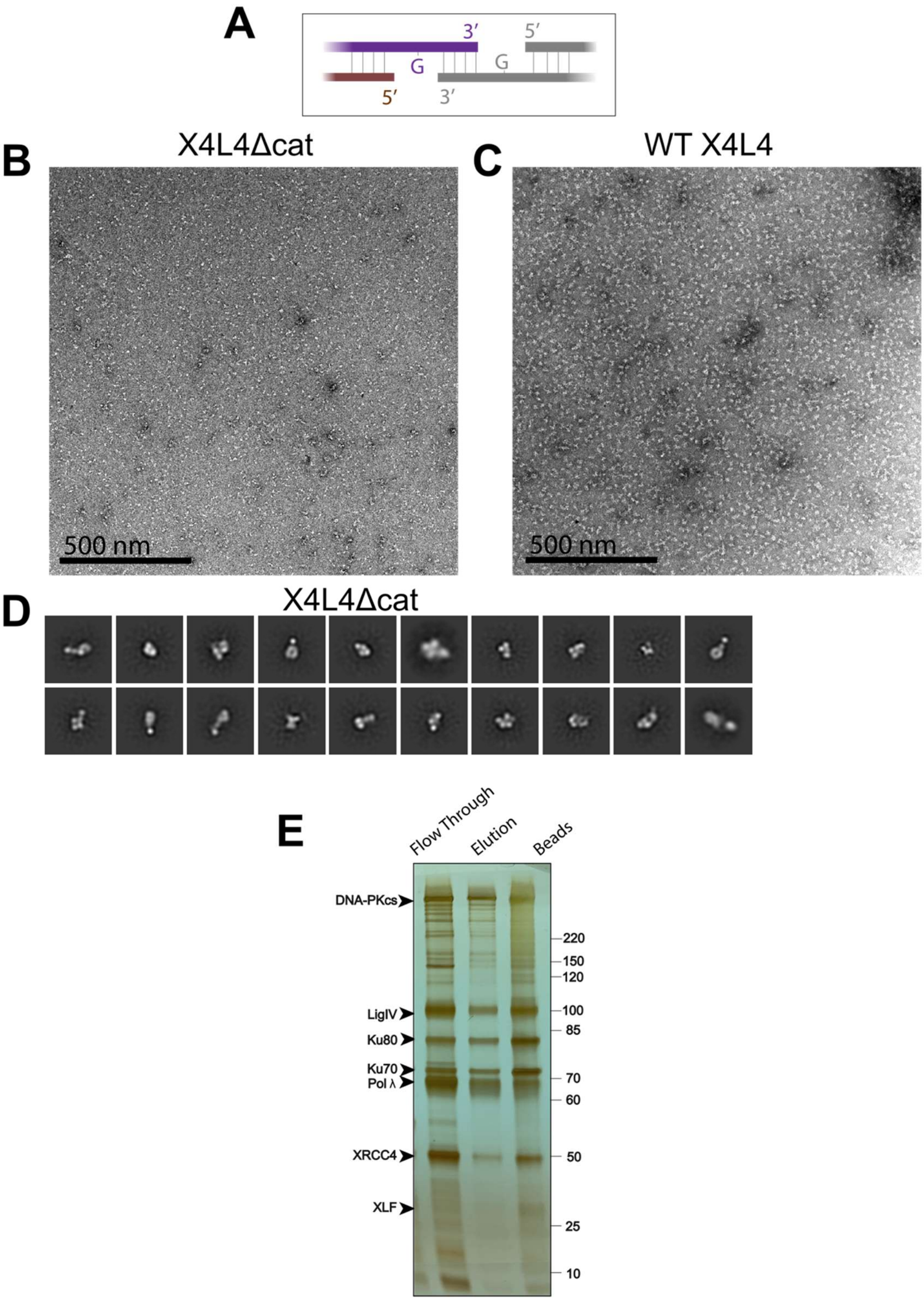
A
3′
5′
G
G
5′
3′
B
X4L4Δcat
500 nm
C
WT X4L4
500 nm
D
X4L4Δcat
E
Flow Through
Elution
Beads
DNA-PKcs
LigIV
Ku80
Ku70
Pol λ
XRCC4
XLF
220
150
120
100
85
70
60
50
25
10

Supplementary Figure 6. Negative stain of SR complex with X4L4Δcat. (A) Diagram of ends of DNA substrate used – 4 nt complementarity flanked by 1 nt gaps. **(B)** Raw micrograph of SR-Pol λ complex assembled with X4L4Δcat. Particles are noticeably smaller than those observed in **(C) -** SR complex assembled with wild-type X4L4. **(D)** Representative 2D class averages for X4L4Δcat sample. No views of a fully assembled synaptic complex are observed. **(E)** Silver stain of pulldown of LR-Pol λ complex.

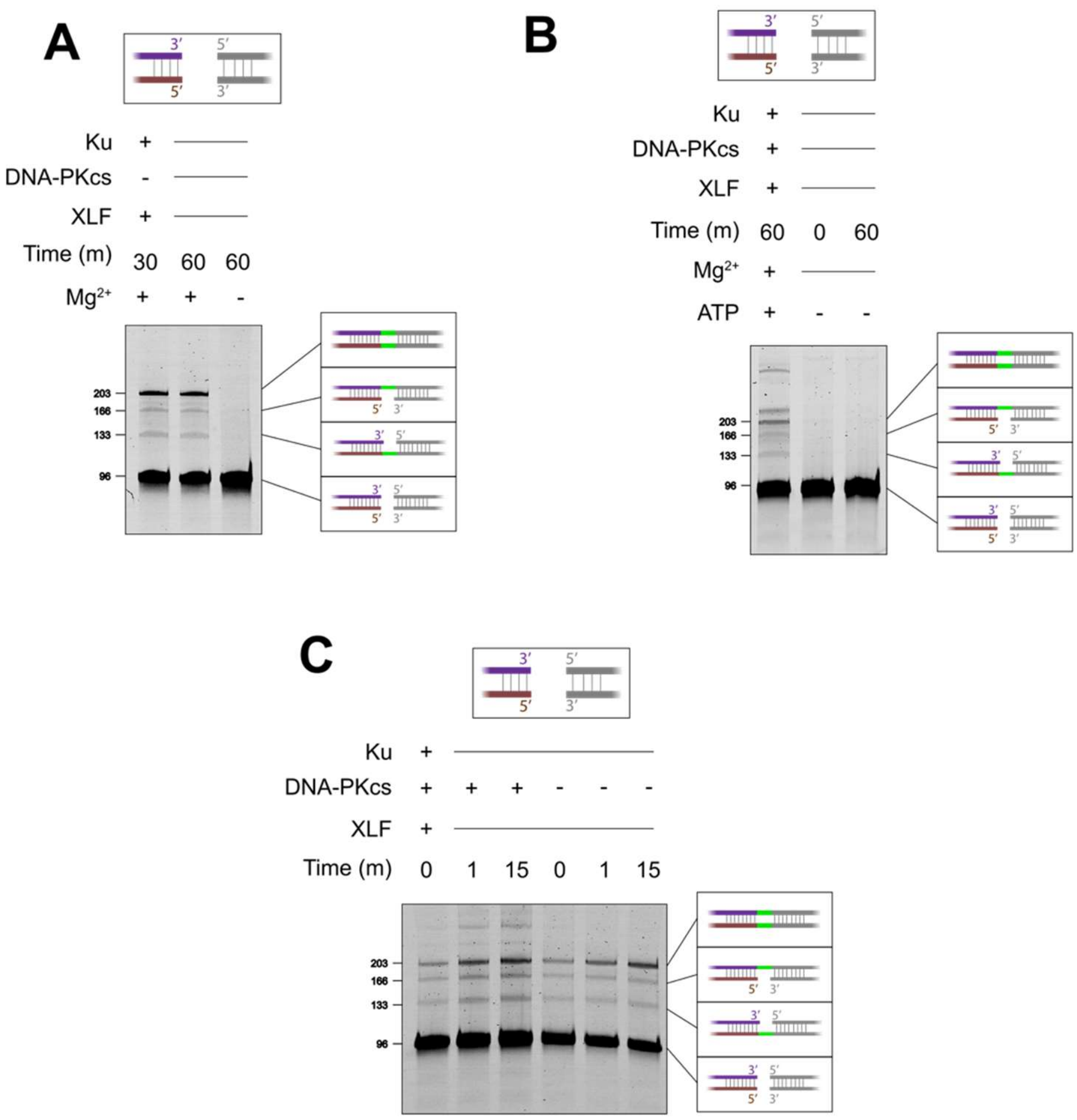

Supplementary Figure 7. Validation of activity assay on directly ligatable substrates. (A) Denaturing urea gel analysis of SR complex ligation activity demonstrating the attenuation of the reaction by addition/restriction of Mg^{2+}. **(B)** Assay of LR complex ligation. Compared to the SR complex, ligation is ATP dependent. **(C)** Time course of both LR and SR complex ligation reactions. Significant ligation is observed immediately after addition of $MgCl_2$ or ATP, indicating that repair can be carried out relatively quickly after the functional complex is assembled.

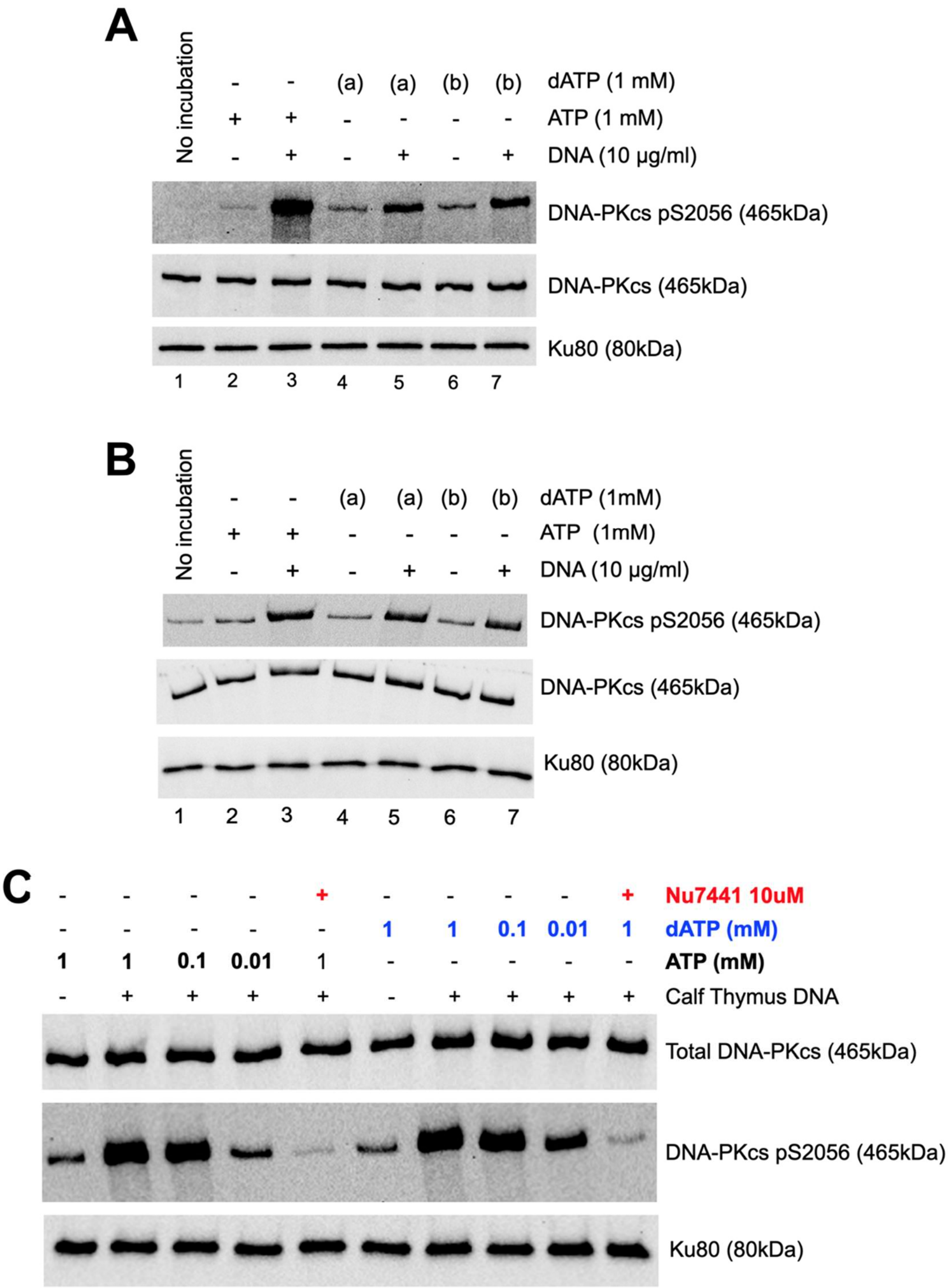
A
No incubation
dATP (1 mM)
ATP (1 mM)
DNA (10 µg/ml)
DNA-PKcs pS2056 (465kDa)
DNA-PKcs (465kDa)
Ku80 (80kDa)
1 2 3 4 5 6 7
B
No incubation
dATP (1mM)
ATP (1mM)
DNA (10 µg/ml)
DNA-PKcs pS2056 (465kDa)
DNA-PKcs (465kDa)
Ku80 (80kDa)
1 2 3 4 5 6 7
C
Nu7441 10uM
dATP (mM)
ATP (mM)
Calf Thymus DNA
Total DNA-PKcs (465kDa)
DNA-PKcs pS2056 (465kDa)
Ku80 (80kDa)

Supplementary Figure 8. (A) Purified DNA-PKcs and Ku70/80 (final concentration 0.4 μM each) in 50 mM HEPES pH 7.5, 10 mM $MgCl_2$, 0.1 mM EDTA and 0.1 mM DTT were incubated in the presence of sonicated calf thymus DNA (10 μg/ml, lanes 3,5 or 6), an equivalent volume of TE (lanes 2,4, and 6) and either 1 mM ATP (lanes 2 and 3) or dATP (lanes 4-7) at 27°C for 3 min. dATP was from (a)ThermoScientific and (b) Invitrogen, respectively. The samples in lane 1 contained DNA-PKcs and Ku only (no ATP or DNA). Reactions were stopped by addition of SDS sample buffer and samples were analysed by western blot for DNA-PKcs serine 2056 phosphorylation (Cell Signalling, cat. #68716), total DNA-PKcs (monoclonal antibody 42-27) or Ku80 (Cell Signalling, cat#2180) as shown. **(B)** Duplicate experiment confirming dATP dependent phosphorylation. **(C)** Comparison of ATP and dATP phosphorylation in the presence of DNA-PK inhibitor Nu7441.

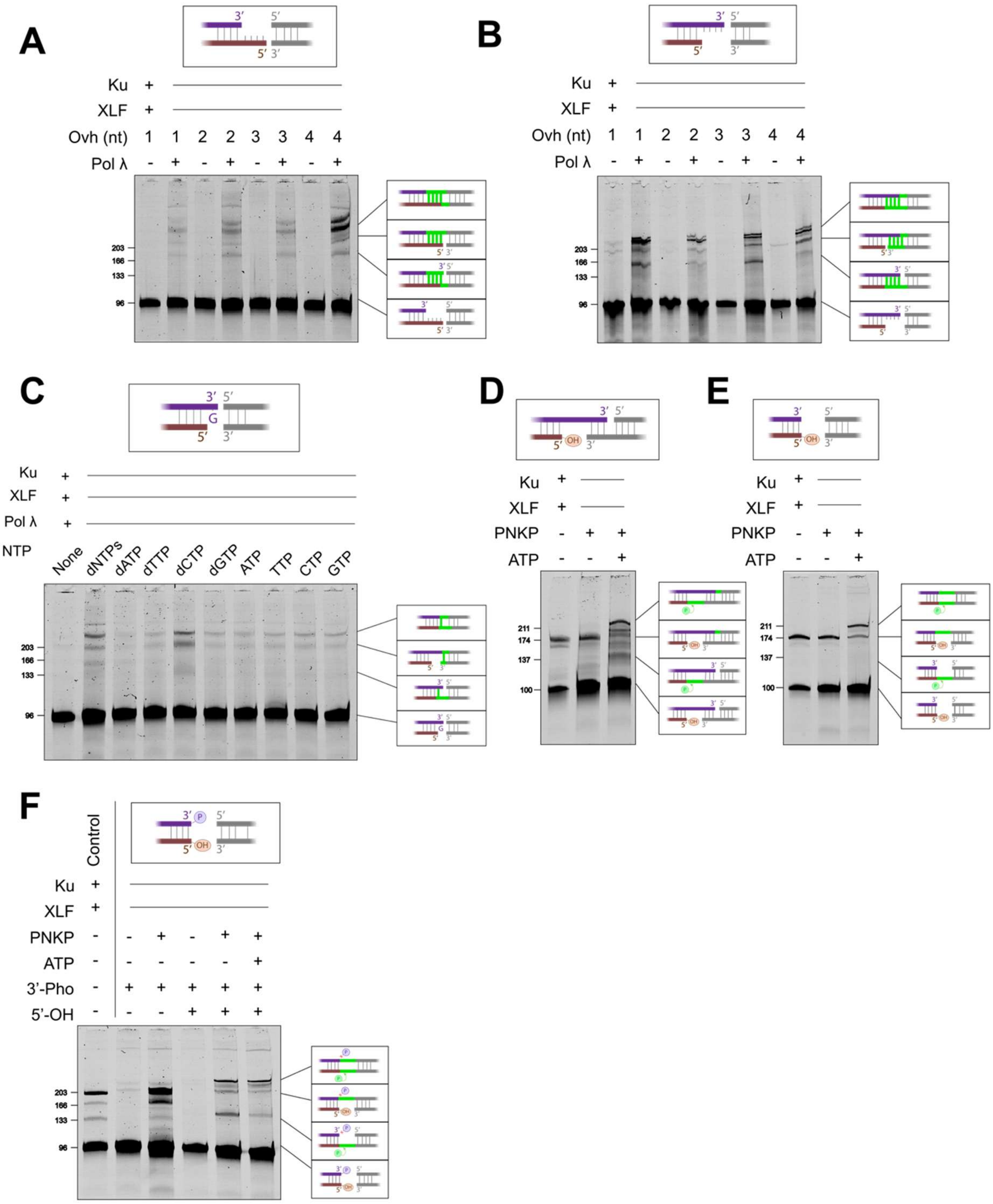
A
B
C
D
E
F
Ku
XLF
Ovh (nt)
Pol λ
NTP
None
dNTPs
dATP
dTTP
dCTP
dGTP
ATP
TTP
CTP
GTP
PNKP
ATP
Control
3'-Pho
5'-OH
203
166
133
96
211
174
137
100

Supplementary Figure 9. End-processing allows the short-range complex to effect repair on a variety of DSB configurations. (**A-B**) Pol λ dependent ligation assay of 1-4 nt 3' and 5' overhangs and blunt end DNA. **(C)** Comparison of Pol λ dependent ligation when supplied with different ribonucleotides and deoxyribonucleotides. dNTPs refers to a master mix of all dNTPs. **(D-E)** Comparison of ligation on 5'-OH substrates containing with and without microhomology. LigIV is able to carry out single ligation across from the 5'-OH in both cases. **(F)** Comparison of end-processing dependent ligation of 3'-Pho containing substrates. In contrast to those only harboring 5'-OH, the 3'-Pho appears to block functional LigIV engagement with the DSB on substrates without terminal complementarity. Control is same as in 5B

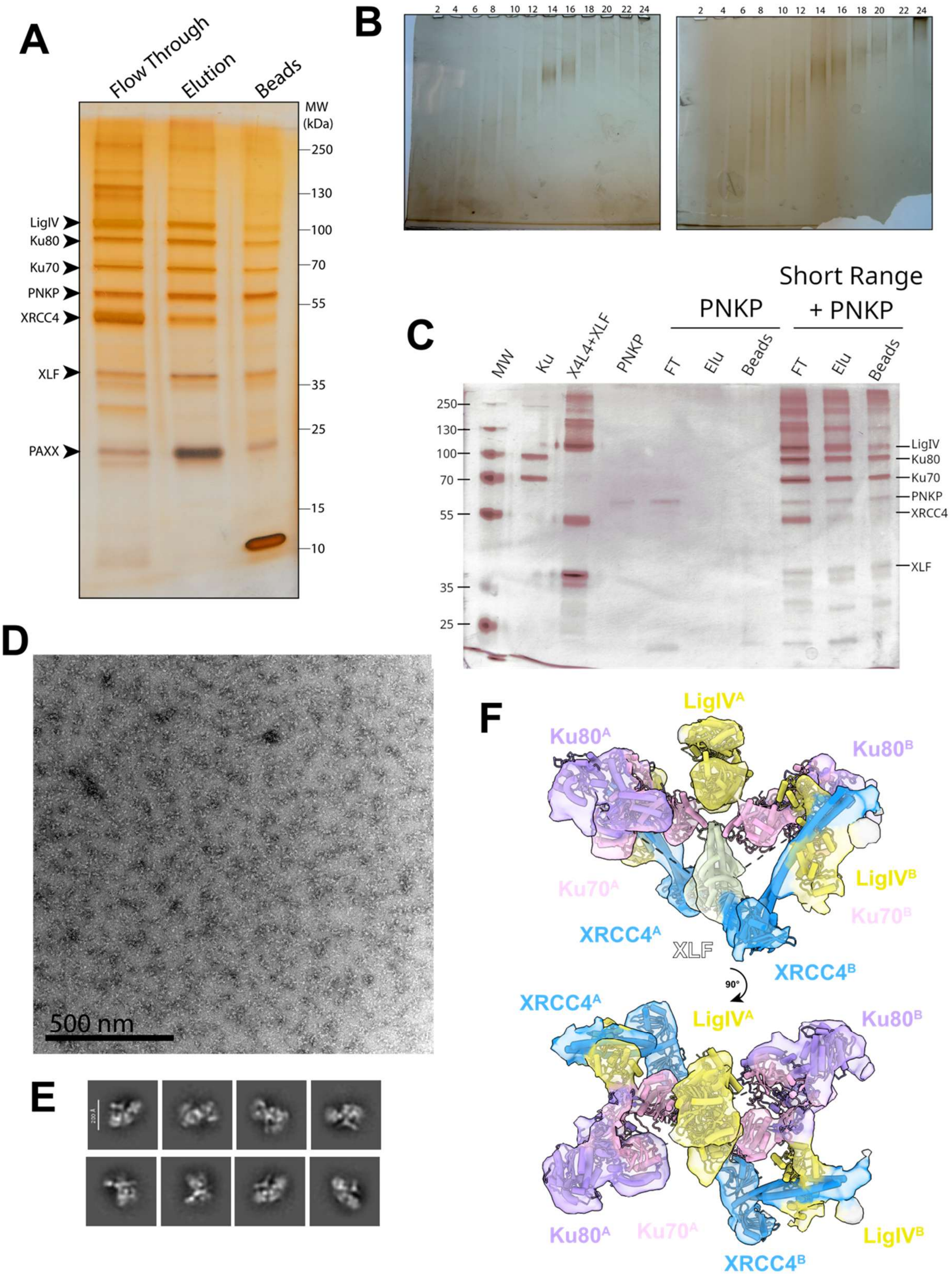
A
Flow Through
Elution
Beads
MW (kDa)
250
130
100
70
55
35
25
15
10
LigIV
Ku80
Ku70
PNKP
XRCC4
XLF
PAXX
B
2 4 6 8 10 12 14 16 18 20 22 24
C
MW
Ku
X4L4+XLF
PNKP
FT
Elu
Beads
Short Range + PNKP
250
130
100
70
55
35
25
D
500 nm
E
F
LigIV^A
Ku80^A
Ku80^B
Ku70^A
Ku70^B
LigIV^B
XRCC4^A
XRCC4^B
XLF
90°

Supplementary Figure 10. Assembly and negative stain EM of SR-PNKP complex. **(A)** Silver stain of a magnetic beads-based pulldown of the SR complex with PNKP. **(B)** Non-denaturing PAGE analysis of GraFix sample preparations of the SR complex +/- PNKP. **(C)** Pulldown of PNKP with DNA and PNKP with the other short-range complex factors (X4L4, Ku70/80, and XLF). **(D)** Negative stain EM of the SR-PNKP complex. **(E)** 2D class averages of the SR-PNKP complex from negative stain EM. **(F)** 3D reconstruction from negative stain EM of the SR-PNKP complex. No new density corresponding to PNKP is observed.

A

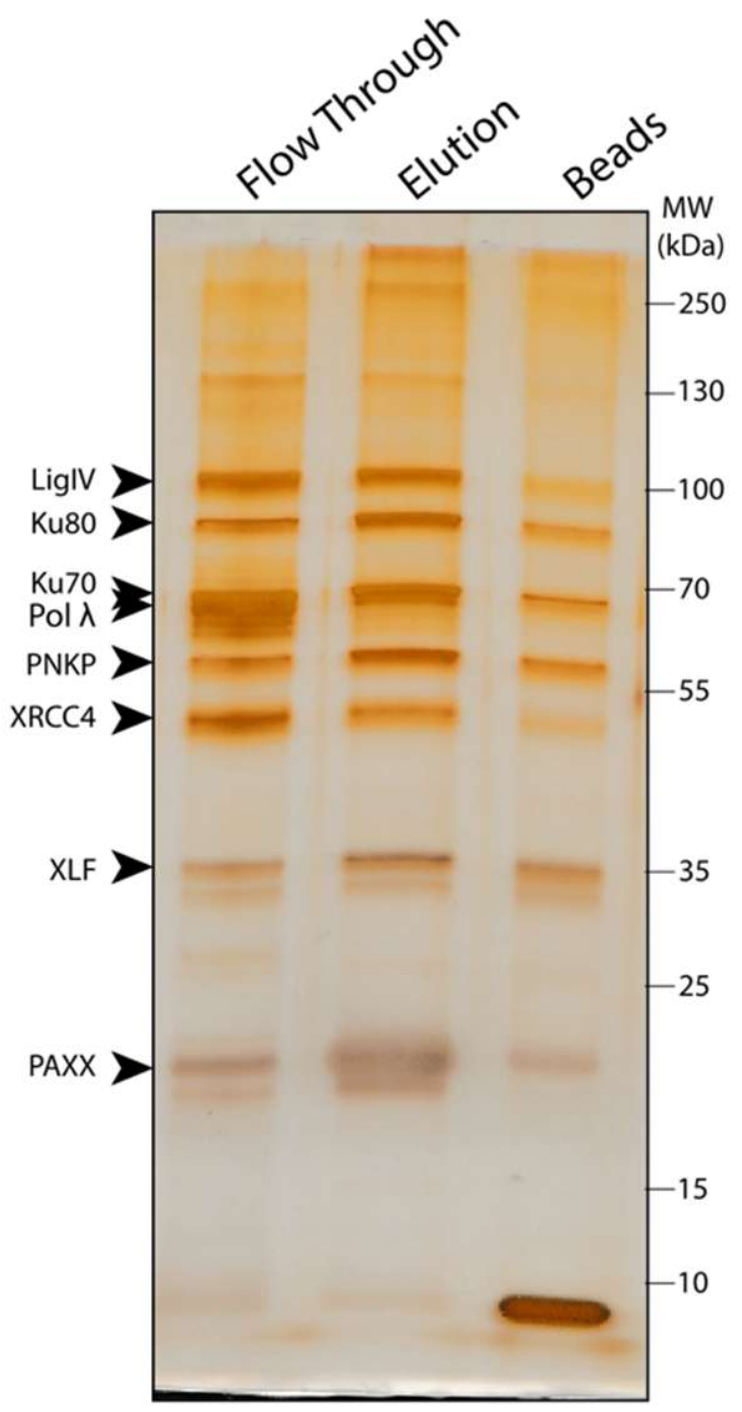
Flow Through
Elution
Beads
MW
(kDa)
250
130
100
70
55
35
25
15
10
LigIV
Ku80
Ku70
Pol λ
PNKP
XRCC4
XLF
PAXX

B

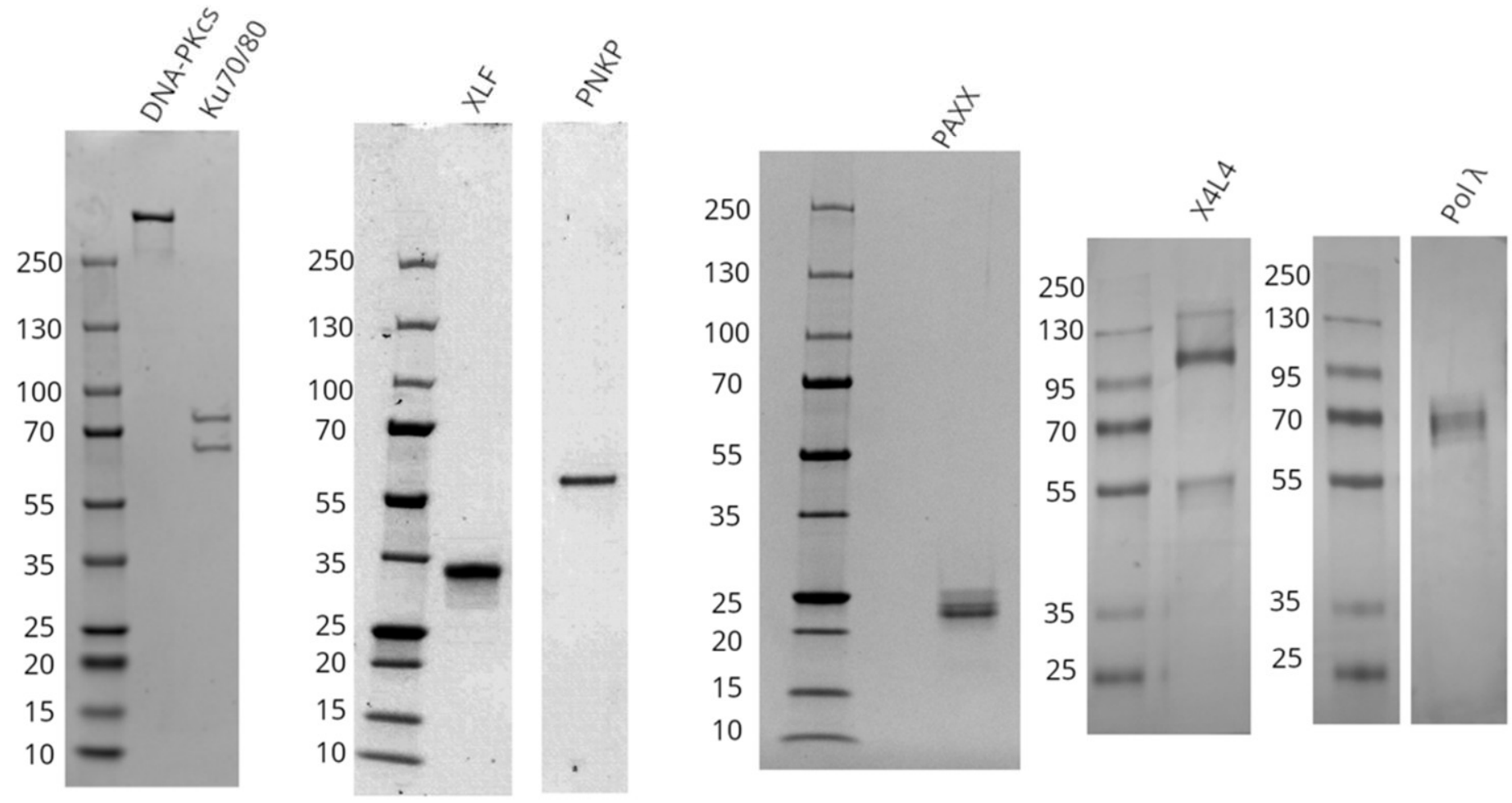
DNA-PKcs
Ku70/80
250
130
100
70
55
35
25
20
15
10
XLF
PNKP
250
130
100
70
55
35
25
20
15
10
PAXX
250
130
100
70
55
35
25
20
15
10
X4L4
250
130
95
70
55
35
25
Pol λ
250
130
95
70
55
35
25

Supplementary Figure 11. Pulldown of SR complex containing both Pol λ and PNKP. (A) Silver stain of a magnetic beads-based pulldown of the SR complex with Pol λ and PNKP. (B) Coomassie/silver stain SDS-PAGE analysis of all purified proteins used in experiments.

Supplementary Table 1. Cryo-EM data collection, refinement and validation statistics

	#1 Short-range Pol λ complex (EMDB 70958, 70957, 70959, 70949) (PDB 9OX8)
**Data collection and processing**	
Magnification	30k
Voltage (kV)	300
Electron exposure (e–/Å^2)	
Defocus range (μm)	-1.5 to -4.0
Pixel size (Å)	1.056
Symmetry imposed	C1
Initial particle images (no.)	1,506,486
Final particle images (no.)	179,057
Map resolution (Å) FSC threshold = 0.143	7.23 (overall), 4.58 (XRCC4-LigIV, XLF scaffold), 4.43 (proximal Ku, Pol λ BRCT, LigIV catalytic domain), 3.86 (distal Ku, Pol λ BRCT)
Map resolution range (Å)	3.86 to >10
**Refinement**	
Initial model used (PDB code)	7LSY
Model resolution (Å) FSC threshold	4.58 (XRCC4-LigIV, XLF scaffold), 4.43 (proximal Ku, Pol λ BRCT, LigIV catalytic domain), 3.86 (distal Ku, Pol λ BRCT) 0.143
Model resolution range (Å)	n/a
Map sharpening *B* factor (Å^2)	-615 (overall), -169 (XRCC4-LigIV, XLF scaffold), -147 (proximal Ku, Pol λ BRCT, LigIV catalytic domain), -123 (distal Ku, Pol λ BRCT)
Model composition	
Non-hydrogen atoms	77,946
Protein residues	4,632 (Nucleotide: 136)
Ligands	0
B factors (Å^2)	Not estimated
Protein	
Ligand	
R.m.s. deviations	
Bond lengths (Å)	0.013
Bond angles (°)	1.614
Validation	
MolProbity score	2.20
Clashscore	13.25
Poor rotamers (%)	1.42
Ramachandran plot	
Favored (%)	92.96
Allowed (%)	7.04
Disallowed (%)	0.00
