# Supplementary material for: End processing in NHEJ by Polymerase λ and PNKP is coordinated during short-range synapsis
Source: Nat Commun. 2026 Sep 9;17:10645. doi: 10.1038/s41467-026-77503-2 (PMC13649775; doi:10.1038/s41467-026-77503-2)
Supplement: Supplementary file 2 [file 41467_2026_77503_MOESM2_ESM.pdf]

Description of Additional Supplementary Files

Filename: Supplementary Data 1

Description: List of DNA constructs used in this study.
