# Supplementary material for: End processing in NHEJ by Polymerase λ and PNKP is coordinated during short-range synapsis
Source: Nat Commun. 2026 Sep 9;17:10645. doi: 10.1038/s41467-026-77503-2 (PMC13649775; doi:10.1038/s41467-026-77503-2)
Supplement: Supplementary file 4 [file 41467_2026_77503_MOESM5_ESM.pdf]

nature portfolio

Peer Review File

End Processing in NHEJ by Polymerase λ and PNKP is coordinated during short-range synapsis

Corresponding Author: Professor Yuan He

This file contains all reviewer reports in order by version, followed by all author rebuttals in order by version.

Version 0:

Reviewer comments:

Reviewer #1

(Remarks to the Author)
Vogt et al. investigate the coupling between end-processing by polymerase λ (Pol λ) and end synapsis during non-homologous end joining (NHEJ) by solving the cryo-EM structure of a core NHEJ complex comprising Ku70/80, XRCC4–LigIV, XLF, PAXX, and Pol λ. The study also attempts to explore the interplay between PNKP-mediated end-processing and short-range synapsis through biochemical assays using 3'P/5'OH DNA ends. Based on their in vitro ligation experiments, the authors propose a coordinated model involving both Pol λ and PNKP acting during the short-range synaptic stage.

While the structural work adds value, particularly by placing Pol λ in the NHEJ synaptic complex, the conceptual framework of coordinated end processing is not novel. The idea that multiple end-processing factors act in a coordinated manner within the short-range complex has already been proposed (e.g., PMID: 31862156), based on their coexistence in nuclear extracts. Furthermore, several key conclusions in this manuscript rely heavily on speculative interpretation rather than direct evidence (see points 5, 7, and 12 below).

In summary, although the structural characterization of the Pol λ-containing complex is potentially of interest, the resolution is relatively low and does not surpass that of previous structural work involving other X-family polymerases (e.g., Pol μ; PMID: 40500445). Taken together, the novelty and mechanistic depth of this work may not fully meet the publication standards of Nature Communications.

Specific Comments:

1. The manuscript should provide detailed information on the purity and potential nuclease contamination of the recombinant NHEJ proteins used. Inclusion of SDS-PAGE or silver-stained gels for all purified components is recommended.

2. Western blot analysis should be performed to confirm the presence of all claimed NHEJ proteins, especially Pol λ, in the pull-downs shown in Fig. S1A and S8.

3. The authors should quantify the stoichiometry of each NHEJ factor in both the pull-down and cryo-EM complexes. The relative abundance of proteins may differ between biochemical and structural assays. Additionally, the rationale for including PAXX in all buffer conditions (reaction, wash, and elution) should be clearly stated.

4. Could the authors clarify why the Cy5-labeled half was pulled down by the unlabeled half in the presence of X4L4 alone, as shown in Fig. 4B (lane 1)? Regardless of the ligation efficiency, a greater amount of Cy5-labeled DNA was pulled down in the presence of X4L4 and XLF (lane 3) compared to X4L4 alone (lane 1). Does this mean X4L4+XLF can mediate association between the two DNA ends? Similar concerns apply to other pull-down–coupled ligation assays throughout the manuscript. If synapsis efficiency varies, differences in ligation efficiency would be expected as a consequence.

5. The claim that Pol λ is stably recruited to the LR complex (line 238) is primarily based on its enzymatic activity. However, since Pol λ can promote synapsis and gap filling independently (PMID: 35778389), this does not confirm stable recruitment. A gel shift assay with Pol λ alone is needed to substantiate this point.

6. In lines 88–89, the authors suggest PAXX alone cannot support ligation. However, previous studies (e.g., PMID:

27705800; 31399561) indicate that PAXX can stimulate ligation in the absence of XLF. This should be addressed.

7. While AlphaFold3 predictions are used to model potential interaction interfaces between NHEJ proteins, experimental validation is lacking. Mutagenesis or truncation analyses are essential to support these computational models.

8. In lines 220–234, the authors claim that the Ku + X4L4 complex is unable to mediate gap-crossing ligation, and that Pol λ is required to fill the gap prior to ligation. However, as shown in the last two lanes of Fig. 4E, the presence of either dATP or dCTP leads to detectable gap-crossing ligation products, albeit at lower levels than the nick-ligation product. Similarly, gap-crossing ligation products are evident in lanes 2 and 7 of Fig. 4F. These results suggest that a certain degree of gap-crossing ligation may occur independently of Pol λ activity. To substantiate the role of Pol λ in gap filling, the authors should provide sequencing data of the ligation junctions.

9. The assertion (lines 243–248) that DNA-PKcs can use dATP as a phosphorylation cofactor lacks direct supporting evidence. Experimental confirmation is necessary.

10. The claim that PNKP forms a stable complex with core NHEJ factors (lines 271–272) is not supported by a clear shift or enrichment in the gel shown in Fig. S7D. This should be validated with further data.

11. The authors should explain why ATP appears to inhibit 5' OH phosphorylation, as suggested by reduced ligation in the final lane.

12. The conclusion that PNKP activity occurs specifically within the short-range synaptic complex is not adequately supported. Observing specific ligation in PNKP-containing reactions does not rule out activity prior to or outside of the SR synapsis. Additional temporal or structural evidence is needed.

Reviewer #2

(Remarks to the Author)
This manuscript by Vogt et al seeks to understand the mechanism by which end-processing occurs within the SR complex during NHEJ. To this end, they solved a CryoEM structure of the SR complex in which the processing factor Pol λ is bound. In the structure, LigIV adopts an 'open' conformation, which the authors speculate would allow end-processing by accessory NHEJ factors. In addition, the authors show that they can preassemble NHEJ complexes containing both PNKP and Pol λ that carry out end joining reactions that require the activity of both processing factors. Overall, the work is a rather modest step forward in our understand of NHEJ end processing given recent published structures by the Chaplin (Frit Nature Communications 2025) and Yang labs (Liu Nature 2025). Given this and other major concerns listed below, I do not believe the work merits further consideration at Nature Communications.

Major Concerns:

1.) In the abstract (lines 32-33) the authors assert they have 'uncovered the mode of Pol λ recruitment to the complex'. This is a bold claim given prior work in the literature including (Frit Nat Comm 2025; Lee JBC 2004; Fan Biochem and Biophys Res Comm 2004; McElhinny Mol Cell 2005).

2.) The newly presented CryoEM structures show no evidence of Pol λ or PNKP engagement with the DNA, only with other proteins in the SR complex. In addition, all contacts between proteins were previously known and this is simply a composition of previously found contacts. The newly found open conformation is intriguing from an end-processing point of view, but no experiments were performed to validate the existence of the state or the dependence of end-processing within the SR complex on this open state. It's possible this state may simply represent an intermediate in the removal of LigIV from the SRC to allow processing, as presented in Wei Yang's recent work (Nature, June 2025).

3.) A substantial number of figure panels is Alphafold modeling (Figures 2D, 2E, 3B, 3E, 5A) yet none of these novel interactions have been confirmed with any mutational analysis in vivo or in vitro. For example, if the Pol λ contacts between Pol λ and LigIV are important for SR complex formation / recruitment / processing, mutation of Pol λ in that area should abolish activity. As acknowledged by the authors the proposed interaction between TdT/Pol μ and LigIV seems quite tenuous (lines 141-142; Figure 2G/H) and most likely is an artifact by Alphafold. Including PAE maps would also help with their analysis of these potential interactions. Doing a simple pull-down assay between TdT/LigIV would also help to validate this proposed interaction.

5.) There are several issues with the in vitro assays performed for this work:
1.) The authors have also not quantified any of the joining making it difficult to compare the activity between their assays and other previously published work.
2.) Gel loading of nearly all presented gels is uneven and the banding pattern suggests a form of degradation or lack of homogeneity in sample preparation.
3.) The authors make conclusions about the timing of events for joining on the top strand vs the bottom strand without doing a time course. A time course is critically important and essential to draw these types of conclusions.
4.) The banding pattern in most gels (Figures 4C, 4D, 4F, 5B, 5D, 5E, 6A, 6B, 6C, 6D) shows a doublet at the fully joined species without any given explanation. Sequencing of these products would vastly help the interpretation of the data. In addition, it looks like some of the products are mislabeled. For example, in Figure 6A, it appears the singly ligated product is

the band shown in lane 3, not the small and diffuse band as labeled; again, sequencing of the products would resolve these issues.

6.) Figures 4 and 5 provide little to no new information on the activities of Pol λ and PNKP, respectively. These figures are useful in confirming the activity of the proteins used in the study, though the data seems more appropriate for supplemental information than main text figures given the previous characterization of their activities.

Reviewer #3

(Remarks to the Author)
The authors reported a short-range synapse cryo-EM complex that contains Polλ-BRCT, Ku70/80, LigIV, XRCCC4, XPF, and PAXX. They observed interesting conformations showing that LigIV-DBD adopts an open conformation and Poλ-BRCT-NTR interacts with the Ku70/80 and LigIV-BRCT domains. They carried out a series of biochemical experiments to show that polλ facilitates repair of gap and overhang substrates even in the presence of DNAPKcs, while PNKP facilitates repair of 3'-Pho and 5'-OH moieties. The combination of both Polλ and PNKP in the SR or LR complex facilitates repair of both lesions. Overall, the experiments are logically executed, the manuscript is well written, and the results provide a wealth of architectural and structural data with which prior and ongoing experiments should be reconciled.

Before publication the authors should consider the following points.

Points.

1. There is an apparent disconnection between the new structure and biochemical experiments. While structure explains how Polλ-BRCT-NTR binds the SR complex, it does not generate a hypothesis to test in the biochemical experiments. For example, Polλ-BRCT has been shown to interact with Ku-70/80 in a recent cryo-EM structure. Authors also point out the BRCT domains between polλ, polµ, and TdT bind at nearly identical sites. The difference of these polymerases could come from the difference in their NTR, for which the authors here identified a new NTR interaction with LigIV-BRCT. Could this interaction be tested and shown that the interface between Polλ-NTR and LigIV-BRCT is critical for repair? If feasible, a comparison with Polµ and TdT would be a strong addition to the manuscript.

2. Although the in vitro ligation system the authors generated has the advantage of obtaining a stable and homogenous NHEJ complex, it may not be able to capture a transient interaction component, especially if the mutation/deletion study is performed. Were each of the biochemical studies supported by the PAGE gel? If so, these should be included. If not the limitations should be noted.

3. There is a lot of interesting new structural information and analysis, which likely informs prior studies and future efforts that merits more discussion. In this regard, the authors should clarify where their results support or extend the recent cryo-EM structure of Polλ-BRCT with Ku and LigIV published this year (https://www.nature.com/articles/s41467-025-59133-2). Are these results consistent with the kinetic mechanism and evident role of lnRNA in the ligation complex shown by single-molecule kinetics (Wang JL, et al.. Dissection of DNA double-strand-break repair using novel single-molecule forceps. Nat Struct Mol Biol. 2018 Jun;25(6):482-487. doi: 10.1038/s41594-018-0065-1; Thapar et al., Mechanism of efficient double-strand break repair by a long non-coding RNA. Nucleic Acids Res. 2020 48:10953-10972. doi: 10.1093/nar/gkaa784)? How may these results change or support the analyses of DNA-PKcs sequence conservation (Lees-Miller et al., Uncovering DNA-PKcs ancient phylogeny, unique sequence motifs and insights for human disease. Prog Biophys Mol Biol. 2021 163:87-108. doi: 10.1016/j.pbiomolbio.2020.09.010), XLF regulation of the XRCC4· ligase IV complex (Hammel et al., XLF regulates filament architecture of the XRCC4·ligase IV complex. Structure. 2010;18:1431-42. doi: 10.1016/j.str.2010.09.009), and the authors' prior review (Williams et al., Structural insights into NHEJ: building up an integrated picture of the dynamic DSB repair super complex, one component and interaction at a time. DNA Repair (Amst). 2014 17:110-20. doi: 10.1016/j.dnarep.2014.02.009)?

Version 1:

Reviewer comments:

Reviewer #1

(Remarks to the Author)
The authors have made substantial efforts to revise the manuscript and have addressed most of my previous concerns. However, two issues remain:

1. For Fig. 1 in the rebuttal letter, the authors should provide the full gel image with the bottom portion clearly visible. This is important because, in some cases (e.g., with Ku and PNKP), the intact substrate band appears fainter than in the control when proteins are included in the reaction. This raises the possibility that shorter products may be present at the bottom of the gel or may have run off the gel.
To address this concern, the authors should quantify the band intensities (e.g., grayscale analysis) to determine whether there is any reduction in the total input substrate. While this issue may not critically affect the overall conclusions, providing quantitative analysis would strengthen the interpretation and ensure that differences between conditions are not due to

unequal substrate loading or loss.
Additionally, the authors state in several places in the rebuttal letter that quantification is not important. However, when comparing different experimental conditions, quantitative data provide clearer and more reliable support for conclusions. Without such analysis, it remains difficult to exclude the possibility that variations arise from differences in input material. Nevertheless, as noted by the authors, this issue may not significantly alter the main conclusions in this manuscript.

2. In point 8, the authors state that sequencing of repair junctions is technically challenging due to the absence of suitable primer sites. However, this limitation can be overcome by amplifying the repair products, followed by TA cloning into a plasmid vector. The cloned fragments can then be propagated in E. coli and subjected to Sanger sequencing.
Regarding the authors' claim (lines 297–304) that Pol λ incorporates mismatched nucleotides leading to double-ligated products, this conclusion is not fully supported in the absence of junction sequence data. An alternative explanation—such as gap-crossing ligation—could also account for the observed products. Therefore, the authors should at least acknowledge this possibility and revise their wording accordingly to avoid overinterpretation.

Reviewer #2

(Remarks to the Author)
The revision of the manuscript by Vogt et al is improved by changing the structure of the figures as well as the addition of XL-MS data. The authors were able to address most of my major concerns. The result is a much more rigorous manuscript albeit still relatively modest scientific advance.

Reviewer #3

(Remarks to the Author)
The revised manuscript by Vogt et al. presents a compelling structural and biochemical investigation into the coordination of Pol λ and PNKP within the NHEJ supercomplex. By determining the cryo-EM structure of Pol λ bound to the SR synaptic complex and complementing this with XL-MS and biochemical assays, the authors provide a key snapshot of how accessory factors access DSBs without compromising the integrity of the core repair scaffold. In my view, the authors have provided a suitable rebuttal, adding significant experimental data that successfully addresses previous critiques.
A notable result is the structural elucidation of Pol λ's recruitment. While the BRCT domain interaction with Ku70/80 was anticipated, identification of a novel interaction between the Pol λ NTR and the LigIV-BRCT1 domain is a significant advance. Capturing the LigIV catalytic domain in an "open" conformation on incompatible DNA ends provides a vital structural intermediate. This nicely explains how the DNA ends become physically accessible to processing polymerases while the complex remains tethered by the LigIV DNA-binding domain (DBD). The results successfully bridge structural modeling with functional readouts. The use of a pulldown-based ligation assay with specifically designed chemically blocked or gapped substrates convincingly shows the sequential, coordinated action of Pol λ and PNKP within the intact SR complex. This data is broadly significant for DNA repair and structural biology. It reveals the fundamental dynamic nature of NHEJ and complements established literature. The authors improved their analyses to synergize with contemporaneously published works (e.g., Frit et al., 2025; Liu et al., 2025), positioning their SR-Pol λ structure as a complementary, distinct step in the repair process.
The authors' experimental revisions are exemplary. The original manuscript relied heavily on AlphaFold3 predictions for novel interfaces (such as the Pol NTR-LigIV interaction). The authors have responded to reviews by doing XL-MS, which provided orthogonal, empirical evidence for these contacts. Furthermore, they improved comments regarding gap-crossing ligation and PAXX redundancy, aligning their statements with their in vitro system data. They provided clear scientifically sound justifications for technical limitations (e.g., why direct sequencing of the hairpin-containing ligation products was not feasible).The new data (XL-MS, fluorescence polarization, negative stain EM of pull-downs, and DNA-PKcs phosphorylation assays) nicely support the improved conclusions. By explicitly differentiating between cryo-EM-derived density and AlphaFold3-assisted modeling (including visual blurring of predicted regions in the figures), the authors ensure the evidence directly supports their structural analyses. The overall global resolution of the cryo-EM map (7.23 Å) is modest but expected for an asymmetric, multi-megadalton complex with highly flexible accessory domains. The use of localized refinement (achieving ~3.8–4.5 Å) completely justifies their rigid-body docking and modeling. Their interpretation of the in vitro biochemical data is appropriately measured and avoids over-quantification of inherently variable pulldown assays. The authors also rightly note that their pulldown system captures stable endpoints.
The combination of state-of-the-art structural biology (GraFix, local refinement, XL-MS) with tailored biochemical assays yields a high-quality, comprehensive advance. The methodology is robust and adheres to modern structural biology standards, including proper validation metrics for cryo-EM (Table 2) and mass spectrometry. The methods section details protein expression/purification, complex assembly, GraFix stabilization, and single-particle processing pipelines with sufficient granularity for reproduction. The mechanistic insights into how cancer-relevant DNA repair pathways maintain fidelity will appeal to a broad audience of biochemists, structural biologists, and oncologists. No further experimental evidence is reasonably required for publication.

Open Access This Peer Review File is licensed under a Creative Commons Attribution 4.0 International License, which permits use, sharing, adaptation, distribution and reproduction in any medium or format, as long as you give appropriate credit to the original author(s) and the source, provide a link to the Creative Commons license, and indicate if changes were made.
In cases where reviewers are anonymous, credit should be given to 'Anonymous Referee' and the source.
The images or other third party material in this Peer Review File are included in the article's Creative Commons license, unless indicated otherwise in a credit line to the material. If material is not included in the article's Creative Commons license and your intended use is not permitted by statutory regulation or exceeds the permitted use, you will need to obtain permission directly from the copyright holder.
To view a copy of this license, visit https://creativecommons.org/licenses/by/4.0/

Authors' Response

We thank all reviewers for their thorough reading of our manuscript and detailed feedback. In response to their comments we have performed 9 new experiments in addition to new analysis of our data which we include in Figures 2F, 3C, 3G, Supplemental Figures S1, S4, S5, S6E, S8, S10, and a new supplementary data file.

We have also made substantial changes to the body of the manuscript to incorporate reviewers' suggestions as well as incorporate findings published contemporaneously with our submission. Manuscript line numbers are referenced throughout the responses below. Please find 5 reviewer specific figures included below to further clarify points of our rebuttal.

Figure	Changes
Figure 1	No change
Figure 2	2E – has been changed so that so that the catalytic domains of Pol λ are blurry to indicate motion.<br>2F – new experimental data from XL-MS
Figure 3	3C – new experimental data from fluorescence polarization<br>3G – new experimental data from XL-MS
Figure 4	Consolidated from Figs. 4 and 5 from our initial submission
Figure 5	No change
Figure S1	S1D-F – new experimental data from negative stain EM
Figure S2	No Change
Figure S3	S3C-E – new panels highlighting cryo-EM density
Figure S4	New supplementary figure - AlphaFold3 confidence metrics and new analysis
Figure S5	New supplementary figure - XL-MS data and supporting structural analysis
Figure S6	S6E – new silver stain of LR Pol λ complex
Figure S7	No change
Figure S8	New supplementary figure – data from DNA-PKcs dATP phosphorylation experiments
Figure S9	New supplementary figure comprising NHEJ Pol λ and PNKP functional assays on various substrates.
Figure S10	S10C – new pulldown experiment
S11	S11B – new panel of SDS-PAGE of all purified factors.

REVIEWER COMMENTS

Reviewer #1 (Remarks to the Author):

Vogt et al. investigate the coupling between end-processing by polymerase λ (Pol λ) and end synapsis during non-homologous end joining (NHEJ) by solving the cryo-EM structure of a core NHEJ complex comprising Ku70/80, XRCC4–LigIV, XLF, PAXX, and Pol λ. The study also attempts to explore the interplay between PNKP-mediated end-processing and short-range synapsis through biochemical assays using 3'P/5'OH DNA ends. Based on their in vitro ligation experiments, the authors propose a coordinated model involving both Pol λ and PNKP acting during the short-range synaptic stage.

While the structural work adds value, particularly by placing Pol λ in the NHEJ synaptic complex, the conceptual framework of coordinated end processing is not novel. The idea that multiple end-processing factors act in a coordinated manner within the short-range complex has already been proposed (e.g., PMID: 31862156), based on their coexistence in nuclear extracts. Furthermore, several key conclusions in this manuscript rely heavily on speculative interpretation rather than direct evidence (see points 5, 7, and 12 below).

In summary, although the structural characterization of the Pol λ-containing complex is potentially of interest, the resolution is relatively low and does not surpass that of previous structural work involving other X-family polymerases (e.g., Pol μ; PMID: 40500445). Taken together, the novelty and mechanistic depth of this work may not fully meet the publication standards of Nature Communications.

We thank the reviewer for their careful reading and thoughtful critique of our manuscript. Responding to their comments and incorporating their suggestions has led to a substantial improvement of our manuscript. We acknowledge the concerns the reviewers raise regarding the novelty of our investigation. After careful editing of our discussion, we have sought to emphasize the unique mechanistic details our *in vitro* approach allows us to describe, which complements and extends previously published findings using nuclear extracts. We have also made substantial additions to our discussion to incorporate the results from Liu, et al 2025, which was published after our submission and structurally describes a step in the process distinct from what we were able to capture. Again, we are appreciative of the reviewer's thorough comments and believe that our manuscript has been strengthened by addressing their concerns.

Specific Comments:

1. The manuscript should provide detailed information on the purity and potential nuclease contamination of the recombinant NHEJ proteins used. Inclusion of SDS-PAGE or silver-stained gels for all purified components is recommended.

We thank the reviewer for these suggestions. We now include Coomassie/silver stain gels of all purified NHEJ factors used in the experiments below. This figure is now included as S11B in the manuscript.

In order to test for nuclease contamination, we incubated each individual factor with Y30 DNA for 30 min at room temperature in the same buffer used for ligation assays/complex assembly. Samples were subjected to analysis by denaturing TBE/urea PAGE (see methods). Faint bands representing smaller species are visible uniformly across the sample, indicative of some modest degradation of the DNA (likely due to freeze thaw), as opposed to nuclease contamination in any specific protein stock.

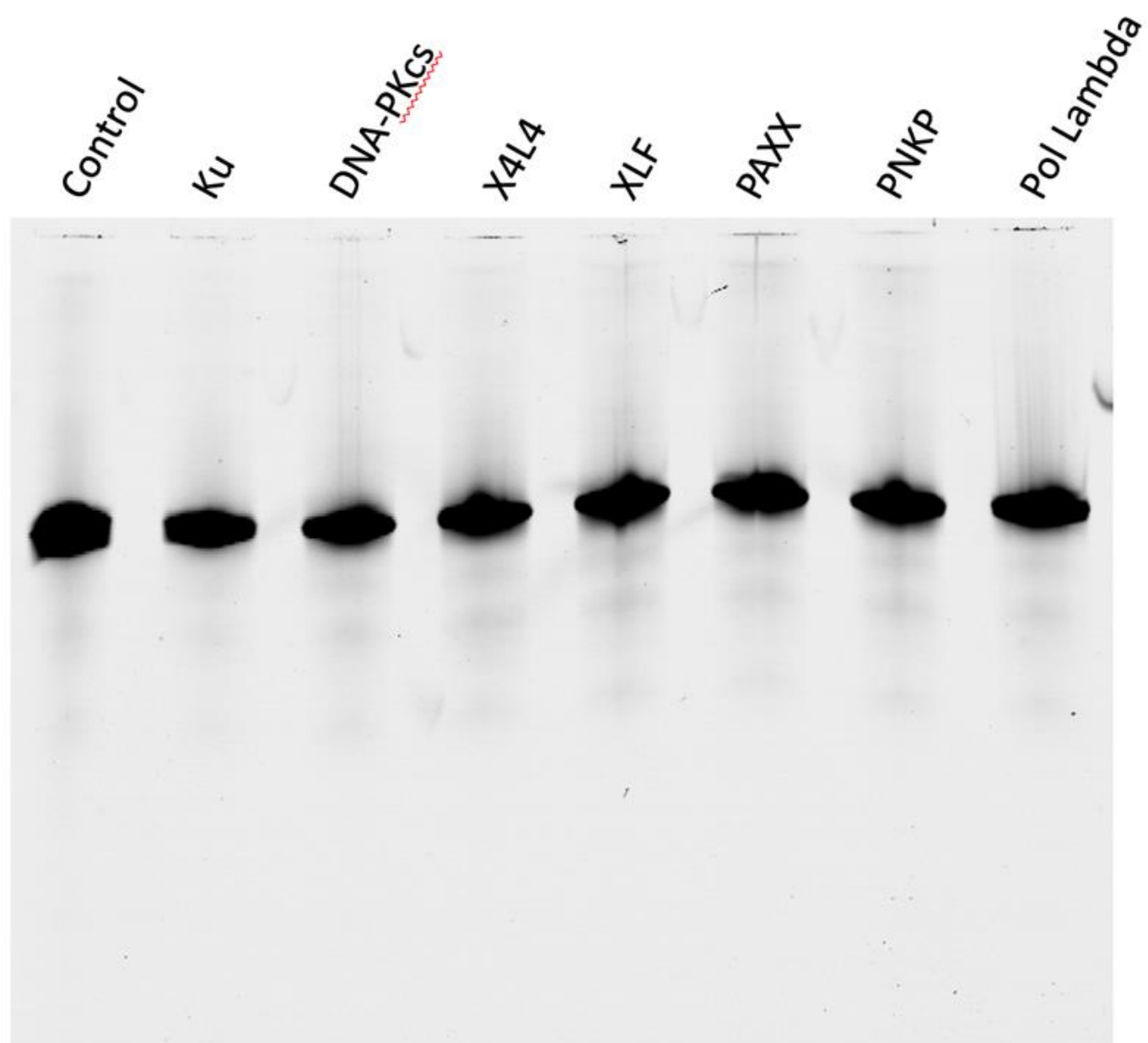

Fig. 1 for reviewers – protein/DNA incubation 30 min at RT in assembly buffer.

2. Western blot analysis should be performed to confirm the presence of all claimed NHEJ proteins, especially Pol λ, in the pull-downs shown in Fig. S1A and S8.

We thank the reviewer for this suggestion. Although, we do not possess reliable antibodies for all 8 different factors we include in our complex assemblies we note that all experiments were performed with purified factors, which were validated by SDS-PAGE during their purification (S10).

To verify the presence of NHEJ proteins via in pull-downs, we have characterized them using negative stain electron microscopy, which have yielded 2D class averages and 3D reconstructions of intact short-range NHEJ complexes, including the BRCT domains of Pol λ, which we were able to capture via cryo-EM. We have included this new electron microscopy data as S1B-E.

So that the connection between the biochemical and structural aspects of our study is more clearly described, we have added the following text to the manuscript at lines 265-272.

“This strategy has added benefit that the sample is amenable for analysis by single particle electron microscopy, allowing samples exhibiting robust ligation to be interrogated structurally to assay complex formation. For example, NS EM of a Pol λ containing pulldown with the components of the SR complex yielded a 3D reconstruction with clearly visible density corresponding to the Pol λ BRCT-Ku70/80 interaction (Fig. S1B-E).”

3. The authors should quantify the stoichiometry of each NHEJ factor in both the pull-down and cryo-EM complexes. The relative abundance of proteins may differ between biochemical and structural assays. Additionally, the rationale for including PAXX in all buffer conditions (reaction, wash, and elution) should be clearly stated.

We thank the reviewer for their comments regarding a potential difference in protein stoichiometry in the pull-down versus cryo-EM experiments. The new negative stain data in S1D-E to demonstrates the presence of fully formed NHEJ complexes representative of the cryo-EM structure. Raw micrographs of pulldowns (Fig. S1B, S6B) show homogenous populations of particles, indicating that the primary species is a fully formed complex, rather than individual factors pulled down on the DNA. Large differences in protein concentration would be observable in the silver stain gels S1A, S10A, and S11A, while the current data shows that factors pull down in approximately 1:1 ratio.

As we reportedly previously (Chen, et al. Sci. Adv. 2023), we suspect that PAXX exchanges more often than the other factors in the complex, and we maintain a low concentration of the factor in all buffer conditions to ensure the presence of the factor in the complex. We have added the following text at lines 571-572 in the methods section to explain:

“...to compensate for its [PAXX’s] rapid removal from the complex in our experimental system, consistent with the protocol established previously.”

4. Could the authors clarify why the Cy5-labeled half was pulled down by the unlabeled half in the presence of X4L4 alone, as shown in Fig. 4B (lane 1)? Regardless of the ligation efficiency, a greater amount of Cy5-labeled DNA was pulled down in the presence of X4L4 and XLF (lane 3) compared to X4L4 alone (lane 1). Does this mean X4L4+XLF can mediate association between the two DNA ends? Similar concerns apply to other pull-down–coupled ligation assays throughout the manuscript. If synapsis efficiency varies, differences in ligation efficiency would be expected as a consequence.

We thank the reviewer for raising the issue that synapsis and ligation efficiencies are not always correlated in our pulldown activity assay. We believe this to be an effect caused by unproductive synapsis, wherein protein-protein interactions hold both DNA ends together, but the assembly is incapable of carrying out ligation. Specifically regarding the X4L4-XLF mediated increase in pulldown efficiency, we suggest that one explanation is both copies of LigIV are bound to either side of the DNA substrate with XLF bridging both sides, which would explain the phenotype in Fig. 4B. The same trend is seen on a different substrate in Fig. 4E lanes 3-4. Furthermore, it has been well documented that XRCC4 and XLF are capable of binding to each other in the absence of other NHEJ factors (PMID 21070942), even forming higher order assemblies (PMID

23442139 & PMID 38898102), which would support the idea that XLF could mediate synapsis of two DNA bound copies of X4L4 independent of Ku. These complexes, while technically synaptic, would likely have very low or no ability to perform ligation without the Ku heterodimer to control LigIV's access to the DSB.

More broadly speaking, there are many phenomena that are likely to confound drawing direct correlation between synaptic efficiency and ligation, particularly *in vitro* where component concentrations are high. Extrapolating from the X4L4-XLF case, any protein-protein interactions of DNA bound species have the potential to form unproductive synaptic complexes. Alternatively, certain factors (Pol λ for example) are known to independently mediate synapsis (PMID 35778389), which could lead to an increase in labeled DNA being pulled down. We also note that it is likely that at least some percentage of the LigIV used in this study was not adenylated, which could lead to fully formed short-range synaptic complexes that are chemically incapable of performing synapsis. For these reasons, we make very limited quantitative claims regarding the results of our ligation assays.

5. The claim that Pol λ is stably recruited to the LR complex (line 238) is primarily based on its enzymatic activity. However, since Pol λ can promote synapsis and gap filling independently (PMID: 35778389), this does not confirm stable recruitment. A gel shift assay with Pol λ alone is needed to substantiate this point.

We thank the reviewer for raising this point. To address this, we include new data showing Pol λ pulling down in roughly stoichiometric ratio with the other factors of the LR complex (Fig. S6E). While we acknowledge that it is possible that some Pol λ may be pulled down through its interaction with DNA, we note that this does not seem to be a substantial enough population to see an enrichment of the polymerase in the elution beyond the roughly 1:1 stoichiometry with the other factors observed via silver stain (Figs. S1A , S6E, S11A). Furthermore, DNA-PK Pol λ structure determined by the Chaplin laboratory (PMID 40328761) shows that the Pol λ BRCT-Ku70/80 interface is not occluded by DNA-PKcs, providing clear evidence that the polymerase can be stably recruited to DNA-PKcs containing NHEJ complexes.

Because of the size of the LR complex (~1.8 MDa) and the number of components it comprises, it would be exceedingly difficult to obtain a clear result from a gel shift assay regarding the presence or absence of a 70 kDa protein. Even if technically feasible, a positive result in this assay would not rule out the binding of the polymerase's catalytic domain to the distal end of the DNA substrate, leading to a false positive result.

6. In lines 88–89, the authors suggest PAXX alone cannot support ligation. However, previous studies (e.g., PMID: 27705800; 31399561) indicate that PAXX can stimulate ligation in the absence of XLF. This should be addressed.

We thank the reviewer for bringing the potentially redundant roles of PAXX and XLF and the discrepancies between our work and previously reported findings. We agree that our language in the original manuscript was too strong regarding this point and have made the following changes at lines 89-91.

"…although its end-bridging activity is not wholly equivalent to XLF, with multiple investigations reporting substantially reduced or complete loss of ligation when the two factors are directly compared[10]"

With regard to the studies mentioned, we would like to acknowledge several critical experimental differences, the most substantial of which is our use of a pulldown to separate the DNA-bound factors from those floating in solution prior to stimulating the ligation reaction. With this in mind, our results are not completely out of line with PMID 27705800 where the ligation activity of X4L4-Ku-PAXX is observed, but only at extremely low efficiency compared to X4L4-Ku-XLF; ligation may be carried out in the presence of PAXX, but it does not form a complex stable enough to survive a pulldown. On the other hand, our system diverges much more than that employed in PMID 31399561 where ligation is investigated using a linearized plasmid (~2700 bp compared to our ~30 bp construct) and a 10 fold excess of protein to DNA (compared to our <2 fold excess). Finally, we would like to point to recently published work that uses PAXX as a negative control compared to XLF in a ligation assay similar to the setup used in PMID 31399561 to further illustrate the prevailing view that PAXX's ability to stimulate ligation does not fully compensate in the absence of XLF.

7. While AlphaFold3 predictions are used to model potential interaction interfaces between NHEJ proteins, experimental validation is lacking. Mutagenesis or truncation analyses are essential to support these computational models.

We thank the reviewer for raising the point that AlphaFold3 predictions are speculative, and we acknowledge that we were not as clear as possible in distinguishing predictions from models build using the map obtained from our cryo-EM experiments. We have identified 3 specific interfaces that the reviewer may be referring to and address them in turn.

1. The Ku70-LigIV-DBD interaction (Fig. 3A) is clearly visible in the cryo-EM data, and AlphaFold3 was only used as a starting point for modeling. To further clarify that this interface was directly derived from experimental data, we include the new supplementary panel, S3E, to clearly show the model fit into the cryo-EM map. Furthermore, for our revisions we have carried out additional fluorescence polarization experiments comparing the affinities of the Artemis and Ku70 peptides binding affinity for this site, revealing modest binding affinities of 1.5-4 µM and 29-30 µM respectively. These data are now included as Fig 3C. We have added the following text to the main text at 195-199:

   "To compare the affinities of these two interacting peptides, we carried out fluorescence polarization on FAM-labelled Artemis$^{486-496}$ and Ku70$^{1-11}$ with the LigIV-DBD. Relatively modest K_D's were calculated for both interactions, with the Artemis-LigIV-DBD at 1.5-4 µM K_D and Ku70-LigIV-DBD at 29-30 µM."

2. Although AlphaFold3 was helpful in identifying the Pol λ NTD-LigIV-BRCT interaction, it was directly observed in the cryo-EM map, which was used to model the peptide. We have included the new supplementary panel S3D to show how the model fits into the cryo-EM map. We further validated this interaction using crosslinking mass spectroscopy (XL-

MS) for our revisions (Fig. 2F), which revealed several crosslinks between the N-terminal region of Pol λ and both LigIV-BRCT1 and XRCC4, providing orthogonal evidence for this interaction. These new data and their analysis (Fig. 2F and Fig S5) have led to several new conclusions, which we discuss at lines 138-161 as follows:

"To further investigate this interaction, we employed crosslinking mass spectroscopy (XL-MS). This revealed a total of 17 crosslinks between the N-terminus of Pol λ and X4L4 (Fig. 2F). Two of these crosslinks were between K733 of the LigIV-BRCT1 subdomain, proximal to the putative binding terminus of the Pol λ-NTR. Surprisingly, a total of 11 crosslinks were observed between the Pol λ-NTR and the ordered C-terminal end of the XRCC4 coiled-coil region (K178, K188, K190, and K197). Another two crosslinks were observed between the N-terminus and K207 of XRCC4, located in the disordered tails. Large number of crosslinks in this area, combined with the fact that only one other was observed anywhere else in the entire NHEJ complex, strongly suggests that the Pol λ-NTR, upstream of its BRCT subdomain, follows the trajectory along the LigIV-BRCT1 subdomain, toward the XRCC4 coiled-coil. Interestingly this strong pattern of crosslinks involving the Pol λ N-terminus is not observed when in complex with X4L4 alone, absent the rest of the NHEJ complex. Only one such crosslink was observed, with K190 of XRCC4. It is possible that interaction between the Pol λ-NTR is only observed in the context of the full complex, due to the slightly different orientation of the LigIV-BRCT1 subdomain, relative to the XRCC4 coiled-coil region, that are observed in the crystal structure of the LigIV tandem BRCT construct in complex with the head and coiled-coil domains of XRCC4[27] (Fig. S5D-E). In the crystal structure, the two LigIV BRCT subdomains are more extensively interacting with the XRCC4 coiled-coil region, but flex slightly more open in the context of the complete NHEJ complex, generating new interfaces with both components of the Ku heterodimer. This may provide a structural underpinning for the N-terminal tail interaction with the polymerases—the BRCT1 subdomain's interface with Ku may be required to present the optimal surface for stable binding."

3. In Fig. 3F-H, we propose a mechanism for polymerase engagement at the DSB based on the predicted structure of LigIV and Pol λ's catalytic domains. In support of this mechanism, we identify several crosslinks our XL-MS experiments between the Pol λ and LigIV's DBD and NTD domains at the predicted interface, which we have mapped onto the predicted state in Fig. 3G. These data are discussed in lines 225-234:

   "Our XL-MS data corroborates the existence of such a state; five crosslinks are observed between the Pol λ catalytic domain and that of LigIV (4 between Pol λ K312 and LigIV K352 in the NTD and one between Pol λ K422 and LigIV K609 just outside of the OBD) (Fig. 3G). The K312-K352 crosslinks are consistent with our AF3 model of the Pol λ catalytic domain bound at the break site, while the K609 crosslink exists in a disordered region between the catalytic and BRCT domains of LigIV, which would position the OBD in an orientation possible in this state, too."

These findings are also complementary with the structure published by Liu et al., which shows Pol µ in precisely this configuration. Correlation to the Pol µ structure are discussed in lines 244-250 of the results and lines 455-465 of the discussion as follows:

(244-250) "While revising this manuscript, the related structure of Pol µ engaged with the short-range complex in precisely this configuration was reported[32]. In this structure, the polymerase's catalytic domain displaces only LigIV's OBD and NTD from the DSB, while the DBD remains in position to stabilize the complex. Given their semi overlapping roles in NHEJ and their shared recruitment mechanism, it is unsurprising that these Pol X's would also carry out end-processing in a similar manner."

(455-465) "As mentioned above, recently published work has demonstrated this is indeed the case for Pol µ[32], suggesting this mechanism is conserved across the Pol X family in their function during NHEJ. It is interesting to note that while we were able to detect Pol λ sampling this configuration through XL-MS, we did not capture a population of particles by electron microscopy substantial enough to produce a 3D reconstruction with the polymerase's catalytic domain bound to the DSB. This may suggest that Pol µ more stably occupies the site or binds to the LigIV-DNA junction with a higher affinity. It is also likely that substrate configuration is a potent modulator of these phenomena as explored in other recently published work[50,51], and further investigation will be needed to fully understand of how all three NHEJ related Pol X's modulate their activity relative to one another."

We do not make any detailed claims regarding any specific residues comprising this state based on the predictions, only that the Pol λ and LigIV catalytic domains both occupy the DSB, which is supported by the fact that the Pol λ catalytic domain is not enough to form a stable complex in the absence of LigIV (Fig. S6).

We would like to add that due to the polyvalent nature of the interactions mediating NHEJ complex formation coupled with the their modest affinities, interrogating these new interactions via mutagenesis or truncation is likely to yield limited insight in our reconstituted system. The results of such experiments would rely on either disruption of complex formation or ligation ability, neither of which we would predict would happen if the Pol λ-NTD or Ku70-CTD were truncated or mutated based on the structural information we have.

8. In lines 220–234, the authors claim that the Ku + X4L4 complex is unable to mediate gap-crossing ligation, and that Pol λ is required to fill the gap prior to ligation. However, as shown in the last two lanes of Fig. 4E, the presence of either dATP or dCTP leads to detectable gap-crossing ligation products, albeit at lower levels than the nick-ligation product. Similarly, gap-crossing ligation products are evident in lanes 2 and 7 of Fig. 4F. These results suggest that a certain degree of gap-crossing ligation may occur independently of Pol λ activity. To substantiate the role of Pol λ in gap filling, the authors should provide sequencing data of the ligation junctions.

We thank the reviewer for identifying the discrepancies in our analyses and the data presented. We agree that our language was too strong in that it implies that we see no gap-crossing ligation, and have made the following changes to the text at lines 279-282 and 297-304.

(279-282) “Surprisingly, ligation is also largely dependent on Pol λ in cases of microhomology flanked by gaps in this system with only faint gap-crossing ligation products visible in conditions absent Pol λ (Fig. 4C).”

(297-304) “We also note that the presence of double ligation products in the last two lanes of Fig. 4C where only a single nucleotide is supplied suggest that Pol λ is capable of incorporating mismatched nucleotides, consistent with previous work identifying the ‘medium fidelity’ inherent to family X polymerases[14].”

We agree from the data that there is gap crossing ligation apparent (lanes 2 and 7 of Fig. 4D – previously 4F). Additionally, with the Pol X family polymerases’ ‘medium fidelity’ (PMID 17631059), we suggest that the single and double ligation products seen in Fig. 4C (previously Fig. 4E) are likely due to incorporation of mismatched bases at the gap, a phenomena we is something we also demonstrated in our system (Fig. S9C).

We explored several options for sequencing our substrates with multiple vendors, but encountered a myriad of technical issues that would prevent satisfactory results. All DNA substrates used in this study share the same Y-shaped structure (see reviewer Fig. 2 for reference), which has significant secondary structure elements that would impair the ability of a polymerase to read through the full sequence. Similarly, the ‘right’ substrates have an internal fluorescent label that would also be an obstacle to the polymerase. Another issue is that the ligation site sits ~50 bp away from the most reasonable sites a primer could be placed, which is not outside of the range where traditional Sanger sequencing methods are inaccurate. Most left-right pairs of substrates are also pseudo-symmetrical in regards to sequence, such that there is no pair of primers that could sufficiently amplify an entire doubly ligated product.

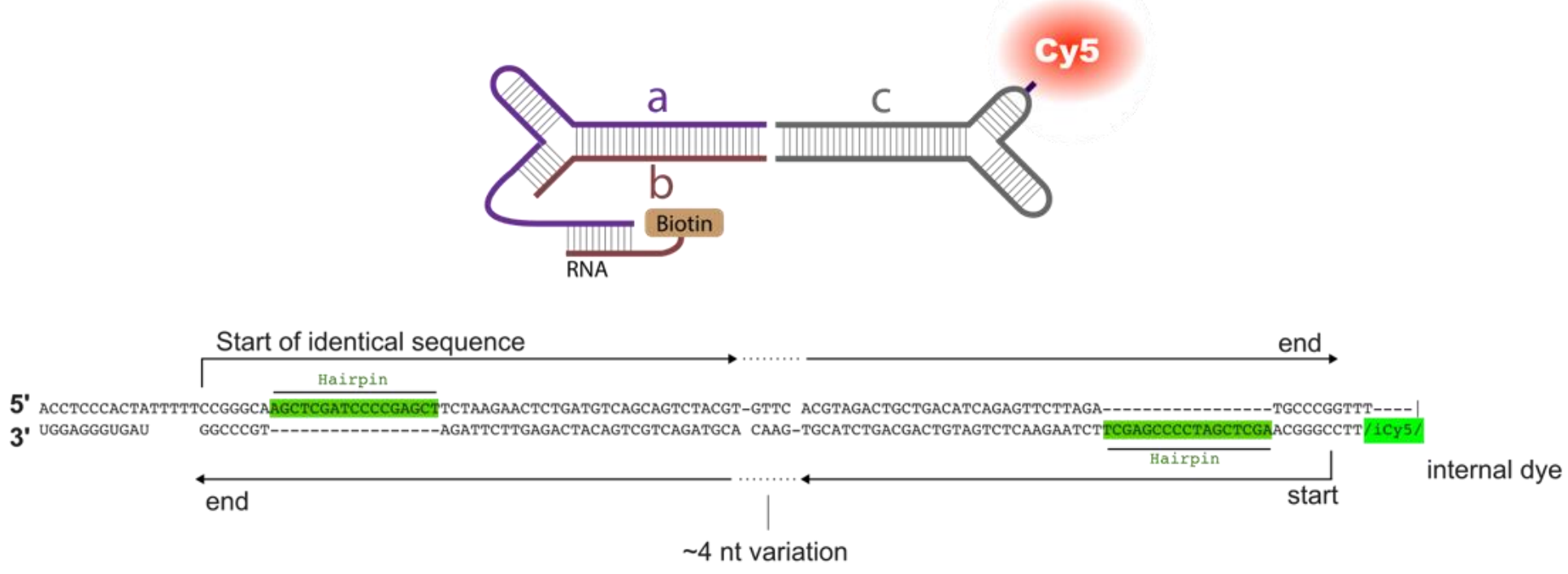

Fig. 2 for reviewers – DNA substrate design. Top shows overall shape after annealing (adapted from Fig. 4A) and bottom shows sequence specific information.

Although directly sequencing the substrates used for our biochemical reconstitutions is not feasible, we also considered redesigning certain critical substrates so that they would be more amenable for sequencing to address concerns raised by reviewers, but we quickly realized that this would introduce issues related to complex assembly, decorrelating the biochemical and

structural aspects of the study. Removing the Y-shaped elements at either end of the substrate would relieve the steric block preventing Ku from loading on both ends of the DNA, and extending the duplex DNA so that the ligation site is farther away from the primers could lead to multiple Ku binding onto the same DNA (PMID 39058590).

Despite these issues, we decided to move forward with a pilot experiment to see what sequencing data would result from a ligated substrate, as well as testing the feasibility of sequencing the products of this system more broadly. We incubated Y30BE DNA with T4 ligase at a concentrations mirroring those used for our electron microscopy and activity assays, visualizing the products with TBE/Urea denaturing PAGE. Prominent ligation products were visible, but attempts to purify them from the gel resulted in extremely low yields (<5 ng/μL) and poor purity profiles. We were left with the conclusion that only substantially scaling up the reaction by orders of magnitude would yield enough material, but this would almost certainly lead to concentration related effects in complex assembly. Given these technical challenges, sequencing of products of our ligation assays is not feasible, but we believe that the claims we make after the changes to the text detailed above are sufficiently supported by the data.

9. The assertion (lines 243–248) that DNA-PKcs can use dATP as a phosphorylation cofactor lacks direct supporting evidence. Experimental confirmation is necessary.

We agree with the reviewer that this claim warranted more experimental evidence. To prove this, we specifically investigated the ability of dATP to support DNA-PKcs phosphorylation at S2056, which has been shown to be indicative of the kinase's activation during NHEJ (PMID 15677476). These data are now included in Fig. S8, and we discuss the results at lines 312-318:

"To determine whether DNA-PK is active in the presence of dATP, purified DNA-PK was incubated under standard kinase assay conditions with equimolar amounts of either ATP or dATP (Fig. S8A-B). The results show that DNA-PK is catalytically active and able to support DNA enhanced autophosphorylation on serine 2056 in the presence of either ATP or dATP. Similar results were obtained using dATP from two different suppliers (Thermo Scientific (a) and Invitrogen (b) in Fig. S8A-B, respectively), and in purified DNA-PKcs and Ku70/80 from two separate batches of HeLa cells. We also confirmed that this phosphorylation was sensitive to the DNA-PKcs inhibitor Nu7441 (Fig. S8C)."

10. The claim that PNKP forms a stable complex with core NHEJ factors (lines 271–272) is not supported by a clear shift or enrichment in the gel shown in Fig. S7D. This should be validated with further data.

We thank the reviewer for this comment regarding the stable recruitment of PNKP to the SR complex. While the data provided in the original manuscript show that it is pulled down with the other factors (Fig. S10A), we acknowledge that an alternative explanation for the data was that it was being pulled down by the DNA, independently of the other NHEJ proteins, with the glycerol-gradient experiment not showing a clear shift in Fig. S10B. To test this we performed a

side-by-side pulldown of PNKP by itself, and with the other NHEJ factors, to show that protein-protein interactions are responsible for the continued presence of PNKP in the elution (Fig. S10C). The absence of PNKP in the pulldown with DNA indicates that a protein-protein interaction is necessary for it to be stably recruited to a DSB during NHEJ. We have added the following relevant text to the manuscript at lines 348-359:

"To test whether the end-processing factor PNKP forms a similarly stable complex with the core NHEJ factors, we carried out *in vitro* reconstitution of the SR complex, separating the DNA bound species by both graFix (Supplementary Fig. 7B) and pulldown (Supplementary Fig. 7C). Although there was no marked peak shift in the graFix experiment, we observed that PNKP requires other NHEJ factors to be retained in the elution of a pulldown (Fig. S9C), indicating that protein-protein interactions are responsible for its recruitment to the site of DNA damage during NHEJ."

11. The authors should explain why ATP appears to inhibit 5' OH phosphorylation, as suggested by reduced ligation in the final lane.

Although it is not clear which lane is referenced in this comment, as discussed in our response to comment 4, there are several factors that can confound the amount of DNA pulled down in the ligation assays, which could also manifest as an observed change in the amount of ligated product when comparing across lanes. We have been deliberately conservative in making any quantitative claims regarding them.

12. The conclusion that PNKP activity occurs specifically within the short-range synaptic complex is not adequately supported. Observing specific ligation in PNKP-containing reactions does not rule out activity prior to or outside of the SR synapsis. Additional temporal or structural evidence is needed.

We agree with the reviewer that our data do not rule out PNKP activity outside of the short-range synaptic complex and note that the enzyme is capable of binding to and performing chemistry on DNA on its own and outside of the context of NHEJ (PMIDs 22055185, 39395804, 22130663). Our claim is that once the short-range complex is formed, PNKP retains access to the DSB and can perform chemistry at the DNA termini.

During complex assembly, PNKP was added after LigIV, meaning that the short-range complex is fully assembled, with the LigIV catalytic domain trapped onto the DNA. We see no evidence that PNKP disassociates this complex to perform end-processing, and this would almost certainly manifest as a decrease in complex assembly efficiency, which we do not observe. We have added the following explanation at lines 371-373:

"By adding PNKP after the LigIV during complex assembly, we ensured that the end-processing enzyme was encountering a fully formed short-range complex, within which it would coordinate its activity."

As we note in the discussion prior to short-range synapsis, DNA-PKcs sterically occludes the DNA ends, preventing aberrant end chemistry. Absent significant structural coordination with

DNA-PKcs, such has been shown for Artemis, it is unlikely that any factors would have access to the DNA prior to the kinase's disassociation from the complex and the transition to SR synapsis.

Reviewer #2 (Remarks to the Author):

This manuscript by Vogt et al seeks to understand the mechanism by which end-processing occurs within the SR complex during NHEJ. To this end, they solved a CryoEM structure of the SR complex in which the processing factor Pol λ is bound. In the structure, LigIV adopts an 'open' conformation, which the authors speculate would allow end-processing by accessory NHEJ factors. In addition, the authors show that they can preassemble NHEJ complexes containing both PNKP and Pol λ that carry out end joining reactions that require the activity of both processing factors. Overall, the work is a rather modest step forward in our understand of NHEJ end processing given recent published structures by the Chaplin (Frit Nature Communications 2025) and Yang labs (Liu Nature 2025). Given this and other major concerns listed below, I do not believe the work merits further consideration at Nature Communications.

We thank the reviewer for their candid assessment of our work. We have carefully read their concerns and have used our revisions to clarify the issues they raise regarding our methods and conclusions, which has substantially strengthened our manuscript. We note that the manuscript Liu, Nature 2025 was published after our manuscript was submitted, and that all three of our studies resolve distinct steps of polymerase engagement during NHEJ. In our revisions, we have made changes to the text to highlight the complementarity of all of these findings. We appreciate the reviewers input and believe our work has improved in addressing their concerns.

Major Concerns:

1.) In the abstract (lines 32-33) the authors assert they have 'uncovered the mode of Pol λ recruitment to the complex'. This is a bold claim given prior work in the literature including (Frit Nat Comm 2025; Lee JBC 2004; Fan Biochem and Biophys Res Comm 2004; McElhinny Mol Cell 2005).

We agree with the reviewer that more conservative wording is appropriate. We have changed the wording in lines 31-33 as follows:

"… we determined the cryo-EM structure of the NHEJ specific polymerase Pol λ bound to the short-range synaptic complex, **detailing** the mode of its recruitment to the complex…"

2.) The newly presented CryoEM structures show no evidence of Pol λ or PNKP engagement with the DNA, only with other proteins in the SR complex. In addition, all contacts between proteins were previously known and this is simply a composition of previously found contacts. The newly found open conformation is intriguing from an end-processing point of view, but no experiments were performed to validate the existence of the state or the dependence of end-

processing within the SR complex on this open state. It's possible this state may simply represent an intermediate in the removal of LigIV from the SRC to allow processing, as presented in Wei Yang's recent work (Nature, June 2025).

We thank the reviewer for their appraisal of our cryo-EM structure. We would like to highlight that the Ku70-CTD-LigIV-DBD interaction detailed in Fig. 3B and the Pol lambda NTD-LigIV-BRCT interaction detailed in Figs. 2C-D were both unreported at the time of submission, the latter of which is still unique to our work.

In order to validate our structure we carried out crosslinking mass spectroscopy (XL-MS), which confirmed interaction between the catalytic domains of Pol lambda and LigIV. As shown in Fig. 3G, several crosslinks were observed near the predicted interface between these factors (Fig. 3F). We discuss these results in lines 225-234:

"Our XL-MS data corroborates the existence of such a state; five crosslinks are observed between the Pol λ catalytic domain and that of LigIV (4 between Pol λ K312 and LigIV K352 in the NTD and one between Pol λ K422 and LigIV K609 just outside of the OBD) (Fig. 3G). The K312-K352 crosslinks are consistent with our AF3 model of the Pol λ catalytic domain bound at the break site, while the K609 crosslink exists in a disordered region between the catalytic and BRCT domains of LigIV, which would position the OBD in an orientation possible in this state, too."

Given that the closed conformation of LigIV completely sterically occludes access to the DSB and the crosslinked sites, this intermediate open state would explain how the polymerase gains access to both of these. One we also note is that structural studies that reporting a similar open LigIV conformation have done so by DNA ends that are artificially blocked by 3'-dideoxynucleotide-terminated upstream primers (PMID 29980672 and PMID 40500445), and we observed this state on noncompatible overhang DNA, all of which support the notion that the ligase assumes this state when the DSB cannot be directly ligated and requires end-processing.

We strongly agree with the reviewer's suggestion that the state represents a structural intermediate in NHEJ, and this is exactly the point we sought to make in Fig. 3H. The nuance we would add is that the LigIV catalytic domain does not completely disassociate from the DSB during end-processing, as we suggest in our manuscript based on structure prediction, XL-MS data, and the fact Pol lambda is not able to replace LigIV in forming stable short-range complex (Fig S6). This conclusion is further supported by PDB 9CQ3 as reported by Wei Yang's group, who captured Pol μ in precisely the same arrangement as predicted in Figs. 3F-H.

3.) A substantial number of figure panels is Alphafold modeling (Figures 2D, 2E, 3B, 3E, 5A) yet none of these novel interactions have been confirmed with any mutational analysis in vivo or in vitro. For example, if the Pol λ contacts between Pol λ and LigIV are important for SR complex formation / recruitment / processing, mutation of Pol λ in that area should abolish activity. As acknowledged by the authors the proposed interaction between TdT/Pol μ and LigIV seems quite tenuous (lines 141-142; Figure 2G/H) and most likely is an artifact by Alphafold. Including PAE maps would also help with their analysis of these potential interactions. Doing a

simple pull-down assay between TdT/LigIV would also help to validate this proposed interaction.

We agree with the reviewer that conclusions drawn from AlphaFold3 require additional validation. As noted in the response to point #2, our new XL-MS data corroborates the AF3 predictions shown in Figs. 3F-H.

Our new XL-MS data confirms interactions between Pol lambda's N-terminal region and both LigIV and XRCC4 (Fig. 2F). These crosslinks disappeared in the absence of other factors, suggesting that the Pol lambda-Ku interaction is necessary for recruitment to the complex. Furthermore, we carried out fluorescence polarization assays on the LigIV-BRCT1 domain and all three Pol X NTR's but did not observe any binding, indicating that this interaction is extremely weak and present only when the polymerases are interacting with Ku via their BRCT domain. For this reason, mutating the NTR of Pol lambda is unlikely to affect complex formation or the repair activities of the repair complex *in vitro.* Similarly, because the TdT-BRCT-Ku70/80 interaction is the predominant factor recruiting the polymerase to the NHEJ complex (PMID 10570175), a TdT/LigIV pulldown would be insufficient to prove or disprove the existence interaction. In contrast to playing fundamental roles in NHEJ complex assembly and end-processing, we speculate that these interactions may play a role in regulating the process, particularly *in vivo* where there may be more competition for this site on the complex. We have added the following lines in the manuscript to discuss the new experimental data as well as clarifying the potential significance of this interaction in lines 138-161 of the text:

"To further investigate this interaction, we employed crosslinking mass spectroscopy (XL-MS). This revealed a total of 17 crosslinks between the N-terminus of Pol λ and X4L4 (Fig. 2F). Two of these crosslinks were between K733 of the LigIV-BRCT1 subdomain, proximal to the putative binding terminus of the Pol λ-NTR. Surprisingly, a total of 11 crosslinks were observed between the Pol λ-NTR and the ordered C-terminal end of the XRCC4 coiled-coil region (K178, K188, K190, and K197). Another two crosslinks were observed between the N-terminus and K207 of XRCC4, located in the disordered tails. Large number of crosslinks in this area, combined with the fact that only one other was observed anywhere else in the entire NHEJ complex, strongly suggests that the Pol λ-NTR, upstream of its BRCT subdomain, follows the trajectory along the LigIV-BRCT1 subdomain, toward the XRCC4 coiled-coil. Interestingly this strong pattern of crosslinks involving the Pol λ N-terminus is not observed when in complex with X4L4 alone, absent the rest of the NHEJ complex. Only one such crosslink was observed, with K190 of XRCC4. It is possible that interaction between the Pol λ-NTR is only observed in the context of the full complex, due to the slightly different orientation of the LigIV-BRCT1 subdomain, relative to the XRCC4 coiled-coil region, that are observed in the crystal structure of the LigIV tandem BRCT construct in complex with the head and coiled-coil domains of XRCC4[27] (Fig. S5D-E). In the crystal structure, the two LigIV BRCT subdomains are more extensively interacting with the XRCC4 coiled-coil region, but flex slightly more open in the context of the complete NHEJ complex, generating new interfaces with both components of the Ku heterodimer. This may provide a structural underpinning for the N-terminal tail interaction with the

polymerases—the BRCT1 subdomain's interface with Ku may be required to present the optimal surface for stable binding."

To highlight the experimental data Pol lambda NTD-LigIV-BRCT interaction identified, we have included Fig. S3D to demonstrate the presence of this peptide in or cryo-EM map. We also note that density at the same position is present in the cryo-EM map deposited by Liu et al., which we suspect corresponds to a similar polymerase NTD-LigIV-BRCT interaction, although the density was left unmodeled in the deposited structure (Reviewer Fig. 2). By aligning the predicted LigIV-Pol μ complex to 9CQ3's LigIV-BRCT1, we show that the Pol μ peptide clearly accounts for this unmodeled density.

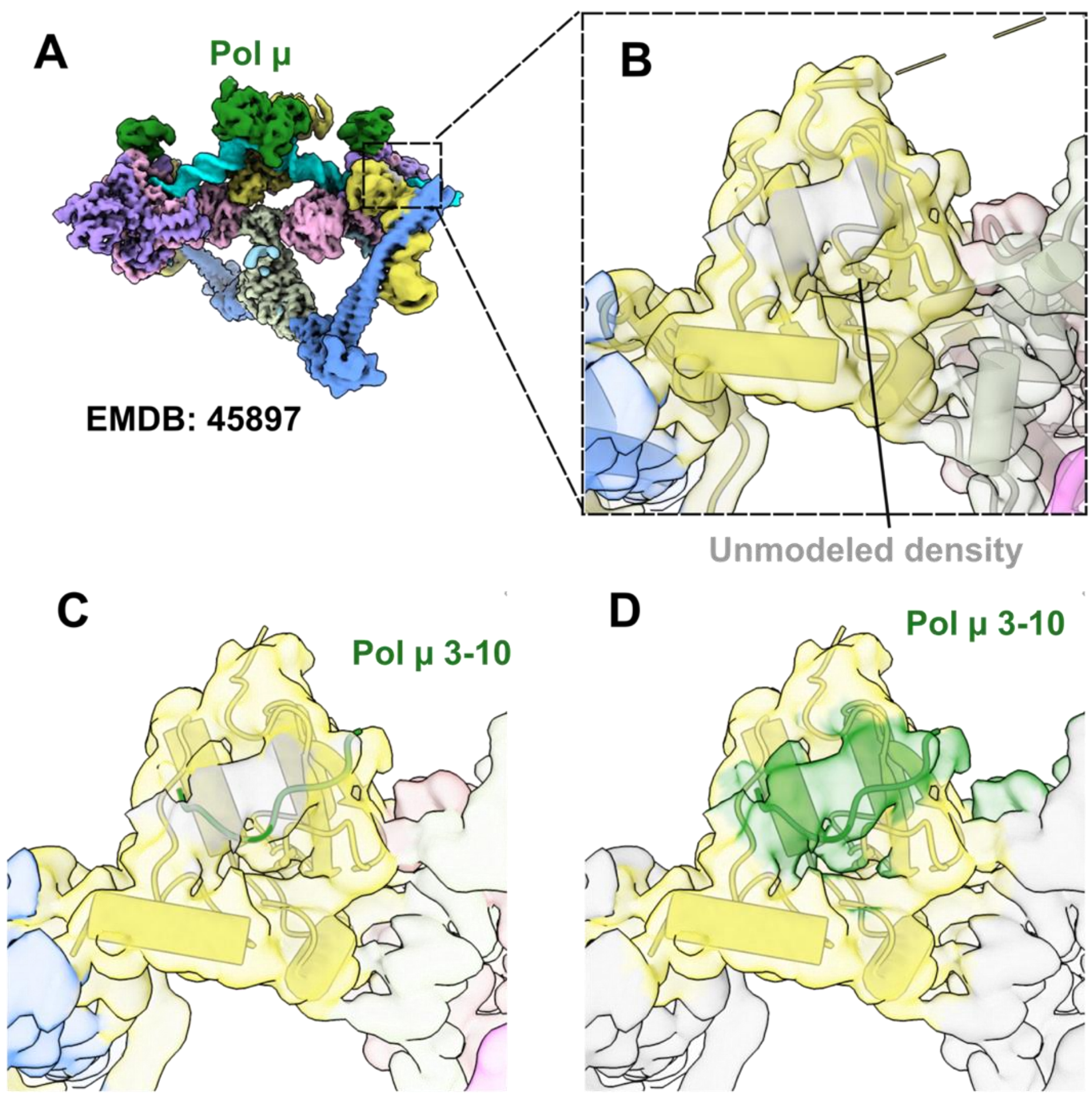

Fig. 3 for reviewers – Pol μ-NTR interaction with LigIV-BRCT1

In addition to these new data, we have added Fig. S5 where we provide confidence metrics for all predictions. We have carefully reviewed the figures and now all figures are either explicitly labeled as AF3 predictions or predicted structural information is blurred to avoid misrepresentation. In Figs. 2E and 4F (previously 5A), AlphaFold3 was used as a convenient way to generate PDB files for the full length proteins, which are shown to illustrate the flexible attachment of the factors to the complex, not to give the impression that all components were

fully modeled, which we sought to communicate by their transparent coloring. To make this more clear, we have added a slightly blurred effect to portions of the proteins not observed in the cryo-EM map, which retains the realistic dimensions of the factors but further distinguishes them from the portions of the models that were generated from experimental data.

We also note that the model shown in Fig. 2D was built using our cryo-EM map and Fig. 3A (Fig. 3B in original manuscript) was generated from a crystal structure of Artemis Lig-IV (PDB 3WIG, PMID 23523427). So that this is clear, we have labeled Fig. 3A with the corresponding PDB code.

5.) There are several issues with the in vitro assays performed for this work:
1.) The authors have also not quantified any of the joining making it difficult to compare the activity between their assays and other previously published work.

We thank the reviewer for suggesting that quantitative analysis of our *in vitro* ligation assays may help in their analysis; please see representative data in Reviewer Fig. 4 and all data in Reviewer Table 1. Products generally account for between 3-15% of the signal in lanes that they appear. These are approximate to results in the similar system reported in PMID: 40500445 and somewhat less than PMID 31399561. We could not directly compare with many of the studies cited in our manuscript, due to their lack of quantitative analysis (PMIDs 15574326, 17290226**,** 31862156**,** 28696258).

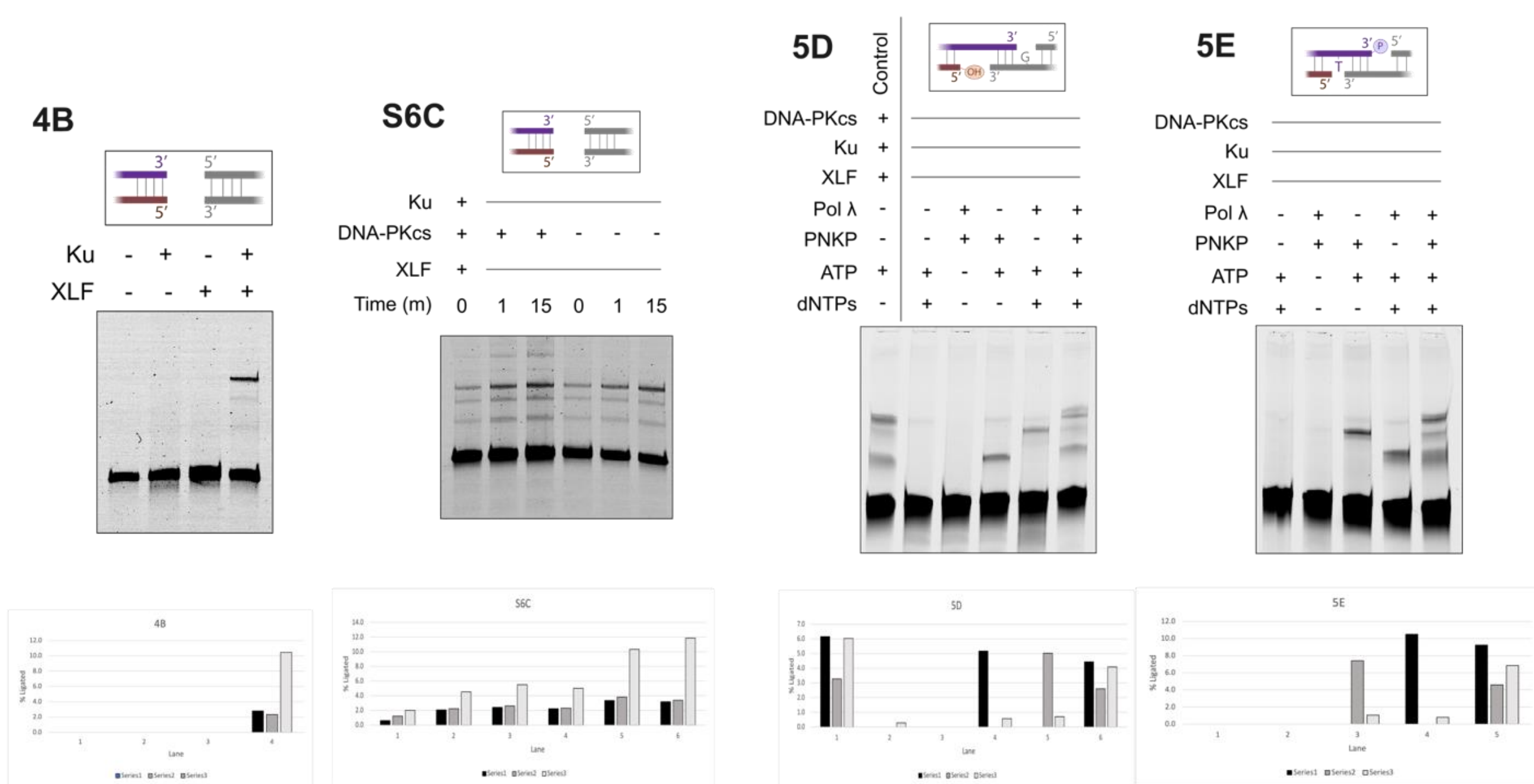

Figure 4 for reviewers – quantification of *in vitro* ligation assays. Intensity was measured in Fiji and product amounts were normalized for the entire amount of DNA in each lane.

Although we provide these data for the reviewers, we avoid quantitative analysis of these data and in our manuscript, instead relying on them to qualitatively confirm and extend our structural studies. Direct comparisons of data generated by different experimental setups can often be misleading unless great attention is given to understanding the experimental conditions. Simply changing the relative concentrations of factors and DNA can drastically change the amounts of repaired DNA in reconstituted systems (for example compare PMID 27705800 and PMID 31399561). Our approach introduces additional caveats; using a magnetic beads-based pulldown to separate the complex assembly and ligation steps leads to differences in the amount unligated substrate being retained on the beads depending on the formation of unproductive assemblies that can capture the right half of the substrate but fail to carry out ligation. It is also likely that some DNA is nonspecifically trapped on the beads, a phenomena that could change based on the particular DNA substrate. For these reasons we are very conservative about any quantitative claims. While we agree with the reviewer that an extensive quantitative analysis of end-processing dependent ligation on different substrates would be of interest, we acknowledge the limitations of our approach in this regard and hope that our findings can help guide studies with that have this aim.

**Fig. 4**				
**4B**				
**Lane**	**%unligated**	**%product1**	**%product2**	**%product3**
**1**	100.0	0.0	0.0	0.0
**2**	100.0	0.0	0.0	0.0
**3**	100.0	0.0	0.0	0.0
**4**	84.4	2.8	2.3	10.5
**4C**				
**Lane**	**%unligated**	**%product1**	**%product2**	**%product3**
**1**	56.3	23.6	5.7	14.4
**2**	97.0	3.0	0.0	0.0
**3**	91.5	8.5	0.0	0.0
**4**	93.1	6.9	0.0	0.0
**5**	100.0	0.0	0.0	0.0
**6**	74.2	13.9	2.6	9.4
**7**	80.4	5.7	10.2	3.7
**8**	60.4	37.4	0.8	1.3
**9**	62.7	36.2	0.0	1.1
**4D**				
**Lane**	**%unligated**	**%product1**	**%product2**	**%product3**
**1**	85.4	6.3	2.5	5.7
**2**	97.7	1.0	0.0	1.3
**3**	100.0	0.0	0.0	0.0
**4**	89.3	9.0	0.0	1.7
**5**	100.0	0.0	0.0	0.0
**6**	87.4	5.7	2.0	4.9
**7**	95.2	3.3	0.0	1.5
**8**	97.3	1.4	0.0	1.3
**9**	94.1	1.3	2.7	2.0
**10**	91.2	3.5	1.4	4.0
**4E**				
**Lane**	**%unligated**	**%product1**	**%product2**	**%product3**
**1**	81.9	15.4	2.7	0.0
**2**	95.7	2.6	1.7	0.0
**3**	100.0	0.0	0.0	0.0
**4**	100.0	0.0	0.0	0.0
**5**	100.0	0.0	0.0	0.0
**6**	81.5	3.8	13.0	1.7
**7**	80.1	7.5	8.7	3.8
**4G**				
**Lane**	**%unligated**	**%product1**	**%product2**	**%product3**
**1**	90.8	3.9	1.8	3.5
**2**	92.4	0.0	6.4	1.3
**3**	100.0	0.0	0.0	0.0
**4**	86.7	4.6	3.1	5.6
**5**	88.4	10.6	0.0	1.0
**6**	100.0	0.0	0.0	0.0
**7**	83.6	5.7	3.2	7.5
**8**	100.0	0.0	0.0	0.0
**9**	100.0	0.0	0.0	0.0
**10**	90.1	2.9	2.5	4.5

**Fig. 5**				
**5A**				
**Lane**	**%unligated**	**%product1**	**%product2**	**%product3**
**1**	90.1	0.0	0.0	9.9
**2**	95.4	0.0	0.0	4.6
**3**	94.3	0.0	0.0	5.7
**4**	87.8	2.4	2.0	7.8
**5B**				
**Lane**	**%unligated**	**%product1**	**%product2**	**%product3**
**5**	92.8	0.0	0.0	7.2
**6**	96.4	0.0	0.0	3.6
**7**	82.9	0.0	11.3	5.9
**8**	69.7	4.5	4.1	21.7
**5C**				
**Lane**	**%unligated**	**%product1**	**%product2**	**%product3**
**2**	100.0	0.0	0.0	0.0
**3**	98.6	1.4	0.0	0.0
**4**	97.2	0.9	0.8	1.1
**5**	92.1	2.3	1.5	4.1
**5D**				
**Lane**	**%unligated**	**%product1**	**%product2**	**%product3**
**1**	84.5	6.2	3.3	6.0
**2**	99.7	0.0	0.0	0.3
**3**	100.0	0.0	0.0	0.0
**4**	94.2	5.2	0.0	0.6
**5**	94.3	0.0	5.0	0.7
**6**	88.9	4.4	2.6	4.1
**5E**				
**Lane**	**%unligated**	**%product1**	**%product2**	**%product3**
**7**	100.0	0.0	0.0	0.0
**8**	100.0	0.0	0.0	0.0
**9**	91.6	0.0	7.4	1.1
**10**	88.7	10.5	0.0	0.8
**11**	79.3	9.2	4.6	6.8

**Fig. S6**				
**S6A**				
**Lane**	**%unligated**	**%product1**	**%product2**	**%product3**
**1**	87.0	1.1	1.2	10.6
**2**	87.1	0.9	1.1	11.0
**3**	100.0	0.0	0.0	0.0
**S6B**				
**Lane**	**%unligated**	**%product1**	**%product2**	**%product3**
**1**	93.2	1.6	1.9	3.3
**2**	100.0	0.0	0.0	0.0
**3**	100.0	0.0	0.0	0.0
**S6C**				
**Lane**	**%unligated**	**%product1**	**%product2**	**%product3**
**1**	96.1	0.6	1.2	2.0
**2**	91.2	2.1	2.2	4.5
**3**	89.4	2.4	2.6	5.5
**4**	90.4	2.2	2.3	5.0
**5**	82.5	3.4	3.8	10.3
**6**	81.6	3.2	3.4	11.9

**Fig. S8**				
**S8A**				
**Lane**	**%unligated**	**%product1**	**%product2**	**%product3**
**1**	100.0	0.0	0.0	0.0
**2**	93.4	1.6	1.8	3.2
**3**	100.0	0.0	0.0	0.0
**4**	93.8	1.8	1.5	2.9
**5**	100.0	0.0	0.0	0.0
**6**	94.6	1.6	1.2	2.5
**7**	99.6	0.4	0.0	0.0
**8**	86.0	2.4	3.5	8.1
**S8B**				
**Lane**	**%unligated**	**%product1**	**%product2**	**%product3**
**1**	97.9	0.0	1.5	0.6
**2**	74.2	6.0	5.2	14.7
**3**	100.0	0.0	0.0	0.0
**4**	78.6	4.9	4.6	12.0
**5**	100.0	0.0	0.0	0.0
**6**	74.3	7.7	6.0	12.0
**7**	100.0	0.0	0.0	0.0
**8**	80.7	3.7	6.0	9.6
**S8C**				
**Lane**	**%unligated**	**%product1**	**%product2**	**%product3**
**1**	98.7	0.0	0.9	0.4
**2**	94.5	1.3	1.7	2.5
**3**	98.3	0.0	0.8	0.9
**4**	97.0	0.7	1.1	1.2
**5**	90.1	1.9	4.1	3.9
**6**	97.9	0.0	1.0	1.1
**7**	97.3	0.7	0.9	1.1
**8**	96.6	0.8	1.2	1.4
**9**	96.6	0.9	1.2	1.4
**10**	96.2	0.9	1.3	1.6
**S8D**				
**Lane**	**%unligated**	**%product1**	**%product2**	**%product3**
**1**	60.1	0.0	11.8	28.0
**2**	87.7	4.9	6.2	1.2
**3**	70.3	9.7	10.2	9.8
**S8E**				
**Lane**	**%unligated**	**%product1**	**%product2**	**%product3**
**1**	61.0	0.0	38.7	0.3
**2**	86.3	0.7	12.6	0.4
**3**	90.1	0.0	3.8	6.1
**S8F**				
**Lane**	**%unligated**	**%product1**	**%product2**	**%product3**
**1**	74.8	3.3	2.4	19.5
**2**	99.6	0.0	0.0	0.4
**3**	84.0	0.9	2.9	12.2
**4**	100.0	0.0	0.0	0.0
**5**	91.7	2.8	1.9	3.7
**6**	93.1	1.5	1.4	4.0

Table 1 for reviewers – quantification of *in vitro* ligation assays

2.) Gel loading of nearly all presented gels is uneven and the banding pattern suggests a form of degradation or lack of homogeneity in sample preparation.

We appreciate the reviewer's concern that our gel figures that might lead readers to be skeptical of our results. We have assessed our protein factors for nuclease contamination by incubating each factor individually with DNA for 30 min at room temperature (reviewer Fig. 1). Some faint degradation products are seen uniformly across all lanes, including in the control, which can be attributable to freeze-thaw not nuclease contamination, and we find it unlikely that their presence alters our analysis.

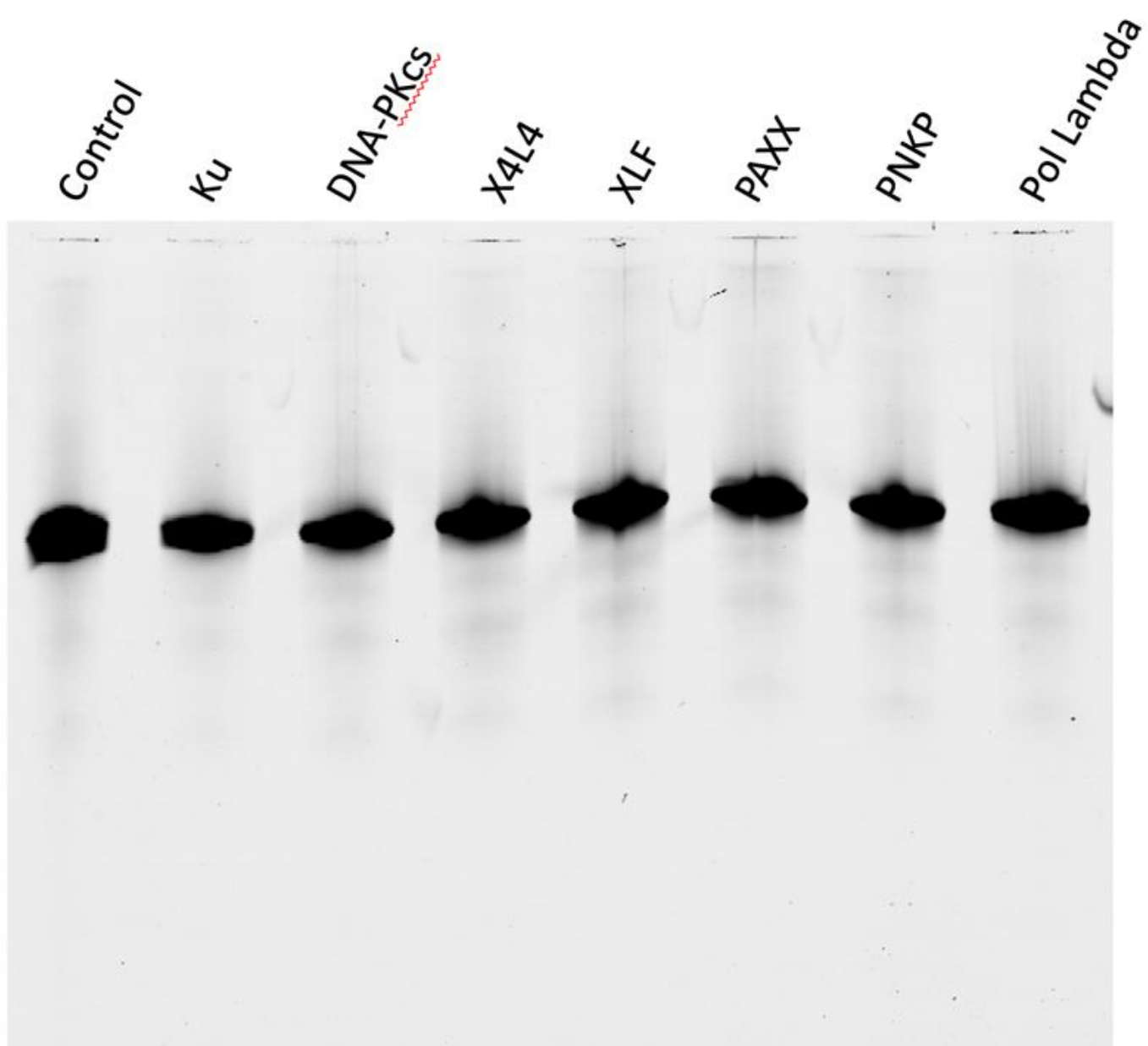

Fig. 1 for reviewers – protein/DNA incubation 30 min at RT in assembly buffer.

Although we acknowledge that slight variations in gel loading due to human error are inevitable, the experiments were carried out carefully, with care given to the amount of each component added to reactions and loaded onto the gels. We suspect that the reviewer is noting the discrepancies in the amount of material pulled down, prior to the start of ligation. For example, there are notable differences in the amounts of unligated product in Fig. 4B, which we attribute to the presence of different NHEJ factors forming assemblies of varying stability, leading to more or less labeled DNA being pulled down. When the same combination of protein components and DNA substrate are included, the material on the gel is much more uniform - see Fig. S7.

3.) The authors make conclusions about the timing of events for joining on the top strand vs the bottom strand without doing a time course. A time course is critically important and essential to draw these types of conclusions.

We have reviewed the draft of our manuscript carefully for claims regarding the timing of events and have made the following changes at line 296:

'In our system, as opposed to substrates with no microhomology, single ligation on substrates with terminal base-pairing is possible (Fig. 4C), indicating that in the SR complex LigIV can repair nicks near gaps independently of Pol λ activity, so long as they are flanked by base-paired nucleotides.'

We thank the reviewer for raising this issue regarding conclusions drawn regarding the timing of events. As we show in Fig S7C, once fully assembled, the complex carries out repair on the order of seconds, with the rate limiting factor being the assembly of the complex. We fully agree that additional temporal information on these NHEJ assemblies would be insightful, but techniques capable of probing these short time periods are outside of our expertise, and would probably warrant their own standalone investigation.

4.) The banding pattern in most gels (Figures 4C, 4D, 4F, 5B, 5D, 5E, 6A, 6B, 6C, 6D) shows a doublet at the fully joined species without any given explanation. Sequencing of these products would vastly help the interpretation of the data. In addition, it looks like some of the products are mislabeled. For example, in Figure 6A, it appears the singly ligated product is the band shown in lane 3, not the small and diffuse band as labeled; again, sequencing of the products would resolve these issues.

We thank the reviewer for their comments regarding the banding patterns in our gels. In order to test the feasibility of sequencing from our experimental setup, we incubated Y30BE DNA with T4 ligase in amounts commensurate with our electron microscopy and activity assays. Although ligation products were visible attempts at isolating products from the gel resulted in extremely low yields (<5 ng/μL) with unconvincing purity profiles.

After discussion with multiple sequencing vendors, all of which concluded that commercially available sequencing techniques would be unlikely to yield interpretable results. As diagrammed below, the substrates for DNA assembly have significant secondary structure, and all that were used for the ligation assays had an internal fluorescent label, both of which would likely negatively impact a polymerase's processivity. We also note that the distance of the ligation site is, at the most, roughly 50 bp from where a sequencing primer could be placed, which is still within the range for significant sequencing fidelity loss. Additionally, due to the design of the substrates, which is pseudo symmetrical, it is impossible to design primers that fully amplify a doubly ligated product. For these reasons, it is likely that sequencing data would be of low quality and further confound interpretation of these results rather than clarify them.

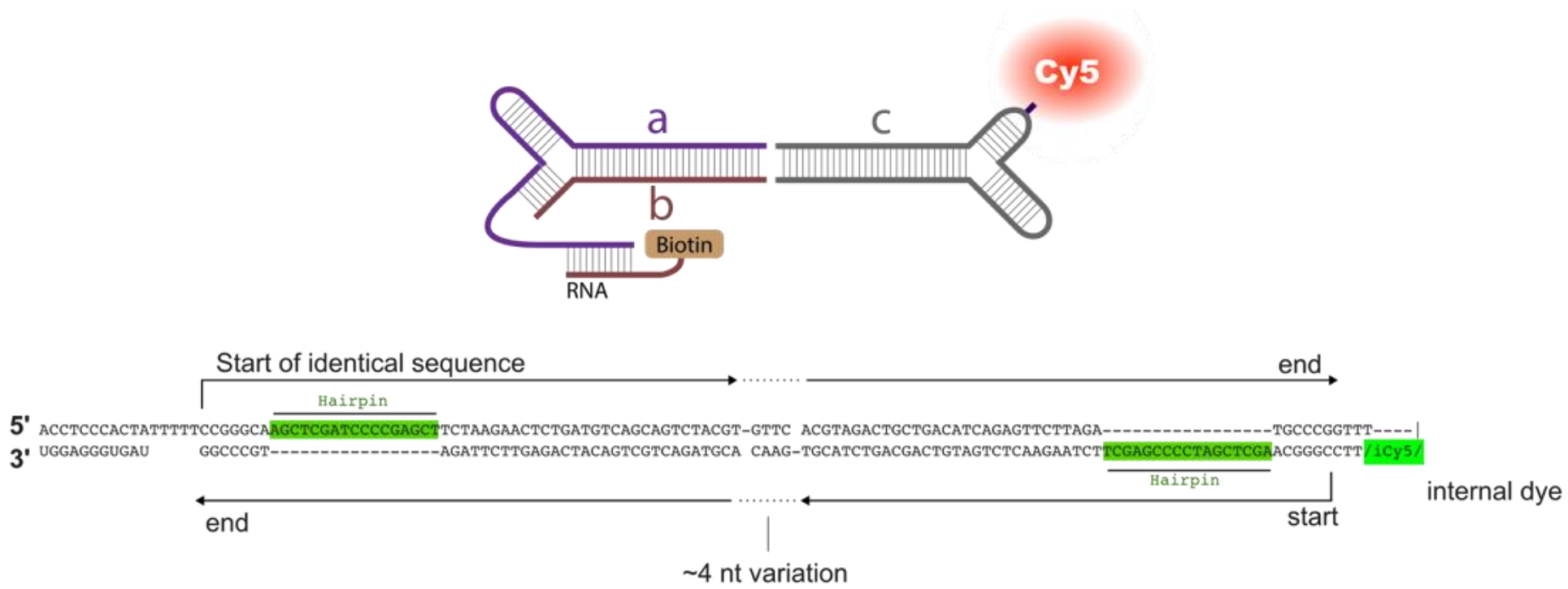

Fig. 2 for reviewers – DNA substrate design. Top shows overall shape after annealing (adapted from Fig. 4A) and bottom shows sequence specific information.

Although redesigning the substrates to ameliorate these issues is possible, this will lead to issues that impact complex assembly, which would decouple the results of the activity assays with the structural analysis. Removing the secondary structural elements will lead to symmetrical loading of Ku on the DNA and extending the duplex portion of DNA may lead to multiple Ku loading onto the substrate as reported in PMID 39058590. Furthermore, in order to attain yields of product amenable to sequencing, we would have to scale up the reaction by at least an order of magnitude, which would likely result in concentration related affects in complex assembly, particularly if the DNA substrate design was substantially changed.

We have carefully examined the gel diagrams and proposed products, and we have changed the labeling of figures 5A (previously 6A) and 5C to label the proper products. Additionally, we note the doublet at the fully ligated species is absent in assays with blunt end DNA (Fig. S7), and only seen with substrates containing overhangs, which would be more prone to degradation, which in combination with LigIV's ability to ligate across gaps could account for -1 nt species that would run as a doublet. We agree with the reviewer that sequencing would clarify these points, but because are limited by technical issues we have been careful about the claims we make regarding these data.

6.) Figures 4 and 5 provide little to no new information on the activities of Pol λ and PNKP, respectively. These figures are useful in confirming the activity of the proteins used in the study, though the data seems more appropriate for supplemental information than main text figures given the previous characterization of their activities.

We agree with the reviewer that some of substrate specific conclusions have been previously reported, and that showing all of these results dilutes the novel information in our study. We have consolidated these figures into a single figure (Fig 4), which demonstrates the activity of the reassembled complexes. We include the rest of the panels in Fig S9.

Reviewer #3 (Remarks to the Author):

The authors reported a short-range synapse cryo-EM complex that contains Polλ-BRCT, Ku70/80, LigIV, XRCCC4, XPF, and PAXX. They observed interesting conformations showing

that LigIV-DBD adopts an open conformation and Poλ-BRCT-NTR interacts with the Ku70/80 and LigIV-BRCT domains. They carried out a series of biochemical experiments to show that polλ facilitates repair of gap and overhang substrates even in the presence of DNAPKcs, while PNKP facilitates repair of 3'-Pho and 5'-OH moieties. The combination of both Polλ and PNKP in the SR or LR complex facilitates repair of both lesions. Overall, the experiments are logically executed, the manuscript is well written, and the results provide a wealth of architectural and structural data with which prior and ongoing experiments should be reconciled.

Before publication the authors should consider the following points.

We thank the reviewer for their positive comments regarding our manuscript.

Points.

1. There is an apparent disconnection between the new structure and biochemical experiments. While structure explains how Polλ-BRCT-NTR binds the SR complex, it does not generate a hypothesis to test in the biochemical experiments. For example, Polλ-BRCT has been shown to interact with Ku-70/80 in a recent cryo-EM structure. Authors also point out the BRCT domains between polλ, polμ, and TdT bind at nearly identical sites. The difference of these polymerases could come from the difference in their NTR, for which the authors here identified a new NTR interaction with LigIV-BRCT. Could this interaction be tested and shown that the interface between Polλ-NTR and LigIV-BRCT is critical for repair? If feasible, a comparison with Polμ and TdT would be a strong addition to the manuscript.

We thank the reviewer for raising these points regarding the connection of the biochemical work to the structural work, as well as the relevance of the Pol X-NTR-LigIV-BRCT1 interaction in the repair process. We agree with the reviewer that the significance of the NTR was not addressed in our biochemical experiments since these were included mainly to show the correlation between short-range complex assembly and activity as well expand the model of end-processing to include PNKP, which was elusive in our structural investigation.

In order to better characterize the Pol X-NTR interaction we carried out fluorescence polarization between the LigIV-BRCT1 domain and the NTR of Pol λ, Pol μ, and TdT. However, no binding was observed in any of these experiments, indicating that this interaction is extremely weak on its own, most likely dependent on the Pol X-BRCT binding to Ku70/80. Further supporting this notion is that in the crosslinking mass spectroscopy experiments performed during revisions, we observed crosslinking between Pol λ and LigIV at this interface only in the presence of the other NHEJ factors, and not in a sample only containing X4L4 and the polymerase.

2. Although the in vitro ligation system the authors generated has the advantage of obtaining a stable and homogenous NHEJ complex, it may not be able to capture a transient interaction component, especially if the mutation/deletion study is performed. Were each of the biochemical studies supported by the PAGE gel? If so, these should be included. If not the limitations should be noted.

We thank the reviewer for their careful analysis of our experimental setup, and we agree that it precludes interrogation of transient interactions. We did not perform parallel PAGE analysis of the proteins for each condition assayed biochemically and have added the following text to the manuscript to acknowledge this at lines 267-269:

"We note that while this approach is particularly effective at capturing strongly interacting species, transient interactions that may also contribute to repair are likely to be excluded from the analysis."

3. There is a lot of interesting new structural information and analysis, which likely informs prior studies and future efforts that merits more discussion. In this regard, the authors should clarify where their results support or extend the recent cryo-EM structure of Polλ-BRCT with Ku and LigIV published this year (https://www.nature.com/articles/s41467-025-59133-2). Are these results consistent with the kinetic mechanism and evident role of lnRNA in the ligation complex shown by single-molecule kinetics (Wang JL, et al.. Dissection of DNA double-strand-break repair using novel single-molecule forceps. Nat Struct Mol Biol. 2018 Jun;25(6):482-487. doi: 10.1038/s41594-018-0065-1; Thapar et al., Mechanism of efficient double-strand break repair by a long non-coding RNA. Nucleic Acids Res. 2020 48:10953-10972. doi: 10.1093/nar/gkaa784)? How may these results change or support the analyses of DNA-PKcs sequence conservation (Lees-Miller et al., Uncovering DNA-PKcs ancient phylogeny, unique sequence motifs and insights for human disease. Prog Biophys Mol Biol. 2021 163:87-108. doi: 10.1016/j.pbiomolbio.2020.09.010), XLF regulation of the XRCC4· ligase IV complex (Hammel et al., XLF regulates filament architecture of the XRCC4·ligase IV complex. Structure. 2010;18:1431-42. doi: 10.1016/j.str.2010.09.009), and the authors' prior review (Williams et al., Structural insights into NHEJ: building up an integrated picture of the dynamic DSB repair super complex, one component and interaction at a time. DNA Repair (Amst). 2014 17:110-20. doi: 10.1016/j.dnarep.2014.02.009)?

We thank the reviewer for highlighting areas where we can extend our discussion and better situate our contributions in the field. To show where our results interact with studies, we have added the following text to the manuscript at lines 422-444:

"Interestingly, although these authors report structures with X4L4 bound to the DNA-PK-Pol λ complex, they did not observe the Pol λ NTR-LigIV BRCT1 interaction. Because the XLF-X4L4 scaffold has not been assembled in this long-range synaptic state, the torsional strain required to rotate the LigIV-BRCT domains observed during short-range synapsis is absent. This may support our speculation that a critical determinant in the polymerase's NTR interaction is transition to short-range complex. Previous studies have also shown significant variability in both the positioning of XLF and XRCC4 relative to one another when forming the XXL (XLF-XRCC4-LigIV) scaffold, as well as the ability of the CTR of the XRCC4 coiled-coil region to fold back on itself under certain conditions[46,47]. This indicates some level of dynamism in this region, which is particularly intriguing given the XL-MS data showing significant interaction between Pol λ and XRCC4 near this site. We also highlight that this interaction, along with the Ku70 CTR-LigIV DBD interaction add to an expanding list of small interfaces and weak

interactions between different NHEJ factors mediatic synapsis and repair, in line with single molecule studies showing demonstrating how the dynamic range of repair is quantitatively modulated by the presence or absence of different NHEJ factors[48]. Although we show that cryo-EM, particularly in conjunction with emerging structure prediction technologies, is effective at identifying and characterizing some of these interactions, there are undoubtedly limits to this strategy, and whether these approaches will prove useful in interrogating the effects of less ordered species such as certain lncRNAs that have been identified as NHEJ modulators[49] remains an open question."

Because we observe dATP stimulated phosphorylation at the PQR phosphorylation cluster, we have added the following at lines 314-318:

"The results show that DNA-PK is catalytically active and able to support DNA enhanced autophosphorylation in the presence of either ATP or dATP on serine 2056, a highly conserved member of the PQR phosphorylation cluster of DNA-PKcs[35]."

REVIEWERS' COMMENTS

Reviewer #1 (Remarks to the Author):

The authors have made substantial efforts to revise the manuscript and have addressed most of my previous concerns. However, two issues remain:

1. For Fig. 1 in the rebuttal letter, the authors should provide the full gel image with the bottom portion clearly visible. This is important because, in some cases (e.g., with Ku and PNKP), the intact substrate band appears fainter than in the control when proteins are included in the reaction. This raises the possibility that shorter products may be present at the bottom of the gel or may have run off the gel.
To address this concern, the authors should quantify the band intensities (e.g., grayscale analysis) to determine whether there is any reduction in the total input substrate. While this issue may not critically affect the overall conclusions, providing quantitative analysis would strengthen the interpretation and ensure that differences between conditions are not due to unequal substrate loading or loss.
Additionally, the authors state in several places in the rebuttal letter that quantification is not important. However, when comparing different experimental conditions, quantitative data provide clearer and more reliable support for conclusions. Without such analysis, it remains difficult to exclude the possibility that variations arise from differences in input material. Nevertheless, as noted by the authors, this issue may not significantly alter the main conclusions in this manuscript.

We thank the reviewer for their detailed attention to our rebuttal. Please find the uncropped gel image below. We see no evidence for any products of degradation in the bottom half of the gel.

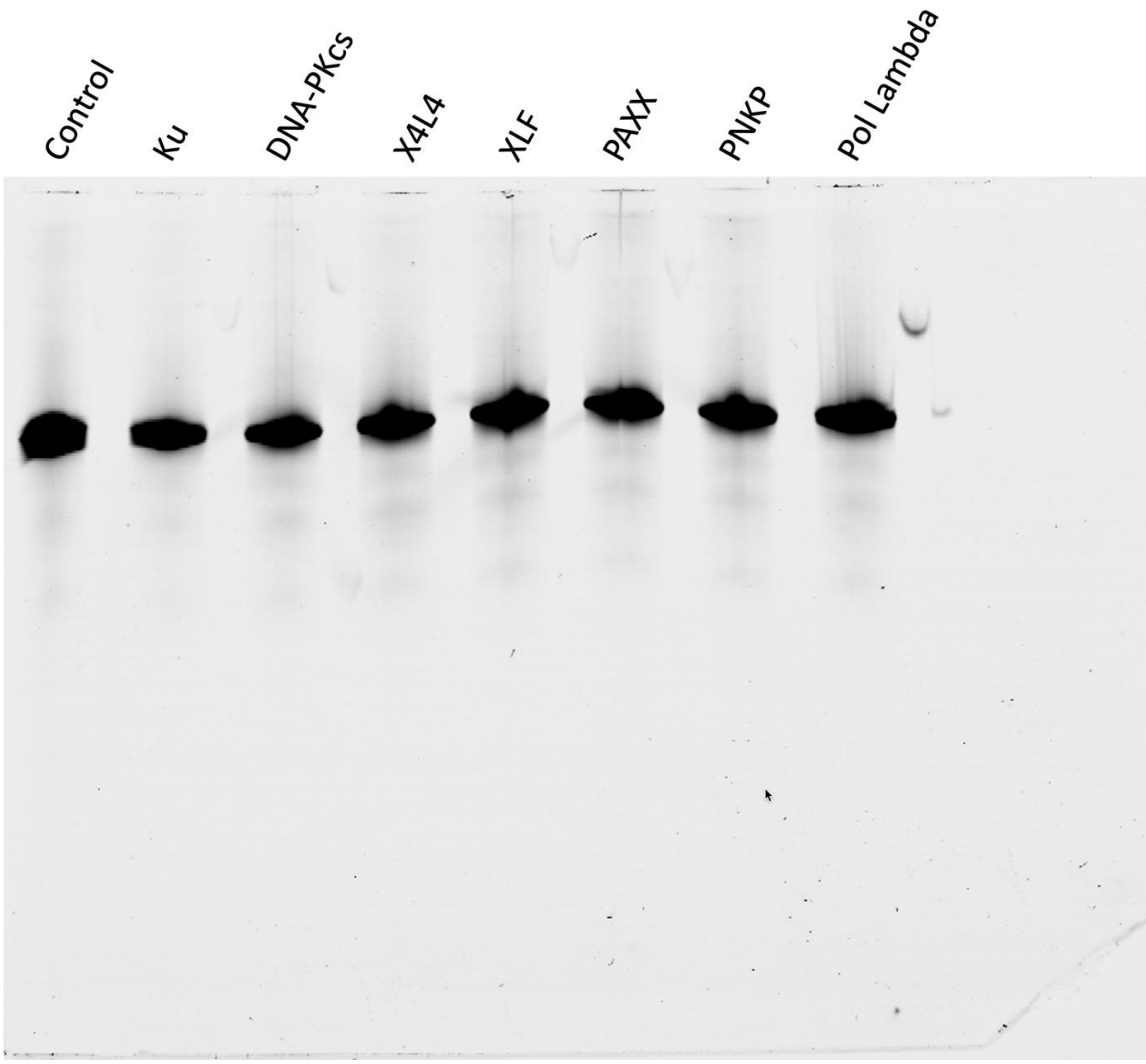

Although we cannot rule out that no very small fragments ran off of the gel, it was run for a relatively short time, and we would expect a variety of smaller DNA species in the case of nuclease contamination, particularly since the fluorescent label is internal to the oligonucleotide, approximately equidistant from the 3’ and 5’ ends.

We agree with the reviewer that extensive quantifications of all activity assays would add to the manuscript. However, we are reticent to make this addition to the main text given the limitation of two biological replicates for many of the conditions and the journal’s explicit guidance regarding the matter. As the reviewer notes, we have been careful to avoid quantitative claims, and we feel that the addition of these analyses would not substantially change our conclusions.

2. In point 8, the authors state that sequencing of repair junctions is technically challenging due to the absence of suitable primer sites. However, this limitation can be overcome by amplifying the repair products, followed by TA cloning into a plasmid vector. The cloned fragments can then be propagated in E. coli and subjected to Sanger sequencing.

Regarding the authors' claim (lines 297–304) that Pol λ incorporates mismatched nucleotides leading to double-ligated products, this conclusion is not fully supported in the absence of junction sequence data. An alternative explanation—such as gap-crossing ligation—could also account for the observed products. Therefore, the authors should at least acknowledge this possibility and revise their wording accordingly to avoid overinterpretation.

We agree with the reviewer that we cannot rule out some amount of gap-crossing ligation in the absence of sequencing information. We still strongly favor the misincorporation of nucleotides as an explanation given that we don't observe gap-crossing ligation on similar substrates in Figs. 5D-E, in addition to the results given in Supplementary Fig. 7C.We have added the following text to the manuscript to acknowledge this alternative explanation:

"Alternatively, it is possible that once one strand is repaired, LigIV becomes more capable of gap crossing ligation, which also be consistent with the presence of all ligation products observed in these lanes, although results on similar substrates in Figs. 5D-E do not show evidence of robust gap-crossing ligation after repair on the opposite strand."

Reviewer #2 (Remarks to the Author):

The revision of the manuscript by Vogt et al is improved by changing the structure of the figures as well as the addition of XL-MS data. The authors were able to address most of my major concerns. The result is a much more rigorous manuscript albeit still relatively modest scientific advance.

We thank the reviewer for acknowledging our efforts, as well as their initial comments, which have led to a substantially improved manuscript.

Reviewer #3 (Remarks to the Author):

The revised manuscript by Vogt et al. presents a compelling structural and biochemical investigation into the coordination of Pol λ and PNKP within the NHEJ supercomplex. By determining the cryo-EM structure of Pol λ bound to the SR synaptic complex and complementing this with XL-MS and biochemical assays, the authors provide a key snapshot of how accessory factors access DSBs without compromising the integrity of the core repair scaffold. In my view, the authors have provided a suitable rebuttal, adding significant experimental data that successfully addresses previous critiques.
A notable result is the structural elucidation of Pol λ's recruitment. While the BRCT domain interaction with Ku70/80 was anticipated, identification of a novel interaction between the Pol λ NTR and the LigIV-BRCT1 domain is a significant advance. Capturing the LigIV catalytic domain in an "open" conformation on incompatible DNA ends provides a vital structural intermediate. This nicely explains how the DNA ends become physically accessible to processing polymerases while the complex remains tethered by the LigIV DNA-binding domain (DBD). The results successfully bridge structural modeling with functional readouts. The use of a pulldown-based ligation assay with specifically designed chemically blocked or gapped substrates convincingly shows the sequential, coordinated action of Pol λ and PNKP within the intact SR complex. This data is broadly significant for DNA repair and structural biology. It

reveals the fundamental dynamic nature of NHEJ and complements established literature. The authors improved their analyses to synergize with contemporaneously published works (e.g., Frit et al., 2025; Liu et al., 2025), positioning their SR-Pol λ structure as a complementary, distinct step in the repair process.

The authors' experimental revisions are exemplary. The original manuscript relied heavily on AlphaFold3 predictions for novel interfaces (such as the Pol NTR-LigIV interaction). The authors have responded to reviews by doing XL-MS, which provided orthogonal, empirical evidence for these contacts. Furthermore, they improved comments regarding gap-crossing ligation and PAXX redundancy, aligning their statements with their in vitro system data. They provided clear scientifically sound justifications for technical limitations (e.g., why direct sequencing of the hairpin-containing ligation products was not feasible).The new data (XL-MS, fluorescence polarization, negative stain EM of pull-downs, and DNA-PKcs phosphorylation assays) nicely support the improved conclusions. By explicitly differentiating between cryo-EM-derived density and AlphaFold3-assisted modeling (including visual blurring of predicted regions in the figures), the authors ensure the evidence directly supports their structural analyses. The overall global resolution of the cryo-EM map (7.23 Å) is modest but expected for an asymmetric, multi-megadalton complex with highly flexible accessory domains. The use of localized refinement (achieving ~3.8–4.5 Å) completely justifies their rigid-body docking and modeling. Their interpretation of the in vitro biochemical data is appropriately measured and avoids over-quantification of inherently variable pulldown assays. The authors also rightly note that their pulldown system captures stable endpoints.

The combination of state-of-the-art structural biology (GraFix, local refinement, XL-MS) with tailored biochemical assays yields a high-quality, comprehensive advance. The methodology is robust and adheres to modern structural biology standards, including proper validation metrics for cryo-EM (Table 2) and mass spectrometry. The methods section details protein expression/purification, complex assembly, GraFix stabilization, and single-particle processing pipelines with sufficient granularity for reproduction. The mechanistic insights into how cancer-relevant DNA repair pathways maintain fidelity will appeal to a broad audience of biochemists, structural biologists, and oncologists. No further experimental evidence is reasonably required for publication.

We thank the reviewer for their positive comments and appraisal of our work.
